# Supplementary material for: Incidence and influencing factors of occupational pneumoconiosis: a systematic review and meta-analysis
Source: BMJ Open. 2023 Mar 1;13(3):e065114. doi: 10.1136/bmjopen-2022-065114 (PMC9980323; doi:10.1136/bmjopen-2022-065114)
Supplement: Supplementary data [file bmjopen-2022-065114supp008.pdf]

Search strategy of each online database

Online database	Search number	Search strategy
PubMed	#1	Silicosis [MeSH Terms] OR anthracosis [MeSH Terms] OR anthracosilicosis [MeSH Terms] OR berylliosis [MeSH Terms] OR byssinosis [MeSH Terms] OR siderosis [MeSH Terms] OR Caplan syndrome [MeSH Terms]
	#2	Pneumoconios*[Title/Abstract] OR pneumokoniosis [Title/Abstract] OR asbestos*[Title/Abstract] OR silicosis [Title/Abstract] OR anthracosis [Title/Abstract] OR anthracosilicosis [Title/Abstract] OR berylliosis [Title/Abstract] OR byssinosis [Title/Abstract] OR siderosis [Title/Abstract] OR "Caplan syndrome"[Title/Abstract] OR “Caplan’s syndrome” [Title/Abstract] OR aluminosis [Title/Abstract] OR baritosis [Title/Abstract] OR chalicosis [Title/Abstract] OR “occupational lung disease*” [Title/Abstract] OR “dust disease*” [Title/Abstract]
	#3	(Silica[Title/Abstract] OR asbestos[Title/Abstract] OR cotton[Title/Abstract] OR

		bagasse[Title/Abstract] OR iron[Title/Abstract] OR anthracite[Title/Abstract] OR stone[Title/Abstract] OR barium [Title/Abstract] OR aluminum[Title/Abstract] OR beryllium[Title/Abstract] OR "hard metal"[Title/Abstract]) AND (dust*[Title/Abstract] OR particle*[Title/Abstract]) AND (lung[Title/Abstract] OR pleura*[Title/Abstract] OR "respiratory tract"[Title/Abstract] OR pulmonary[Title/Abstract] OR fibrosis[Title/Abstract])
	#4	#1 OR #2 OR #3
	#5	“Cohort studies” [MeSH Terms] OR “longitudinal studies” [MeSH Terms] OR “follow up studies” [MeSH Terms] OR “prospective studies” [MeSH Terms] OR “retrospective studies” [MeSH Terms]
	#6	“Cohort studies” [Title/Abstract] OR “longitudinal studies” [Title/Abstract] OR “follow up studies” [Title/Abstract] OR “prospective studies” [Title/Abstract] OR “retrospective studies” [Title/Abstract]
	#7	#5 OR #6

	#8	#4 AND #7
Cochrane Library	#1	(pneumoconios*):ti,ab,kw OR (pneumokoniosis):ti,ab,kw OR (asbestos*):ti,ab,kw OR (silicosis):ti,ab,kw OR (anthracosis):ti,ab,kw OR (anthracosilicosis):ti,ab,kw OR (berylliosis):ti,ab,kw OR (byssinosis):ti,ab,kw OR (siderosis):ti,ab,kw OR ("caplan syndrome"):ti,ab,kw OR ("caplan's syndrome"):ti,ab,kw OR (aluminosis):ti,ab,kw OR (baritosis):ti,ab,kw OR (chalicosis):ti,ab,kw OR ("occupational lung disease*"):ti,ab,kw OR ("dust disease*"):ti,ab,kw
	#2	MeSH descriptor: [Asbestosis] explode all trees
	#3	MeSH descriptor: [Silicosis] explode all trees
	#4	MeSH descriptor: [Anthracosis] explode all trees
	#5	MeSH descriptor: [Anthracosilicosis] explode all trees
	#6	MeSH descriptor: [Berylliosis] explode all trees
	#7	MeSH descriptor: [Byssinosis] explode all trees
	#8	MeSH descriptor: [Siderosis] explode all trees
	#9	MeSH descriptor: [Caplan Syndrome] explode all trees

	#10	#2 OR #3 OR #4 OR #5 OR #6 OR #7 OR #8
	#11	(silica):ti,ab,kw OR (asbestos):ti,ab,kw OR (cotton):ti,ab,kw OR (bagasse):ti,ab,kw OR (iron):ti,ab,kw OR (anthracite):ti,ab,kw OR (stone):ti,ab,kw OR (barium):ti,ab,kw OR (aluminium):ti,ab,kw OR (beryllium):ti,ab,kw OR ("hard metal"):ti,ab,kw
	#12	(dust*):ti,ab,kw OR (particle*):ti,ab,kw
	#13	(lung):ti,ab,kw OR (pleura*):ti,ab,kw OR ("respiratory tract"):ti,ab,kw OR (pulmonary):ti,ab,kw OR (fibrosis):ti,ab,kw
	#14	#11 AND #12 AND #13
	#15	#1 OR #10 OR #14
	#16	("cohort studies"):ti,ab,kw OR ("longitudinal studies"):ti,ab,kw OR ("follow up studies"):ti,ab,kw OR ("prospective studies"):ti,ab,kw OR ("retrospective studies"):ti,ab,kw
	#17	MeSH descriptor: [Retrospective Studies] explode all trees
	#18	MeSH descriptor: [Prospective Studies] explode all trees

	#19	MeSH descriptor: [Follow-Up Studies] explode all trees
	#20	MeSH descriptor: [Longitudinal Studies] explode all trees
	#21	MeSH descriptor: [Cohort Studies] explode all trees
	#22	#16 OR #17 OR #18 OR #19 OR #21
	#23	#15 AND #22
Web of Science	#1	TS = (Pneumoconios* OR pneumokoniosis OR asbestos* OR silicosis OR anthracosis OR anthracosilicosis OR berylliosis OR byssinosis OR siderosis OR Caplan* syndrome OR aluminosis OR baritosis OR chalicosis OR occupational lung disease* OR dust disease*)
	#2	TS = (Silica OR asbestos OR cotton OR bagasse OR iron OR anthracite OR stone OR barium OR aluminum OR beryllium OR hard metal)
	#3	TS = (dust* OR particle*)
	#4	TS = (lung OR pleura* OR respiratory tract OR pulmonary OR fibrosis)
	#5	#2 AND #3 AND #4
	#6	#1 OR #5
	#7	TS = (cohort studies OR longitudinal studies OR

		follow up studies OR prospective studies OR retrospective studies)
	#8	#6 AND #7
Embase	#1	'siderosis'/exp OR 'occupational lung disease'/exp
	#2	pneumoconios*: ab,ti OR pneumokoniosis: ab,ti OR asbestos*: ab,ti OR silicosis: ab,ti OR anthracosis: ab,ti OR anthracosilicosis: ab,ti OR berylliosis: ab,ti OR byssinosis: ab,ti OR siderosis: ab,ti OR 'caplan* syndrome ': ab,ti OR aluminosis: ab,ti OR baritosis: ab,ti OR chalicosis: ab,ti OR 'occupational lung disease* ': ab,ti OR 'dust disease* ': ab,ti
	#3	silica: ab,ti OR asbestos: ab,ti OR cotton: ab,ti OR bagasse: ab,ti OR iron: ab,ti OR anthracite: ab,ti OR stone: ab,ti OR barium: ab,ti OR aluminum: ab,ti OR beryllium: ab,ti OR 'hard metal ': ab,ti
	#4	dust*: ab,ti OR particle*: ab,ti
	#5	lung: ab,ti OR pleura*: ab,ti OR 'respiratory tract ': ab,ti OR pulmonary: ab,ti OR fibrosis: ab,ti
	#6	#3 AND #4 AND #5
	#7	#1 OR #2 OR #6
	#8	'longitudinal study'/exp OR 'cohort analysis'/exp OR

		'prospective study'/exp OR 'retrospective study'/exp
	#9	'cohort studies': ab,ti OR 'longitudinal studies': ab,ti OR 'follow up studies': ab,ti OR 'prospective studies': ab,ti OR 'retrospective studies': ab,ti
	#10	#8 OR #9
	#11	#7 AND #10
