# Supplementary figures and images for: Incidence and influencing factors of occupational pneumoconiosis: a systematic review and meta-analysis
Source: BMJ Open. 2023 Mar 1;13(3):e065114. doi: 10.1136/bmjopen-2022-065114 (PMC9980323; doi:10.1136/bmjopen-2022-065114)

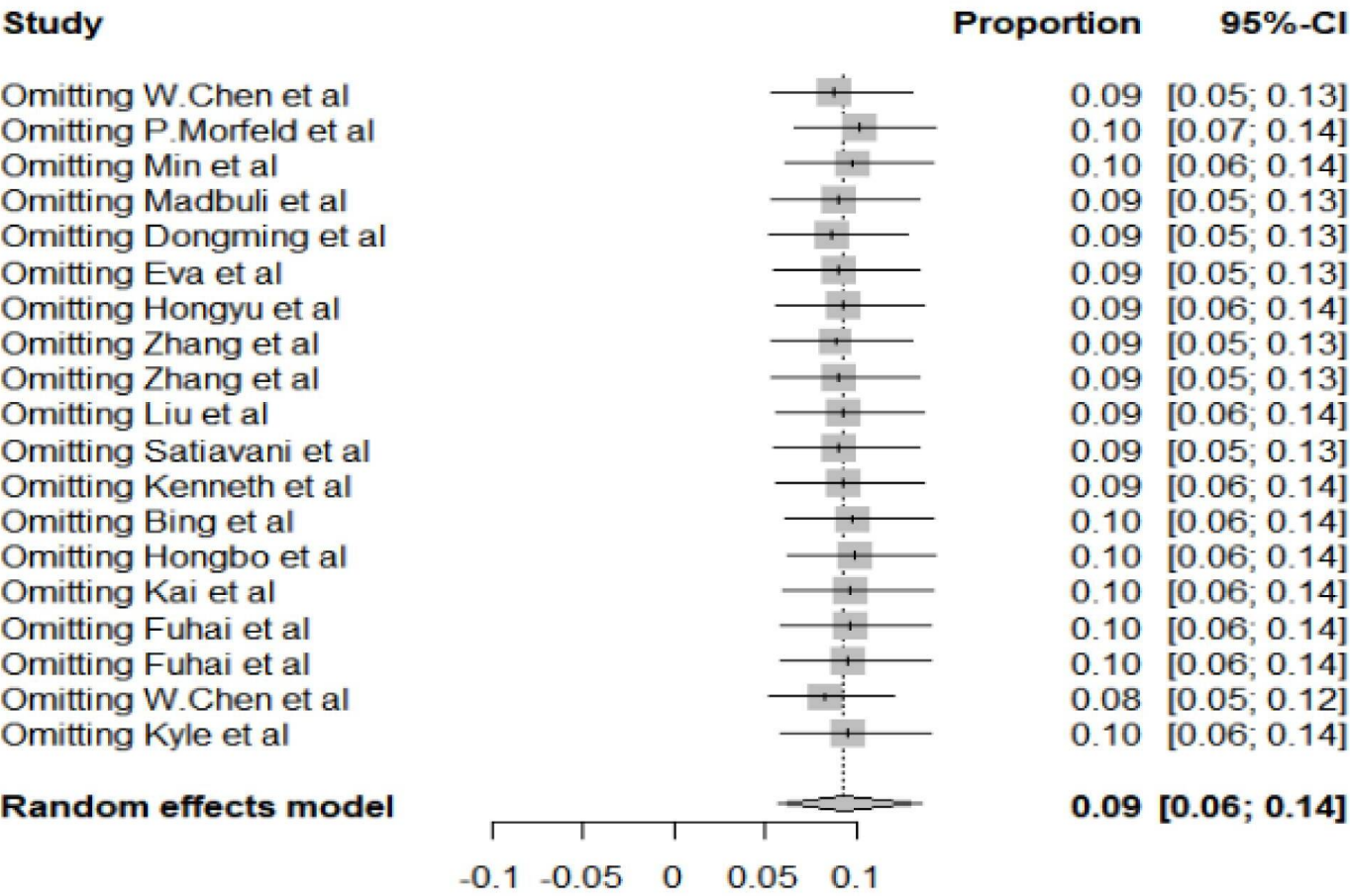

Supplement: Supplementary data [file bmjopen-2022-065114supp001.pdf]

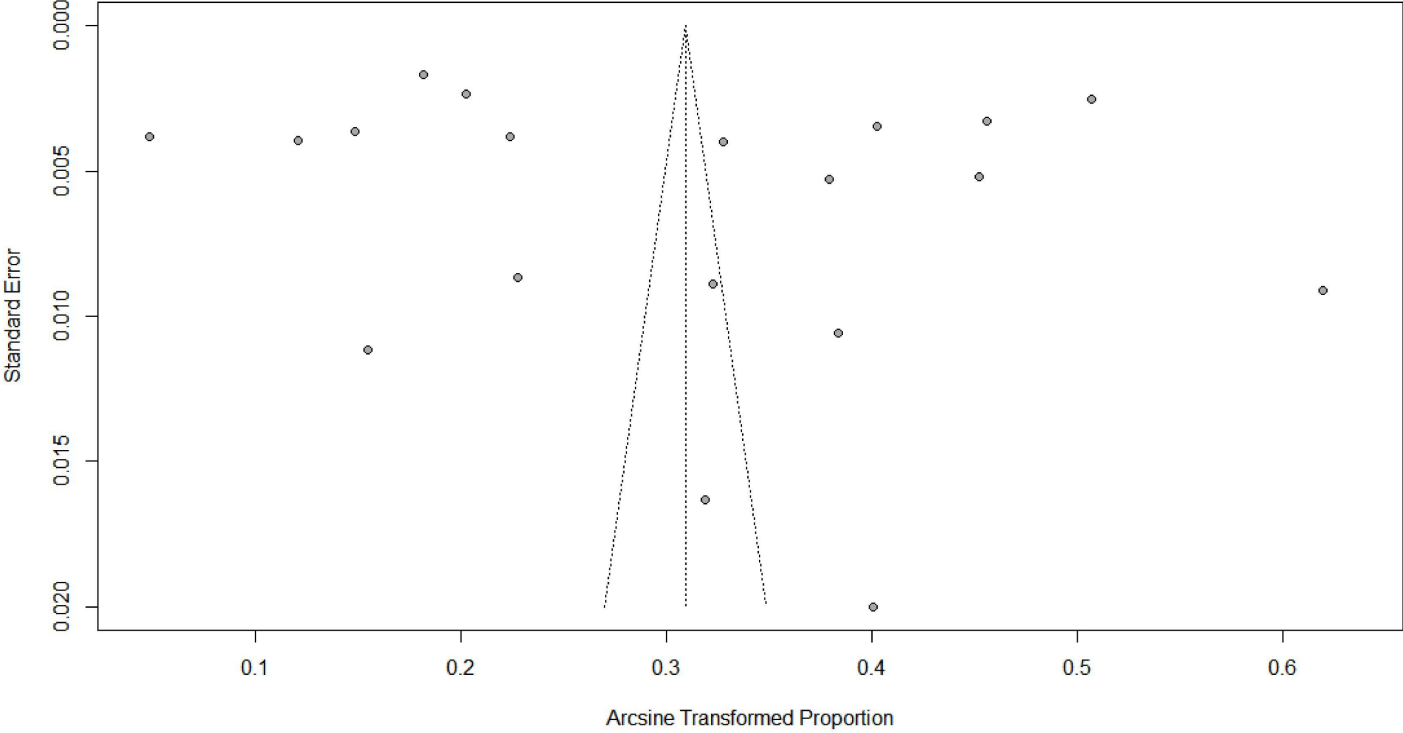

Supplement: Supplementary data [file bmjopen-2022-065114supp002.pdf]

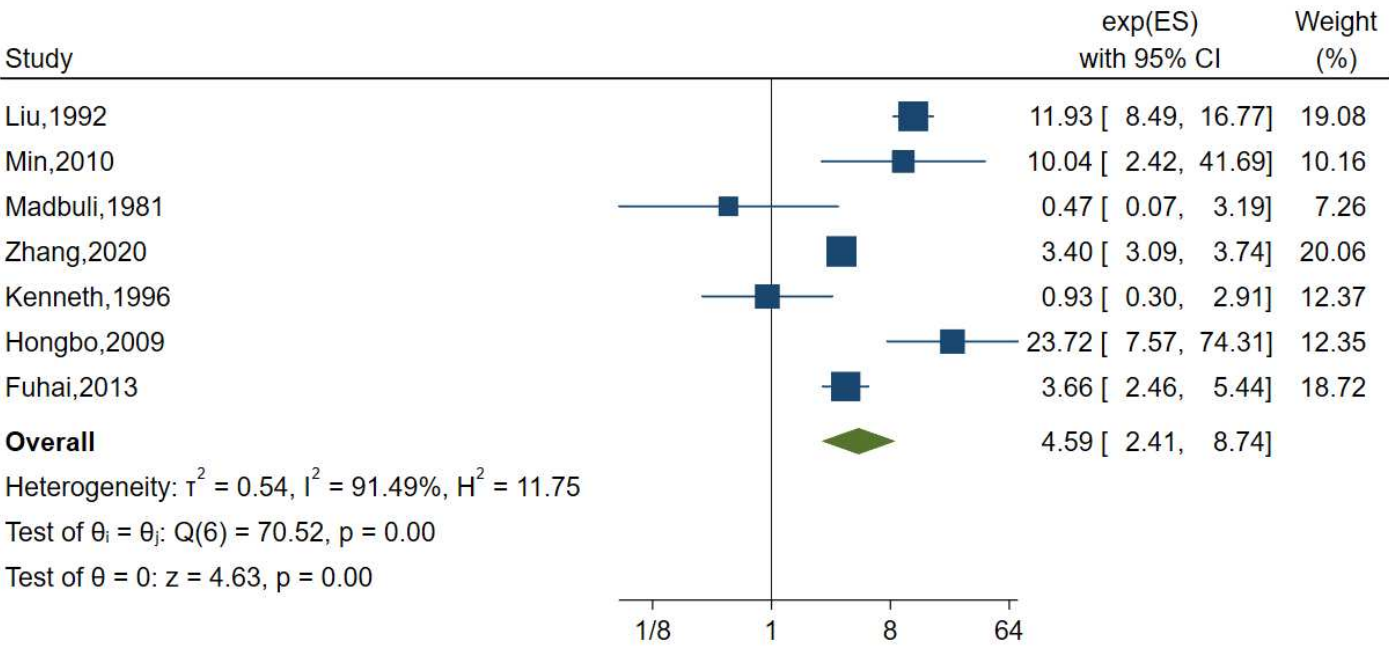

Random-effects DerSimonian-Laird model

Supplement: Supplementary data [file bmjopen-2022-065114supp003.pdf]

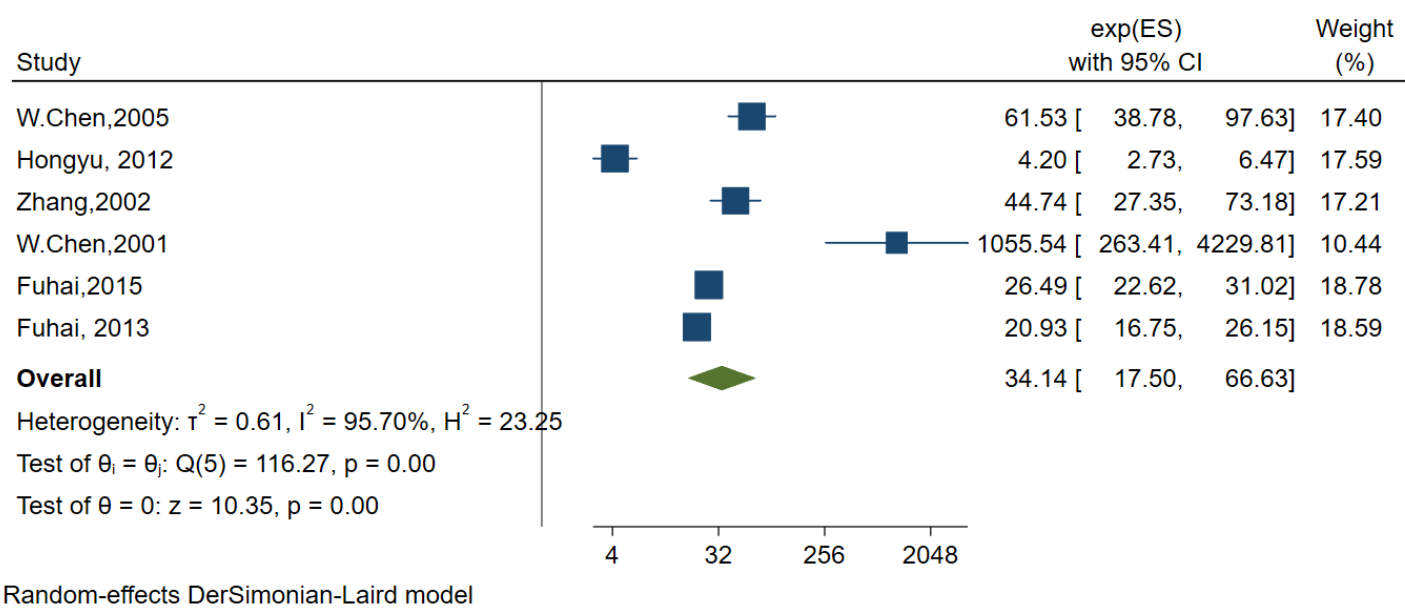

Supplement: Supplementary data [file bmjopen-2022-065114supp004.pdf]

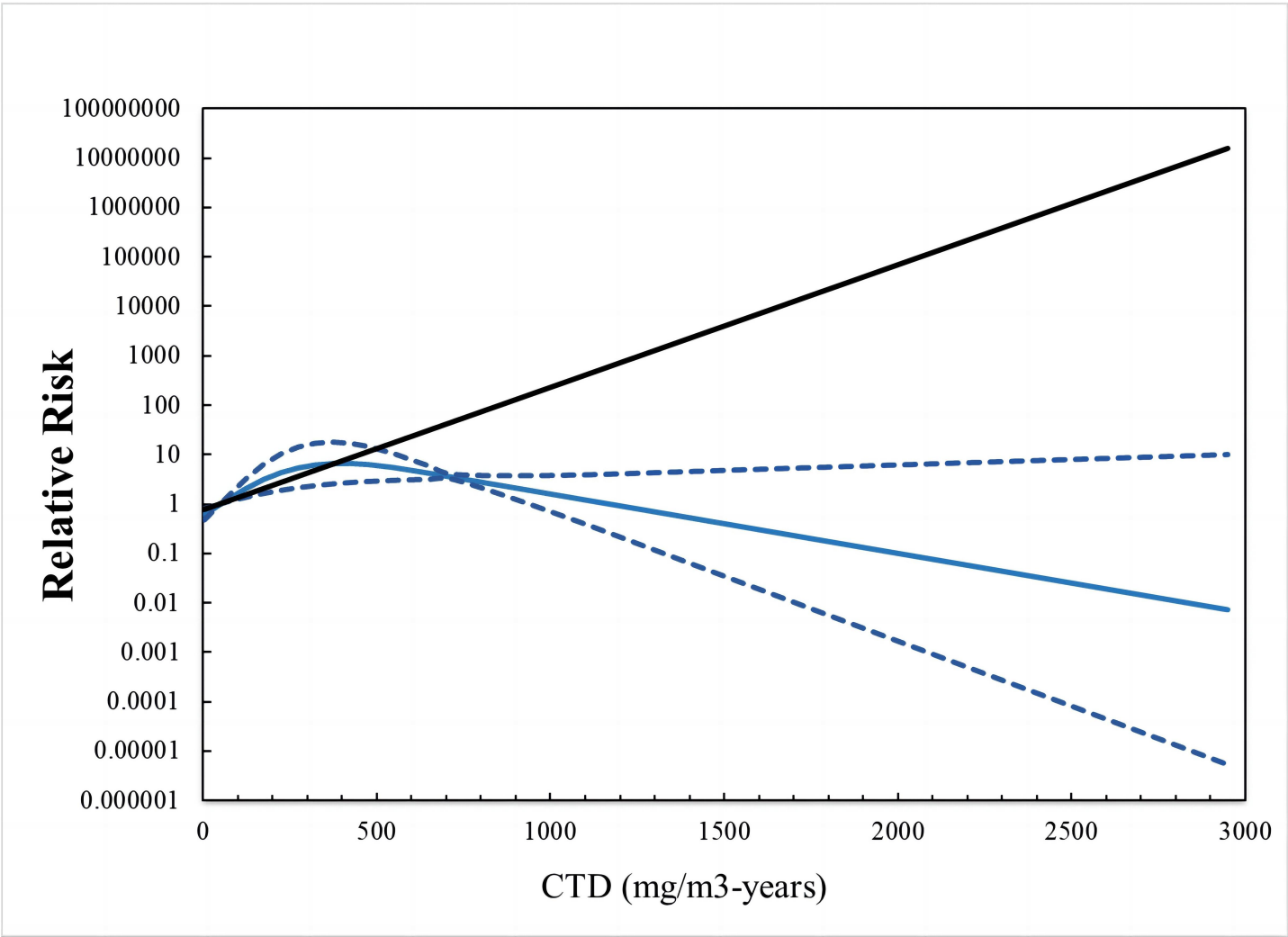

Supplement: Supplementary data [file bmjopen-2022-065114supp005.pdf]

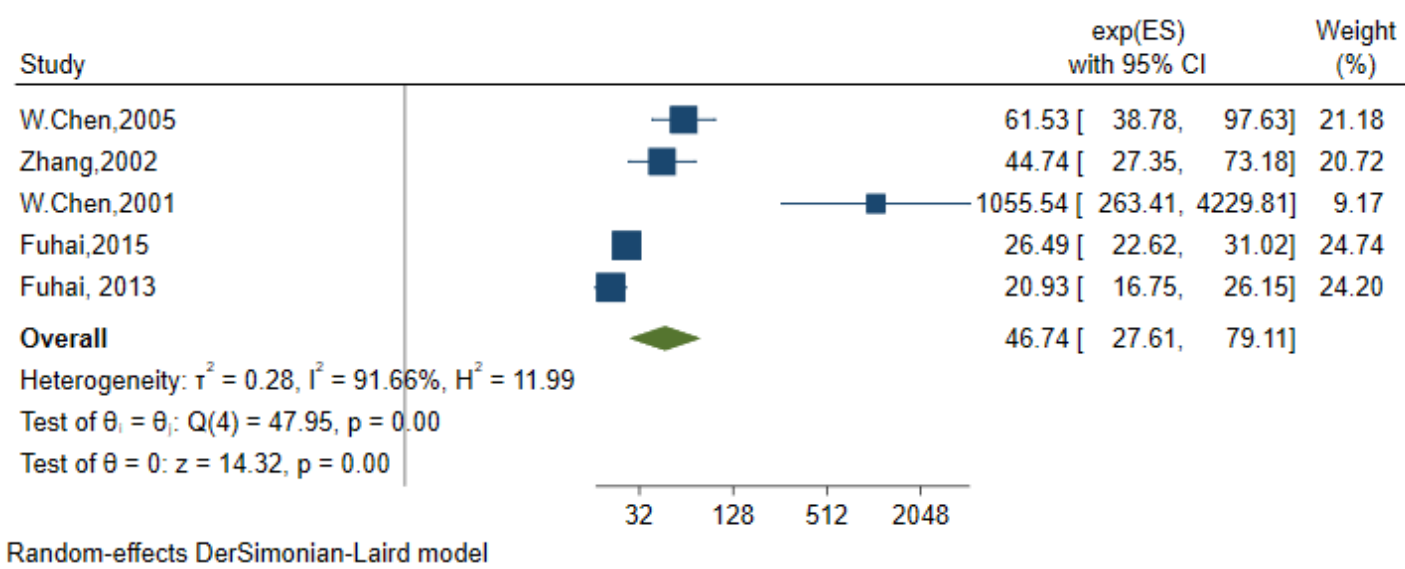

Supplement: Supplementary data [file bmjopen-2022-065114supp006.pdf]

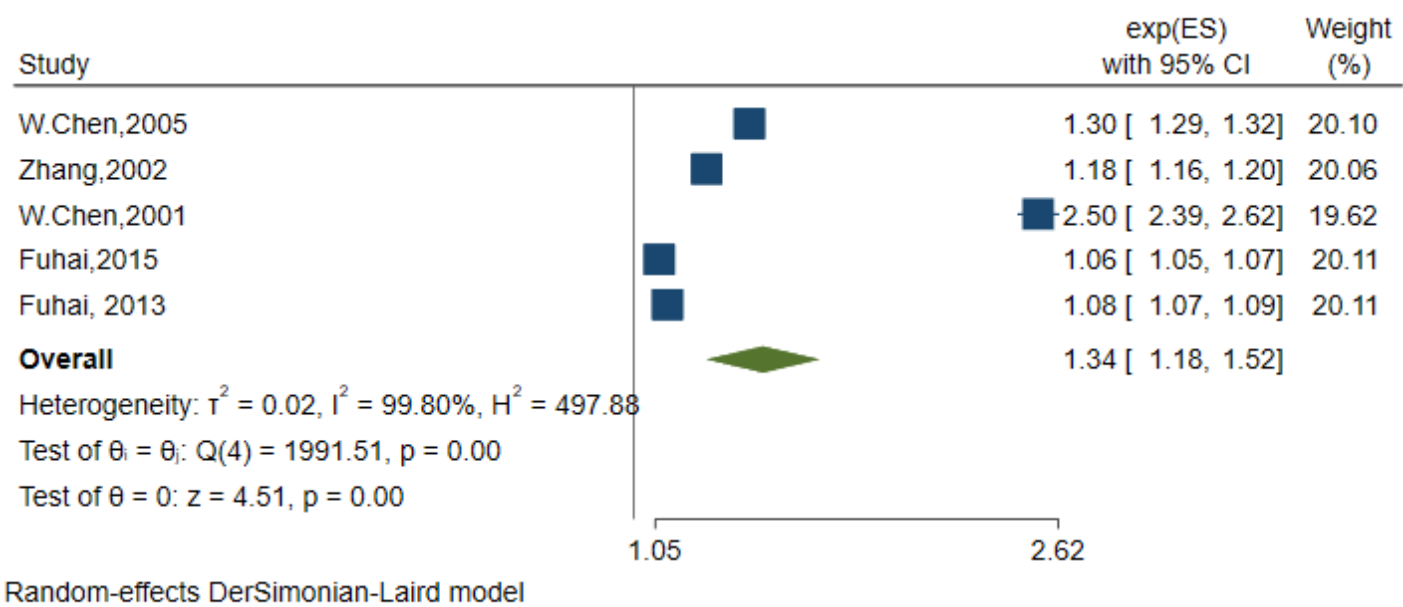

Supplement: Supplementary data [file bmjopen-2022-065114supp007.pdf]
