# Supplementary material for: Incidence and influencing factors of occupational pneumoconiosis: a systematic review and meta-analysis
Source: BMJ Open. 2023 Mar 1;13(3):e065114. doi: 10.1136/bmjopen-2022-065114 (PMC9980323; doi:10.1136/bmjopen-2022-065114)
Supplement: Author's manuscript [file bmjopen-2022-065114.draft_revisions.pdf]

BMJ Open is committed to open peer review. As part of this commitment we make the peer review history of every article we publish publicly available.

When an article is published we post the peer reviewers' comments and the authors' responses online. We also post the versions of the paper that were used during peer review. These are the versions that the peer review comments apply to.

The versions of the paper that follow are the versions that were submitted during the peer review process. They are not the versions of record or the final published versions. They should not be cited or distributed as the published version of this manuscript.

BMJ Open is an open access journal and the full, final, typeset and author-corrected version of record of the manuscript is available on our site with no access controls, subscription charges or pay-per-view fees (http://bmjopen.bmj.com).

If you have any questions on BMJ Open's open peer review process please email info.bmjopen@bmj.com

BMJ Open

Prevalence and influencing factors of occupational pneumoconiosis: A systematic review and meta-analysis.

Journal:	*BMJ Open*
Manuscript ID	bmjopen-2022-065114
Article Type:	Original research
Date Submitted by the Author:	26-May-2022
Complete List of Authors:	Su, Xuesen; Shanxi Medical University,<br>Kong, Xiaomei; First Hospital of Shanxi Medical University, Department of Respiratory and Critical Care Medicine; The National Health Commission Key Laboratory of Pneumoconiosis (Shanxi, China) Project<br>Yu, xiao; First Hospital of Shanxi Medical University, Department of Respiratory and Critical Care Medicine; The National Health Commission Key Laboratory of Pneumoconiosis (Shanxi, China) Project<br>Zhang, Xinri; First Hospital of Shanxi Medical University, Department of Respiratory and Critical Care Medicine; National Health Commission Key Laboratory of Pneumoconiosis (Shanxi China) Project
Keywords:	OCCUPATIONAL & INDUSTRIAL MEDICINE, PUBLIC HEALTH, EPIDEMIOLOGY

SCHOLARONE™
Manuscripts

I, the Submitting Author has the right to grant and does grant on behalf of all authors of the Work (as defined in the below author licence), an exclusive licence and/or a non-exclusive licence for contributions from authors who are: i) UK Crown employees; ii) where BMJ has agreed a CC-BY licence shall apply, and/or iii) in accordance with the terms applicable for US Federal Government officers or employees acting as part of their official duties; on a worldwide, perpetual, irrevocable, royalty-free basis to BMJ Publishing Group Ltd ("BMJ") its licensees and where the relevant Journal is co-owned by BMJ to the co-owners of the Journal, to publish the Work in this journal and any other BMJ products and to exploit all rights, as set out in our licence.

The Submitting Author accepts and understands that any supply made under these terms is made by BMJ to the Submitting Author unless you are acting as an employee on behalf of your employer or a postgraduate student of an affiliated institution which is paying any applicable article publishing charge ("APC") for Open Access articles. Where the Submitting Author wishes to make the Work available on an Open Access basis (and intends to pay the relevant APC), the terms of reuse of such Open Access shall be governed by a Creative Commons licence – details of these licences and which Creative Commons licence will apply to this Work are set out in our licence referred to above.

Other than as permitted in any relevant BMJ Author's Self Archiving Policies, I confirm this Work has not been accepted for publication elsewhere, is not being considered for publication elsewhere and does not duplicate material already published. I confirm all authors consent to publication of this Work and authorise the granting of this licence.

For peer review only

Prevalence and influencing factors of occupational pneumoconiosis: A systematic review and meta-analysis.

Xuesen Su[1, 3], Xiaomei Kong[2, 3], Xiao Yu[2, 3], Xinri Zhang[2, 3]

Institutional Affiliations:

[1] The First College for Clinical Medicine, Shanxi Medical University, No. 56 Xinjian South Road, Taiyuan, Shanxi, People's Republic of China.

[2] Department of Respiratory and Critical Care Medicine, First Hospital of Shanxi Medical University, Shanxi Medical University, No. 85 Jiefang South Road, Taiyuan, Shanxi, People's Republic of China.

[3] The National Health Commission Key Laboratory of Pneumoconiosis (Shanxi, China) Project, No. 85 Jiefang South Road, Taiyuan, Shanxi, People's Republic of China.

Corresponding author: Xinri Zhang, M.D.

Department of Respiratory and Critical Care Medicine, First Hospital of Shanxi Medical University, Shanxi Medical University, Taiyuan, Shanxi, People's Republic of China.

E-mail address: XinriZhangsxmd@163.com

For peer review only - http://bmjopen.bmj.com/site/about/guidelines.xhtml

Abstract:

Objectives: To estimate the prevalence and incidence of pneumoconiosis worldwide and explore influencing factors of it.

Design: Systematic review and meta-analysis.

Setting: Cohort studies on occupational pneumoconiosis.

Participants: PubMed, Embase, the Cochrane Library and Web of Science were searched. Studies were selected for meta-analysis if they involved at least one variable investigated as an influencing factor for incidence of pneumoconiosis and reported either the parameters and CIs of the risk fit to the data, or sufficient information to facilitate calculation of those values. Finally, 19 studies met the inclusion and exclusion criteria and were included in our meta-analysis, involving 335,424 participants, of whom 29,972 developed pneumoconiosis.

Primary outcome measures: The pooled prevalence of pneumoconiosis and risk ratio (RR) and 95% confidence intervals (CIs) of influencing factors.

Results: The pooled prevalence of pneumoconiosis was 0.093 (95% CI: 0.085~0.135). We found some influencing factors: (1) male (RR=3.74; 95%CI 1.31~10.64; P=0.01), (2) smoking (RR=1.80; 95%CI 1.34~2.43; P=0.0001), (3) tunneling category (RR=4.75; 95%CI 1.96~11.53; P<0.0001), (4) helping category (RR=0.07; 95%CI 0.13~0.16; P<0.0001), (5) duration of dust exposure ((RR=4.59, 95% CIs 2.41~8.74, P<0.01), (6) cumulative total dust exposure (CTD) (RR=34.14, 95% CIs 17.50~66.63, P<0.01). A dose-response analysis showed a significant positively linear dose-response association between prevalence of pneumoconiosis

For peer review only - http://bmjopen.bmj.com/site/about/guidelines.xhtml

and duration of exposure and CTD (P-nonlinearity =0.10, P-nonlinearity =0.16; respectively).

Conclusion: The prevalence of pneumoconiosis in occupational workers was 0.093 and seven factors were associated with the prevalence, which are able to provide some clues for targeted prevention of pneumoconiosis.

PROSPERO registration number: CRD42022323233.

Keywords: pneumoconiosis, influencing factors, systematic review, meta-analysis

Abbreviations: NOS: Newcastle Ottawa Scale, CWP: Coal Worker's Pneumoconiosis, CTD: cumulative total dust exposure, CSE: cumulative silica exposure.

For peer review only

For peer review only - http://bmjopen.bmj.com/site/about/guidelines.xhtml

Article Summary

- The pooled prevalence of pneumoconiosis among dust exposed workers was 0.093 (95% CI: 0.085~0.135)
- Seven factors influencing morbidity of pneumoconiosis, including gender, age, smoking, coal workers' occupational categories, duration of dust exposure, CTD and CSE.
- There is a linear dose-response association between risk of pneumoconiosis and level of dust exposure.
- The incidence of silicosis was highly collinear with CSE (r=0.794, P<0.001), and an additional 1 mg/m^3-years of CSE causes an increase in incidence of silicosis by 2.19% (95%CI: 1.00%~4.86%).

Strengths and limitations of this study

- To our knowledge, this is the first systematic review and meta-analysis to estimate the prevalence of pneumoconiosis in dust exposed workers by pooling data from only cohort studies.
- Our study provides quantitative relation between influencing factors and pneumoconiosis with a lager sample size.
- Considering the limited literature available, some potentially relevant risk factors were not included in this meta-analysis, such as alcohol drinking and tuberculosis.
- Significant heterogeneity in the pooled results for many reasons and the small number of included studies may limit the reliability of our results for some risk

For peer review only - http://bmjopen.bmj.com/site/about/guidelines.xhtml

factors.

Introduction

Pneumoconiosis is inclusive of a group of serious pulmonary diseases associated with the inhalation of mineral dusts and corresponding reactions of lung tissues, including diffuse fibrosis and progressive lung dysfunction. The prevalence, risk factors, diagnosis at the early stages, prevention and intervention of pneumoconiosis among workers exposed to dust have received much attention worldwide, but established therapeutic options for pneumoconiosis are limited at present, underscoring the importance of safeguard procedure for it[1].

Many measures have been put in place to protect workers against dust inhalation, such as methods of controlling dust concentration, assessment of exposure, using tools to prevent exposure, regular physical examination, and pathological records preservation, and eventually some studies have reported a downward trend since 2015[2-5]—around 527,500 cases of prevalence with about 60,000 new patients reported globally in 2017 corresponds with around 1,111,000 cases of prevalence with about 99,000 new patients reported globally in 2016. However, it is still escalating in some regions, especially countries with a large number of workers[6 7]. Additionally, there are some worse outcomes for some special types of workers. For instance, Satiavani et al[8] observed that there was a more rapid growth in prevalence and cumulative risk of silicosis increasing years of smoking and dust exposure than never-smoking workers in a 14 years cohort study, suggesting stricter exposure limits and more preventive measure may need to be imposed on smoking workers. So, the possible reason for

For peer review only - http://bmjopen.bmj.com/site/about/guidelines.xhtml

current status is that present preventive standards may not be able to protect workers of highly different characteristics, in another word, some targeted prevention may be much in demand now.

Targeted prevention requires a further understanding of the influencing factors. Despite some previous studies have shown that the incidence rate of pneumoconiosis was affected by many factors, such as advanced age, male, smoking and dust exposure[8-10], limited prospective data and the homogeneity of studied populations impede their clinical applicability. Furthermore, there is no meta-analysis and systematic review that summary related research results. Consequently, risk factors of pneumoconiosis and the shape of relation between them remain relatively unclear.

Due to the scarce and controversial available evidence, we conducted the current systematic review and meta-analysis to give some clues for targeted prevention of pneumoconiosis. The aim of this systematic review was to address and explore the prevalence and the impact of different influencing factors on the morbidity of pneumoconiosis and the shape of relation between them with a lager sample size.

Methods

This meta-analysis was completed in accordance with the Preferred Reporting Items for Systematic Reviews and Meta-Analysis (PRISMA) statement[11] and was registered with PROSPERO (Registration NO: CRD42022323233).

Search strategy

Two reviewers systematically and independently searched the literature published before November 2021 in online databases, including PubMed, EMBASE, the

For peer review only - http://bmjopen.bmj.com/site/about/guidelines.xhtml

Cochrane Library and Web of science, with no limitation on language or time of publication by using search terms related to “pneumoconiosis” and “cohort studies”. Any differences in the literature search process were resolved through a full discussion with a third reviewer. *Supplementary Table* provides details of the search terms for each electronic database.

Inclusion and exclusion criteria

Two reviewers independently exported all studies identified by the search strategy to Endnote X9 (Thomson Reuters, Philadelphia, USA). Titles and abstracts of all search results were screened first, and then the full text of eligible literature was further examined independently. Studies included in this meta-analysis must meet the following criteria: (1) all involving occupational dust exposure workers (exposed to occupational dust in any period of their work history) are divided into with-pneumoconiosis group and without-pneumoconiosis group according to the national diagnostic criteria of pneumoconiosis; (2) at least one variable investigated as an influencing factor for morbidity of pneumoconiosis; (3) the outcome was the number of workers with and without pneumoconiosis under the condition of with and without exposure or with different levels of exposure (4) cohort study designs. The following studies were excluded: (1) studies with insufficient data; (2) the diagnosis of pneumoconiosis patients included in studies wasn’t reported. Any disagreements about inclusion of eligible papers were resolved through a full discussion with a third reviewer.

Quality assessment

For peer review only - http://bmjopen.bmj.com/site/about/guidelines.xhtml

The quality of the included studies was assessed independently by two authors using the Newcastle Ottawa Scale (NOS). The NOS contains eight categories relating to methodological quality and each study was given an eventual score out of a maximum of 9 points. A score of 0~6 points equated to a low-quality study, and a score of 7~9 points equated to a high-quality study. Any disagreements about quality assessment were resolved through a full discussion with a third reviewer.

Data extraction

Two reviewers independently conducted data extraction and any disagreements about data extraction were resolved through a full discussion with a third reviewer. The following data were extracted: (1) study characteristics: first author name, publication year, country, duration of follow-up, duration of follow-up, sample size; (2) type of pneumoconiosis included; (3) participant numbers (separating those who developed pneumoconiosis and those who did not); (4) characteristics of these groups (such as gender); (5) exposure of interest (duration or dust concentrations); (6)information about the outcome of interest (incidence of pneumoconiosis); (7) risk estimates with 95% CIs from any statistic model.

Statistical methods

Statistical analyses were undertaken using Statistical Package for the Social Sciences (SPSS, Version 25.0, IBM SPSS Statistics; IBM Corporation, Armonk, NY), Stata 16.1 software (Stata Corp. TX, 2019) and R 4.1.3 statistical software (Schwarzer, 2007; TUNA Team, Tsinghua University, 2022). First of all, for exploring the prevalence of pneumoconiosis in dust exposed workers, the pooled prevalence and its

For peer review only - http://bmjopen.bmj.com/site/about/guidelines.xhtml

95% confidence intervals (CIs) were calculated by using a random-effect model. Second, in the study of the association between potentially influencing factors and prevalence of pneumoconiosis, dichotomous variable was reported as frequency and proportion, the risk ratios (RRs) and its 95% CIs were summarized to evaluate the outcome by using a random-effect model. Third, we conducted highest categories versus lowest categories and dose-response meta-analyses to explored the relation between risk of pneumoconiosis and dust exposure including two aspects: duration of dust exposure and cumulative total dust exposure concentration (CTD).

Firstly, we applied a random effect model to summarize RRs and 95% CIs from each study obtained by comparing the highest versus the lowest exposure category. Then, the method described by Greenland[12] and Orsini[13] was applied for the dose-response analysis, requiring that the distribution of cases, non-cases, and the RRs with the 95% CI for at least three quantitative exposure categories are known. For each exposure category, we extracted midpoint as the dose in each category, estimated by calculating the mean of the lower and upper bound. When the highest and lowest categories were open ended, we assumed the length of these open-ended intervals to be the same as those of the adjacent intervals.

A two stage, random effects dose-response meta-analysis was applied to examine a possible non-linear association between risk of pneumoconiosis and two aspects of dust exposure. This meta-analysis was done through modelling of dust exposure and restricted cubic splines with three knots at fixed centiles of 10%, 50%, and 90% of the distribution. Based on the Orsini method, we calculated restricted cubic spline models

For peer review only - http://bmjopen.bmj.com/site/about/guidelines.xhtml

by a generalized least squares trend estimation method, which takes into account the correlation within each set of reported relative risks or hazard ratios. Furthermore, we pooled study-specific estimates using the restricted maximum likelihood method in a random-effects meta-analysis. A probability value for non-linearity was estimated by null hypothesis testing, in which the coefficient of the second spline was considered equal to zero. A linear dose-response association between risk of pneumoconiosis and additional five years of duration and 50 mg/m^3-years of CTD was investigated by use of the two-stage generalized least squares trend estimation method. Study specific slope lines were first estimated and then these lines were combined to obtain an overall average slope by a random effects model.

The effect of cumulative silica exposure (CSE) on incidence of silicosis was estimated using the Pearson correlation and simple linear regression model. Due to the incidence rate of silicosis was non-normally distributed data (the higher the incidence, the smaller number of group), the natural logarithm of incidence rate was used as the normal distribution variable. So, the linear model has the following form for the association between them:

$Y = ln\ (Y')$

$Y = a + b*X$

where Y' is the value of incidence rate (cases of silicosis/total workers), a is intercept of the linear regression model, b is the corresponding parameter estimate for CSE and X is the value of CSE (mg/m^3*years).

We calculated both the Q statistic and I^2 as indicators of heterogeneity (significant

For peer review only - http://bmjopen.bmj.com/site/about/guidelines.xhtml

heterogeneity defined as I^2 >50%, P<0.05). Subgroup analysis were performed to explore possible reasons for the significant heterogeneity and address the effects of important factors. We divided the data into small groups based on type of pneumoconiosis (CWP versus other type of pneumoconiosis), occupational category for CWP and study quality (NOS<7 versus NOS≥7), and then compared across subgroups.

Sensitivity analysis was performed to assess the influence of any particular study on the pooled estimate by eliminating individual studies one by one. To assess the possible risk of publication biases from the included studies, we leveraged funnel plots in combination with Egger's tests.

Results

Study selection and study characteristics

A detailed flow diagram of the literature screening was presented in *Fig. 1*.5293 articles were identified in the initial search. After exclusion of duplicate papers and those that did not meet the inclusion criteria, 78 full text articles of potentially relevant studies were identified. After full text review, an additional 59 articles were excluded: forty-four studies with insufficient and duplicate research data, six that were cross-sectional studies, five that were reviews, three that were case-control studies and one that was case report. Finally,19 studies met the inclusion and exclusion criteria and were included in our meta-analysis.

Among these studies, there were six prospective studies, nine retrospective studies and four retrospective-prospective cohort studies. NOS scores ranged from 5 to 9 and

For peer review only - http://bmjopen.bmj.com/site/about/guidelines.xhtml

publish year range from 1981 to 2021.This meta-analysis involved 335,424 participants, including 29,972 pneumoconiosis patients and 305,452 non-pneumoconiosis workers. We finally analyzed seven factors influencing morbidity of pneumoconiosis, including gender, smoking, coal workers' occupational categories, duration of dust exposure, age, CTD and CSE (*Table 1*).

Table 1 Study characteristics

Study	Country	Design	Pneumoconiosis reported	Duration (years)	Sample size (pneumoconiosis cases/total cases)	Risk factors reported	NOS scores
W. Chen et ai[9]	China	Retrospective	Silicosis	45	4465/23002	F5	7
P. Morfeld et al[14]	Germany	Prospective	Silicosis	20	40/17144	F1, F2	8
Min et al[15]	China	Prospective	Silicosis	29	48/2009	F1, F2, F4, F7	9
Madbuli et al[16]	Egypt	Prospective	Byssinosis	10	95/625	F1, F4	5
Dongming et al[17]	China	Retrospective and Prospective	Silicosis	43	9377/39808	F2, F6	9
Eva et al[18]	South African	Prospective	Silicosis	23	313/2235	F6	7

For peer review only - http://bmjopen.bmj.com/site/about/guidelines.xhtml

Hongyu et al[19]	China	Retrospective and Prospective	Silicosis	44	316/3147	F1, F2, F5, F7	9
Zhang et al[20]	China	Retrospective	Silicosis	35	1769/9268	F5	6
Zhang et al[21]	China	Retrospective	CWP		3224/21000	F4	7
Liu et al[22]	China	Retrospective	CWP	7	1624/15647	F3, F4	5
Satiavan et al[8]	China	Prospective	Silicosis	54	1219/8887	F1	7
Kenneth et al[23]	U.S.	Prospective	Silicosis	5.5	92/937	F2, F4, F6	7
Bing et al[10]	China	Retrospective	CWP	48	411/18705	F3	8
Hongbo et al[24]	China	Retrospective	CWP	50	236/16154	F3, F4	8
Kai et al[6]	China	Retrospective and Prospective	CWP	44	2873/87904	F3	7
Fuhai et al[25]	China	Retrospective and Prospective	CWP	41	1847/45589	F3, F5	8
Fuhai et al[26]	China	Retrospective	CWP	42	838/17023	F3, F4, F5	9

For peer review only

For peer review only - http://bmjopen.bmj.com/site/about/guidelines.xhtml

Kyle et al[27]	U.S.	Retrospective	Silicosis	25	170/3330	F6	7
W. Chen et al[28]	China	Retrospective	Silicosis	35	1015/3010	F5	6

Risk factors: F1: gender; F2: smoking; F3: coal workers' occupational categories; F4: duration of dust exposure; F5: cumulative total dust exposure (CTD); F6: cumulative silica exposure (CSE); F7: age. CWP: Coal Worker's Pneumoconiosis

Pooled results, sensitive analysis, publication bias of the prevalence of pneumoconiosis

Based on the results of random-effects method, the pooled prevalence of pneumoconiosis among dust exposed workers was 0.093 (95% CI: 0.085~0.135), with a high-level between-study heterogeneity (I^2 = 99.93 %, P < 0.0001) (*Fig.2, Table 2*). The sensitive analysis demonstrated that there were no individual studies significantly affected the pooled results (*Fig.3*). Egger's linear regression test (t = 0.81, p = 0.427) suggested no significant publication bias and the funnel plot was presented in *Fig.4*.

Table 2 The pooled results and subgroup analysis of prevalence of pneumoconiosis

		Number of studies	n/N	Pooled prevalence rate			Heterogeneity	
				Effect size	Lower limit	Upper limit	I^2	p
Total		19	29,972/335,424	0.093	0.058	0.135	99.93%	<0.001
Pneumoconiosis	silicosis	11	18,824/112,777	0.119	0.064	0.188	99.92%	<0.001
	CWP	7	11,503/222,022	0.044	0.024	0.082	99.90%	<0.001
Gender	male	5	3,112/19,946	0.034	0.000	0.123	98.96%	<0.010
	female	5	972/12,462	0.012	0.002	0.086	98.45%	<0.010

For peer review only

For peer review only - http://bmjopen.bmj.com/site/about/guidelines.xhtml

Smoker	yes	5	4,845/35,677	0.064	0.012	0.152	99.93%	<0.001
	no	5	1,388/16,479	0.019	0.005	0.076	98.88%	<0.010
Occupational categories of coal workers	tunneling	6	3,675/36,573	0.112	0.062	0.174	99.63%	<0.010
	mining	6	1,508/21,948	0.059	0.025	0.093	99.06%	<0.010
	combining	6	2,861/42,886	0.049	0.020	0.123	99.82%	<0.001
	helping	6	765/119,854	0.005	0.001	0.019	99.56%	<0.010

Subgroup analysis of prevalence of pneumoconiosis and comparison results

Table 2 demonstrates the pooled prevalence of all subgroups stratified by different pneumoconiosis, gender, smoker or not and occupational categories of coal workers. The pooled prevalence of silicosis (0.119, 95 % CI: 0.064~0.188) was higher than that of CWP (0.044, 95 % CI: 0.024~0.082). The pooled prevalence in males (0.034, 95 % CI: 0.000~0.123) was higher than that in females (0.012 , 95 % CI: 0.002~0.086). Additionally, the pooled prevalence in smokers (0.064, 95 % CI: 0.012 ~0.152) was higher than that in non-smokers (0.019, 95 % CI: 0.005~0.076). The pooled prevalence of CWP in different occupational categories were obviously different. Specifically, the prevalence of tunneling workers was 0.112 (95%CI: 0.062~0.174), mining workers was 0.059 (95%CI: 0.025~0.093), combining workers was 0.049 (95%CI: 0.020~0.123) and helping workers was 0.005 (95%CI: 0.001~0.019).

Influencing factors of pneumoconiosis

We found seven factors influencing prevalence of pneumoconiosis, including gender, smoking, age, coal workers' occupational categories, duration of dust exposure, CTD and CSE.

For peer review only

For peer review only - http://bmjopen.bmj.com/site/about/guidelines.xhtml

Gender

Five studies, including four high quality studies and one low quality study, reported the association between gender and prevalence of pneumoconiosis. The pooled results of five studies suggested that male workers had an obvious higher risk than female (RR =3.74; 95% CI 1.31~10.64; P=0.01) with heterogeneity (P<0.00001, I^2=93%) by using a random-effect model. Some evidence of heterogeneity was detected between subgroups in stratified analyses for high quality studies (RR =4.54, 95% CI 3.70~5.57, P<0.00001) with heterogeneity (P=0.4, I^2=0%) (*Fig. 5*).

Smoking

Five studies, all of which were high quality studies, reported the comparison between smoking and never-smoking. The pooled results of five studies suggested that smoking workers had an obvious higher risk than non-smoking workers (RR =1.80; 95% CI 1.34~2.43; P=0.0001) with heterogeneity (P=0.02, I^2=67%) by using a random-effect model. No data pooling was possible for any identified subgroup (*Fig. 6*).

Age

There were two studies reporting the association between age and morbidity of pneumoconiosis. These studies[15 19] roughly suggest that the highest incidence in the age range of 50 to 60 years old. In the age range of lower than 50 years old, incidence rate increases with age, and in the age range of higher than 60 years old, it decreases with age.

Occupational category

For peer review only - http://bmjopen.bmj.com/site/about/guidelines.xhtml

Occupational categories were only reported in studies involving CWP, so we summarized result between occupational categories and incidence rate of pneumoconiosis from studies about CWP. The comparison between four different occupational categories was investigated in six studies involving 220,911 participates and 8,791 patients. According to pooled results in different occupational categories, the prevalence of tunneling workers (0.112, 95%CI: 0.062~0.174) was highest, corresponding to the lowest prevalence in helping workers (0.005,95%CI: 0.001~0.019). So, we conducted comparison between tunneling workers and other categories (mining, combining and helping workers) and between helping and other categories (tunneling, mining and combining workers). The pooled results of six studies suggested that tunneling workers had an obvious higher risk than other workers (RR =4.75; 95% CI 1.96~11.33; P<0.001) with heterogeneity (P<0.001, I^2=100%) by using a random-effect model (*Fig.7*). And helping workers had an obvious lower risk than other workers (RR =0.07; 95% CI 0.03~0.16; P<0.001) with heterogeneity (P<0.001, I^2=99%) by using a random-effect model (*Fig. 8*). Significant heterogeneity and the small number of included studies may limit the reliability of our results.

Duration of dust exposure

Seven studies, including six high quality studies and one low quality study, reported the relationship between duration of dust exposure and prevalence of pneumoconiosis. The summary effect size for pneumoconiosis morbidity comparing the longest and shortest duration of dust exposure was calculated by using random-effect model

For peer review only - http://bmjopen.bmj.com/site/about/guidelines.xhtml

(RR=4.59, 95% CIs 2.41~8.74, P<0.01; *Fig.9*), indicating a significant association; however, evidence of high heterogeneity was found between studies (I^2=91.49%, P<0.01). In dose-response analysis, there was no indication for a nonlinear association between duration of dust exposure and risk of pneumoconiosis (P-nonlinearity=0.10; *Fig.10*). Furthermore, linear dose-response meta-analysis showed that an increase in duration by 5 years was positively associated with the risk of pneumoconiosis (RR:1.26, 95%CI: 1.15 ~1.39, P<0.01; *Fig.11*) with heterogeneity (I^2=97.68%, P<0.01). No data pooling was possible for dose-response meta-analysis of any identified subgroup.

Cumulative total dust concentration (CTD)

Six studies reported the association between CTD and prevalence of pneumoconiosis, which involved 189,037 participants and 10,271 patients. The summary effect size for pneumoconiosis morbidity comparing the highest and lowest CTD was calculated by using random-effect model (RR=34.14, 95% CIs 17.50~66.63, P<0.01; *Fig.12*), indicating a significant association; however, evidence of high heterogeneity was found between studies (I^2=95.70%, P<0.01). In dose-response analysis, there was no indication for a nonlinear association between duration of dust exposure and risk of pneumoconiosis (P-nonlinearity=0.16; *Fig.13*). Furthermore, linear dose-response meta-analysis showed that an increase in CTD by 50 mg/m^3-years was positively associated with the risk of pneumoconiosis (RR:1.38, 95%CI: 1.22 ~1.55, P<0.01; *Fig.14*) with heterogeneity (I^2=99.75%, P<0.01). No data pooling was possible for dose-response meta-analysis of any identified subgroup.

For peer review only - http://bmjopen.bmj.com/site/about/guidelines.xhtml

Cumulative Silica Exposure (CSE)

Four studies reported the association between CSE and incidence of silicosis, which involved 40,212 participants and 6,371 patients. The analysis of Pearson correlation demonstrated that the incidence of silicosis was highly collinear with CSE (r=0.794, P<0.001, *Fig.15*). The corresponding estimate for value of *b* was 0.234 (95%CI: 0.131~0.337), and value of *a* was -4.053 (95%CI: -4.745~-3.362). Therefore, an additional 1 mg/m^3-years of CSE causes an increase in incidence of silicosis by $e^{0.234-4.053}$ (95%CI: $e^{0.131-4.745}$~$e^{0.337-3.362}$), that is 2.19% (95%CI: 1.00%~4.86%). No data pooling was possible for regression analysis of any identified subgroup.

Discussion

To our knowledge, this is the first systematic review and meta-analysis to estimate the prevalence of pneumoconiosis in dust exposed workers and the relationship between different influencing factors and pneumoconiosis. Our meta-analysis revealed that the pooled estimate of pneumoconiosis prevalence among dust exposed workers was about 9.3% (95% CI: 8.5%~13.5%). This estimate was higher than the prevalence of 7.5% (95% CI: 6.6%%~8.4%) reported in 2020 by an analysis from the Global Burden of Disease Study 2017[29]. The first possible reason for it was studies included by our meta-analysis mostly were from countries with relatively higher incidence rates of pneumoconiosis, such as China and American. Secondly, with the improvement of preventive measures, the incidence of pneumoconiosis is decreasing in recent years. Since the type of article included in this meta-analysis is cohort study, it is also possible that the pooled prevalence is higher than the prevalence reported by

For peer review only - http://bmjopen.bmj.com/site/about/guidelines.xhtml

recent studies. This is based on the fact that our pooled prevalence of CWP (4.4%, 95 % CI: 2.4%~8.2%) differ greatly from those of this report about CWP (1.9%, 95%CI: (1.5%~2.5%), but are close to those (3.7%, 95% CI 3.0~4.5%) of a meta-analysis for CWP among underground miners[30]. But the pooled results of only cohort studies enabled a larger sample size and accessed more to the real conclusion.

In addition, our results showed that the pooled prevalence of silicosis is higher than that of CWP, which is consistent with many previous studies. Previous studies showed that crystalline silica or quartz, the most common occupational dust, is the most important cause of silicosis through many pathologic processes[31-33], such as macrophage autophagy, autoimmune, apoptosis, and proptosis. However, the link between them and CWP is minimal, and coal's carbon content is the most critical factor in assessing CWP risk[34-36]. In other words, the incidence of silicosis is higher than that of coal worker's pneumoconiosis, possibly because the pathogenic exposures leading to silicosis are more prevalent than CWP. But the mechanism about this, enable to perfectly explain the epidemiological data, currently remains elusive.

This meta-analysis summarized several influencing factors with a larger sample size and a more comprehensive way ever before, and explored effect size of influencing factors on developing pneumoconiosis, including male, smoking, age and occupational categories.

Consistent with many previous studies[8 15 37], our findings showed that there exists a difference in the pooled prevalence of pneumoconiosis between men and women (0.034, 95 % CI: 0.000~0.123 VS 0.012, 95 % CI: 0.002~0.086), with the former

For peer review only - http://bmjopen.bmj.com/site/about/guidelines.xhtml

being at higher risk (RR =3.74; 95% CI 1.31~10.64) than the latter. On the other hand, some previous studies have shown no difference in the incidence of pneumoconiosis between men and women[38-40], however, some limitations, such as cross-sectional studies, medical examination and a short period of follow- up time, made their findings inconclusive. There are some possible reasons for the differing risk of pneumoconiosis between males and females, as follows. Frist of all, higher physical strength of labor in men contributes to more rapid pulmonary ventilation, which in turn leads to inhalation of a much higher concentration of occupational dust over the same period of time, compared to women[41 42]. Secondly, some gender disparities are largely mediated by differences in the expression of genotypes, such as differential expression of pro-inflammatory cytokines, as shown by previous gender related studies[43 44]. Thirdly, a higher percentage of male smokers compared to female smokers led to a higher risk of pneumoconiosis in the former group.

Regarding the relationship between smoking and pneumoconiosis, our finding showed the pooled prevalence in smokers (0.064, 95 % CI: 0.012~0.152) was higher than that in non-smokers (0.019, 95 % CI: 0.005~0.076), with the former being at higher risk (RR =1.80; 95% CI 1.34~2.43) than the latter. The present studies between smoking and pneumoconiosis mainly focuses on the morbidity and mortality of pneumoconiosis[17 45], and it is generally accepted that smoking can promote the occurrence of pneumoconiosis and also increase all-cause mortality in patients with pneumoconiosis despite the risk for smoking varies widely among studies. Additionally, Dongming *et al*[17] found that the incidence of pneumoconiosis increases

For peer review only - http://bmjopen.bmj.com/site/about/guidelines.xhtml

more rapidly among smokers than non-smokers, as the dust concentration increases. Previous animal research demonstrated that the tobacco smoke exposure alone had only a minimal effect on these toxicity parameters, however, the combined exposure to tobacco smoke and occupational dust exacerbated the lung response, compared to anyone alone[46]. One possible reason for it is cigarette smoking destroys the ciliated epithelium in the bronchi and decreases the pulmonary clearance of dust. Moreover, Dorman *et al*[47]. found that tobacco smoke exposure exacerbated the silica dust induced lung toxicity in the rats through changing the secretion level of several cytokines/chemokines and oxidants generated by the bronchoalveolar phagocytes. Alternatively, exposure to a toxic agent may result in gene expression changes with corresponding alterations in the functions mediated by the affected genes which may ultimately result in target organ toxicity. For instance, SPP1 – a gene involved in lung inflammatory and fibrotic responses of toxic particles, was overexpressed by 2.85-, 36.69-, and 72.61-fold, respectively, in the smoke, silica, and smoke plus silica exposed rats, compared with the air controls[48]. At present, it is highly necessary for smoking workers to quit smoking and may require lower dust concentrations and stricter prevention and control of dust inhalation than non-smokers. And the effects of tobacco smoke and occupational dust on the lungs are complex, and more researches are needed on their interactions.

There are occupational categories for any occupational dust-exposed worker, but almost all occupational studies on them have focused on coal miners[6 24 25]. This meta-analysis involved 220,911 participates and 8,791 patients of six studies. Tunneling

For peer review only - http://bmjopen.bmj.com/site/about/guidelines.xhtml

workers had highest pooled prevalence of 11.2% (95%CI: 6.2%~17.4%), mining workers were 5.9% (95%CI: 2.5%~9.3%), combining workers were 4.9% (95%CI: 2.0%~12.3%) and helping workers were 0.5% (95%CI: 0.1%~1.9%). One possible main reason for different morbidity is the average dust concentration of working area. Kai *et al*[6] found that the average dust concentration is different, and the incidence of pneumoconiosis is also different for the same occupational categories in different coal mines, that is, the higher the average dust concentration, the higher the incidence. Additionally, there are many distinctions for different occupational categories, such as working hours, work habits, and living habits. Whether these factors are also the reasons for the different incidence rates between them needs to be discovered and confirmed by future research.

The associations between risk of pneumoconiosis and duration of dust exposed and CTD were systematically assessed in this meta-analysis through comparison of the highest to the lowest categories and dose-response analyses both for linear and non-linear relationships. In the linear dose-response meta-analysis, we found a positive association between morbidity of pneumoconiosis and duration and CTD; there was no clear indication for non-linearity between them.

This is, to our knowledge, the first meta-analysis that investigated the association between duration of dust exposed and pneumoconiosis, showing a positive association. The observed positive association is consistent with results from previous clinical studies on duration and incidence of pneumoconiosis. For instance, a retrospective cohort study involving 11 state-owned coal mines found that the rate of

For peer review only - http://bmjopen.bmj.com/site/about/guidelines.xhtml

morbidity increased sharper as the length of employment[21]. Since most studies on the risk of different length of employment have been measured in increments of five years, our study also calculated the increase in risk for each additional five years (RR:1.26, 95%CI: 1.15 ~1.39). Some reviews[49-52] proposed that the development and progression of pneumoconiosis are chronic inflammatory, anti-inflammatory and fibrotic process, indicating the progression of pneumoconiosis occurs gradually under the continuous stimulation of occupational dust. So, we believe that shortening the length of employment in work environment of high dust concentration or targeted shift change of workers with long duration of dust exposed may reduce the increased morbidity as a whole. However, the effect and mechanism of these targeted preventive measures need to be further discovered and confirmed.

For CTD, although there is no similar previous meta-analysis, our results are similar to previous clinical research that revealed a positive association between incidence of pneumoconiosis and CTD[6 9 19 26]. Present studies suggested that dust is still the primary occupational hazard for developing pneumoconiosis, accordingly, if dust concentrations increase, the risk of pneumoconiosis will increase in theory[26]. We think CTD is a more significant method of evaluating exposure than average dust concentration or duration of exposure because it takes into account both them. So, we detected further risk growth beyond the increment of 50 mg/m^3-years ((RR:1.38, 95%CI: 1.22 ~1.55). However, a reduction of sample size exposed the rising level of CTD limited reliability of relation between higher level of CTD and risk of pneumoconiosis. The association between CTD and the incidence of pneumoconiosis

For peer review only - http://bmjopen.bmj.com/site/about/guidelines.xhtml

should be further investigated and analyzed, and permissible limits for CTD should be gradually imposed by the Occupational Safety and Health Administration (OSHA) in the future.

Due to lack of the effect size of silica on the risk of silicosis, we performed Pearson correlation analysis and linear regression model to estimate the effect of CSE on incidence of silicosis. Previous studies have identified the positive correlation between incidence of silicosis and silica through epidemiological studies[17 18 27]. Furthermore, Tamires *et al* found each additional year of silica exposure, the prevalence ratio of silicosis increased 4%[53]. However, CSE is a more effective way to evaluate the level of silica exposure than duration because the former takes into account it with dust concentration. There is no previous study to explore the quantitative association between CSE and incidence of silicosis because of fewer exposures' stratification in each study. In our meta-analysis, a positively strong linear correlation (r=0.794, P<0.001) was found, and more significantly we found a quantitative association—an additional 1 mg/m^3-years of CSE causes an increase in incidence of silicosis by 2.19% (95%CI: 1.00%~4.86%). At present, respirable crystalline silica (RCS) exposure limits are generally regulated in the range of 0.05 mg/m^3 to 0.1 mg/m^3 by most countries[54]. But this standard of occupational exposure is too strict to abide for most workplace, they may exceed it for short times[55]. And there is new insight are to impose more accurate assessment on dust exposure, such as measured in hours, days, weeks, months, making it more effective to control the risk

For peer review only - http://bmjopen.bmj.com/site/about/guidelines.xhtml

of pneumoconiosis[56]. We thought that our finding could provide some clues for this new insight.

Limitations

There are some inevitable limitations in our meta-analysis. First, significant heterogeneity in the pooled results for many reasons and the small number of included studies may limit the reliability of our results for some risk factors. Second, considering the limited literature available, some potentially relevant risk factors were not included in this meta-analysis, such as age, alcohol drinking and tuberculosis.

Conclusions

In summary, our pooled results of this meta-analysis identified the prevalence of pneumoconiosis in occupational workers was 0.093 and seven factors associated with the prevalence, including male, smoking, age, coal workers' occupational categories, duration of dust exposure, CTD and CSE. And we found the positive association between risk of pneumoconiosis and duration of dust exposure and CTD. Our finding will provide some clues for targeted prevention of pneumoconiosis.

Author Contributions

Xuesen Su, Xiao Yu and Xiaomei Kong completed the search, determined eligible papers for inclusion, completed the quality assessment, and data extraction. Xuesen Su, Xinri Zhang completed the meta-analysis. All authors contributed to the writing of the final manuscript.

Acknowledgements

For peer review only - http://bmjopen.bmj.com/site/about/guidelines.xhtml

The authors would also like to thank Professors from the College of Public Health, Shanxi Medical University for the advice on the study design and revise opinion.

Funding

Organization: 1.The National Health Commission Key Laboratory of Pneumoconiosis (Shanxi, China) Project; Project number: No. 2020-PT320-005; Project Leader: Xinri Zhang. 2. Key Laboratory of Respiratory Disease Prevention and Contorl of Shanxi.

Conflict of Interest

The authors have no conflict of interest to declare.

Patient and public involvement

Patients and/or the public were not involved in the design, or conduct, or reporting, or dissemination plans of this research.

Data availability statement

All data relevant to the study are included in the article or uploaded as supplementary information.

Ethics Approval

No ethical approval is necessary for this systematic review. And all the studies included in this meta-analysis have obtained ethical approval.

For peer review only - http://bmjopen.bmj.com/site/about/guidelines.xhtml

Reference

1. Qi XM, Luo Y, Song MY, et al. Pneumoconiosis: current status and future prospects. *Chinese medical journal* 2021;134(8):898-907. doi: 10.1097/cm9.0000000000001461 [published Online First: 2021/04/22]

2. Global, regional, and national incidence, prevalence, and years lived with disability for 354 diseases and injuries for 195 countries and territories, 1990-2017: a systematic analysis for the Global Burden of Disease Study 2017. *Lancet (London, England)* 2018;392(10159):1789-858. doi: 10.1016/s0140-6736(18)32279-7 [published Online First: 2018/11/30]

3. Global, regional, and national incidence, prevalence, and years lived with disability for 328 diseases and injuries for 195 countries, 1990-2016: a systematic analysis for the Global Burden of Disease Study 2016. *Lancet (London, England)* 2017;390(10100):1211-59. doi: 10.1016/s0140-6736(17)32154-2 [published Online First: 2017/09/19]

4. Global, regional, and national incidence, prevalence, and years lived with disability for 310 diseases and injuries, 1990-2015: a systematic analysis for the Global Burden of Disease Study 2015. *Lancet (London, England)* 2016;388(10053):1545-602. doi: 10.1016/s0140-6736(16)31678-6 [published Online First: 2016/10/14]

For peer review only - http://bmjopen.bmj.com/site/about/guidelines.xhtml

5. Global, regional, and national incidence, prevalence, and years lived with disability for 301 acute and chronic diseases and injuries in 188 countries, 1990-2013: a systematic analysis for the Global Burden of Disease Study 2013. *Lancet (London, England)* 2015;386(9995):743-800. doi: 10.1016/s0140-6736(15)60692-4 [published Online First: 2015/06/13]

6. Cui K, Shen F, Han B, et al. Comparison of the Cumulative Incidence Rates of Coal Workers' Pneumoconiosis between 1970 and 2013 among Four State-Owned Colliery Groups in China. *International journal of environmental research and public health* 2015;12(7):7444-56. doi: 10.3390/ijerph120707444 [published Online First: 2015/07/03]

7. Blackley DJ, Halldin CN, Laney AS. Continued Increase in Prevalence of Coal Workers' Pneumoconiosis in the United States, 1970-2017. *American journal of public health* 2018;108(9):1220-22. doi: 10.2105/ajph.2018.304517 [published Online First: 2018/07/20]

8. Poinen-Rughooputh S, Rughooputh MS, Guo Y, et al. Sex-Related Differences in the Risk of Silicosis Among Chinese Pottery Workers: A Cohort Study. *Journal of occupational and environmental medicine* 2021;63(1):74-79. doi: 10.1097/jom.0000000000002068 [published Online First: 2020/10/31]

9. Chen W, Hnizdo E, Chen JQ, et al. Risk of silicosis in cohorts of Chinese tin and tungsten miners, and pottery workers (I): an epidemiological study. *American journal of industrial medicine* 2005;48(1):1-9. doi: 10.1002/ajim.20174 [published Online First: 2005/06/09]

For peer review only - http://bmjopen.bmj.com/site/about/guidelines.xhtml

10. Han B, Liu H, Zhai G, et al. Estimates and Predictions of Coal Workers' Pneumoconiosis Cases among Redeployed Coal Workers of the Fuxin Mining Industry Group in China: A Historical Cohort Study. *PloS one* 2016;11(2):e0148179. doi: 10.1371/journal.pone.0148179 [published Online First: 2016/02/06]

11. Page MJ, McKenzie JE, Bossuyt PM, et al. The PRISMA 2020 statement: an updated guideline for reporting systematic reviews. *BMJ (Clinical research ed)* 2021;372:n71. doi: 10.1136/bmj.n71 [published Online First: 2021/03/31]

12. Greenland S, Longnecker MP. Methods for trend estimation from summarized dose-response data, with applications to meta-analysis. *American journal of epidemiology* 1992;135(11):1301-9. doi: 10.1093/oxfordjournals.aje.a116237 [published Online First: 1992/06/01]

13. Orsini N, Li R, Wolk A, et al. Meta-analysis for linear and nonlinear dose-response relations: examples, an evaluation of approximations, and software. *American journal of epidemiology* 2012;175(1):66-73. doi: 10.1093/aje/kwr265 [published Online First: 2011/12/03]

14. Morfeld P, Mundt KA, Taeger D, et al. Threshold value estimation for respirable quartz dust exposure and silicosis incidence among workers in the german porcelain industry. *Journal of occupational and environmental medicine* 2013;55(9):1027-34. doi: 10.1097/JOM.0b013e318297327a [published Online First: 2013/08/24]

15. Zhang M, Zheng YD, Du XY, et al. Silicosis in automobile foundry workers: a 29-year cohort study. *Biomedical and environmental sciences : BES* 2010;23(2):121-9. doi: 10.1016/s0895-3988(10)60041-4 [published Online First: 2010/06/03]

For peer review only - http://bmjopen.bmj.com/site/about/guidelines.xhtml

16. Noweir MH. Some observations on epidemiologic studies in Egyptian gins, cotton pressing plants and cottonseed oil extraction plants. *Chest* 1981;79(4 Suppl):15s-20s. doi: 10.1378/chest.79.4_supplement.15s [published Online First: 1981/04/01]

17. Wang D, Zhou M, Liu Y, et al. Comparison of Risk of Silicosis in Metal Mines and Pottery Factories: A 44-Year Cohort Study. *Chest* 2020;158(3):1050-59. doi: 10.1016/j.chest.2020.03.054

18. Hnizdo E, Sluis-Cremer GK. Risk of silicosis in a cohort of white South African gold miners. *American journal of industrial medicine* 1993;24(4):447-57. doi: 10.1002/ajim.4700240409 [published Online First: 1993/10/01]

19. Guan HY, Zhang H, Su LP, et al. [The study of the characteristics and influencing factors of pneumoconiosis among workers exposed to dusts in an iron mine]. *Zhonghua lao dong wei sheng zhi ye bing za zhi = Zhonghua laodong weisheng zhiyebing zazhi = Chinese journal of industrial hygiene and occupational diseases* 2012;30(1):36-40. [published Online First: 2012/06/27]

20. Zhang H, Yang J, Qin Y, et al. [Assessment of risk for tin mine dust in Guangxi]. *Zhonghua lao dong wei sheng zhi ye bing za zhi = Zhonghua laodong weisheng zhiyebing zazhi = Chinese journal of industrial hygiene and occupational diseases* 2002;20(6):430-2. [published Online First: 2003/12/26]

21. Zhang G, Wang XT. [Dose-response relationship analysis between cumulative coal dust exposure and pneumoconiosis risk]. *Zhonghua lao dong wei sheng zhi ye bing za zhi = Zhonghua laodong weisheng zhiyebing zazhi = Chinese journal of industrial hygiene and occupational diseases* 2020;38(6):433-37. doi: 10.3760/cma.j.cn121094-

For peer review only - http://bmjopen.bmj.com/site/about/guidelines.xhtml

20190510-00194 [published Online First: 2020/07/08]

22. Liu XG. [Study on the response index of population--the prevalence degree of pneumoconiosis]. *Zhonghua yu fang yi xue za zhi [Chinese journal of preventive medicine]* 1992;26(6):342-4. [published Online First: 1992/11/01]

23. Rosenman KD, Reilly MJ, Rice C, et al. Silicosis among foundry workers. Implication for the need to revise the OSHA standard. *American journal of epidemiology* 1996;144(9):890-900. doi: 10.1093/oxfordjournals.aje.a009023 [published Online First: 1996/11/01]

24. Liu H, Tang Z, Yang Y, et al. Identification and classification of high risk groups for Coal Workers' Pneumoconiosis using an artificial neural network based on occupational histories: a retrospective cohort study. *BMC public health* 2009;9:366. doi: 10.1186/1471-2458-9-366 [published Online First: 2009/09/30]

25. Shen F, Liu H, Yuan J, et al. Cost-Effectiveness of Coal Workers' Pneumoconiosis Prevention Based on Its Predicted Incidence within the Datong Coal Mine Group in China. *PloS one* 2015;10(6):e0130958. doi: 10.1371/journal.pone.0130958 [published Online First: 2015/06/23]

26. Shen F, Yuan J, Sun Z, et al. Risk identification and prediction of coal workers' pneumoconiosis in Kailuan Colliery Group in China: a historical cohort study. *PloS one* 2013;8(12):e82181. doi: 10.1371/journal.pone.0082181 [published Online First: 2014/01/01]

27. Steenland K, Brown D. Silicosis among gold miners: exposure--response analyses and risk assessment. *American journal of public health* 1995;85(10):1372-7. doi:

For peer review only - http://bmjopen.bmj.com/site/about/guidelines.xhtml

10.2105/ajph.85.10.1372 [published Online First: 1995/10/01]

28. Chen W, Zhuang Z, Attfield MD, et al. Exposure to silica and silicosis among tin miners in China: exposure-response analyses and risk assessment. *Occupational and environmental medicine* 2001;58(1):31-7. doi: 10.1136/oem.58.1.31 [published Online First: 2000/12/19]

29. Shi P, Xing X, Xi S, et al. Trends in global, regional and national incidence of pneumoconiosis caused by different aetiologies: an analysis from the Global Burden of Disease Study 2017. *Occupational and environmental medicine* 2020;77(6):407-14. doi: 10.1136/oemed-2019-106321 [published Online First: 2020/03/20]

30. Lu C, Dasgupta P, Cameron J, et al. A systematic review and meta-analysis on international studies of prevalence, mortality and survival due to coal mine dust lung disease. *PloS one* 2021;16(8):e0255617. doi: 10.1371/journal.pone.0255617 [published Online First: 2021/08/04]

31. Tan S, Chen S. Macrophage Autophagy and Silicosis: Current Perspective and Latest Insights. *International journal of molecular sciences* 2021;22(1) doi: 10.3390/ijms22010453 [published Online First: 2021/01/21]

32. Mayeux JM, Escalante GM, Christy JM, et al. Silicosis and Silica-Induced Autoimmunity in the Diversity Outbred Mouse. *Frontiers in immunology* 2018;9:874. doi: 10.3389/fimmu.2018.00874 [published Online First: 2018/05/15]

33. Tan S, Chen S. The Mechanism and Effect of Autophagy, Apoptosis, and Pyroptosis on the Progression of Silicosis. *International journal of molecular sciences* 2021;22(15) doi: 10.3390/ijms22158110 [published Online First: 2021/08/08]

For peer review only

For peer review only - http://bmjopen.bmj.com/site/about/guidelines.xhtml

34. Huang X, Li W, Attfield MD, et al. Mapping and prediction of coal workers' pneumoconiosis with bioavailable iron content in the bituminous coals. *Environmental health perspectives* 2005;113(8):964-8. doi: 10.1289/ehp.7679 [published Online First: 2005/08/05]

35. Suarthana E, Laney AS, Storey E, et al. Coal workers' pneumoconiosis in the United States: regional differences 40 years after implementation of the 1969 Federal Coal Mine Health and Safety Act. *Occupational and environmental medicine* 2011;68(12):908-13. doi: 10.1136/oem.2010.063594 [published Online First: 2011/05/21]

36. Mo J, Wang L, Au W, et al. Prevalence of coal workers' pneumoconiosis in China: a systematic analysis of 2001-2011 studies. *International journal of hygiene and environmental health* 2014;217(1):46-51. doi: 10.1016/j.ijheh.2013.03.006 [published Online First: 2013/04/24]

37. Forastiere F, Lagorio S, Michelozzi P, et al. Silica, silicosis and lung cancer among ceramic workers: a case-referent study. *American journal of industrial medicine* 1986;10(4):363-70. doi: 10.1002/ajim.4700100404 [published Online First: 1986/01/01]

38. Rastogi SK, Gupta BN, Chandra H, et al. A study of the prevalence of respiratory morbidity among agate workers. *International archives of occupational and environmental health* 1991;63(1):21-6. doi: 10.1007/bf00406193 [published Online First: 1991/01/01]

39. Gielec L, Izycki J, Woźniak H. [Evaluation of long-term occupational exposure to dust and

For peer review only - http://bmjopen.bmj.com/site/about/guidelines.xhtml

its effect on health during production of ceramic tiles]. *Medycyna pracy* 1992;43(1):25-33. [published Online First: 1992/01/01]

40. Chaudhury N, Phatak A, Paliwal R, et al. Silicosis among agate workers at Shakarpur: An analysis of clinic-based data. *Lung India : official organ of Indian Chest Society* 2010;27(4):221-4. doi: 10.4103/0970-2113.71955 [published Online First: 2010/12/09]

41. Miller AE, MacDougall JD, Tarnopolsky MA, et al. Gender differences in strength and muscle fiber characteristics. *European journal of applied physiology and occupational physiology* 1993;66(3):254-62. doi: 10.1007/bf00235103 [published Online First: 1993/01/01]

42. Card JW, Zeldin DC. Hormonal influences on lung function and response to environmental agents: lessons from animal models of respiratory disease. *Proceedings of the American Thoracic Society* 2009;6(7):588-95. doi: 10.1513/pats.200904-020RM [published Online First: 2009/11/26]

43. An J, Ribeiro RC, Webb P, et al. Estradiol repression of tumor necrosis factor-alpha transcription requires estrogen receptor activation function-2 and is enhanced by coactivators. *Proceedings of the National Academy of Sciences of the United States of America* 1999;96(26):15161-6. doi: 10.1073/pnas.96.26.15161 [published Online First: 1999/12/28]

44. Grundboeck-Jusco J, Binette JP, Kimura A, et al. The glycosaminoglycan composition of the lung with acute and chronic pathology and in senescence. *Clinica chimica acta; international journal of clinical chemistry* 1992;208(1-2):77-84. doi: 10.1016/0009-8981(92)90023-j [published Online First: 1992/06/15]

For peer review only

45. Wang D, Yang M, Liu Y, et al. Association of Silica Dust Exposure and Cigarette Smoking With Mortality Among Mine and Pottery Workers in China. *JAMA network open* 2020;3(4):e202787. doi: 10.1001/jamanetworkopen.2020.2787 [published Online First: 2020/04/15]

46. Sager TM, Umbright CM, Mustafa GM, et al. Tobacco Smoke Exposure Exacerbated Crystalline Silica-Induced Lung Toxicity in Rats. *Toxicological sciences : an official journal of the Society of Toxicology* 2020;178(2):375-90. doi: 10.1093/toxsci/kfaa146 [published Online First: 2020/09/26]

47. Dorman DC, Mokashi V, Wagner DJ, et al. Biological responses in rats exposed to cigarette smoke and Middle East sand (dust). *Inhalation toxicology* 2012;24(2):109-24. doi: 10.3109/08958378.2011.647413 [published Online First: 2012/01/31]

48. Dong J, Ma Q. Osteopontin enhances multi-walled carbon nanotube-triggered lung fibrosis by promoting TGF-β1 activation and myofibroblast differentiation. *Particle and fibre toxicology* 2017;14(1):18. doi: 10.1186/s12989-017-0198-0 [published Online First: 2017/06/10]

49. Mossman BT, Churg A. Mechanisms in the pathogenesis of asbestosis and silicosis. *American journal of respiratory and critical care medicine* 1998;157(5 Pt 1):1666-80. doi: 10.1164/ajrccm.157.5.9707141 [published Online First: 1998/05/29]

50. Fujimura N. Pathology and pathophysiology of pneumoconiosis. *Current opinion in pulmonary medicine* 2000;6(2):140-4. doi: 10.1097/00063198-200003000-00010 [published Online First: 2000/03/31]

51. McCunney RJ, Morfeld P, Payne S. What component of coal causes coal workers'

For peer review only - http://bmjopen.bmj.com/site/about/guidelines.xhtml

pneumoconiosis? *Journal of occupational and environmental medicine* 2009;51(4):462-71. doi: 10.1097/JOM.0b013e3181a01ada [published Online First: 2009/04/01]

52. Castranova V, Vallyathan V. Silicosis and coal workers' pneumoconiosis. *Environmental health perspectives* 2000;108 Suppl 4(Suppl 4):675-84. doi: 10.1289/ehp.00108s4675 [published Online First: 2000/08/10]

53. Souza TP, Watte G, Gusso AM, et al. Silicosis prevalence and risk factors in semi-precious stone mining in Brazil. *American journal of industrial medicine* 2017;60(6):529-36. doi: 10.1002/ajim.22719 [published Online First: 2017/05/18]

54. Bevan R, Ashdown L, McGough D, et al. Setting evidence-based occupational exposure limits for manganese. *Neurotoxicology* 2017;58:238-48. doi: 10.1016/j.neuro.2016.08.005 [published Online First: 2016/08/16]

55. Buchanan D, Miller BG, Soutar CA. Quantitative relations between exposure to respirable quartz and risk of silicosis. *Occupational and environmental medicine* 2003;60(3):159-64. doi: 10.1136/oem.60.3.159 [published Online First: 2003/02/25]

56. Krefft S, Wolff J, Rose C. Silicosis: An Update and Guide for Clinicians. *Clinics in chest medicine* 2020;41(4):709-22. doi: 10.1016/j.ccm.2020.08.012 [published Online First: 2020/11/07]

For peer review only

Figure legend:

Fig. 1 Flow diagram of study selection

Fig. 2 The forest plot of pooled prevalence of pneumoconiosis

Fig. 3 The forest plot of sensitivity results for pooling prevalence of pneumoconiosis

Fig. 4 The funnel plot of publication bias of included papers for pooling prevalence of pneumoconiosis

Fig. 5 Forrest plot detailing risk ratios for male workers when comparing participants who developed pneumoconiosis with controls and the subgroup analysis of study quality (NOS<7 versus NOS≥7). "Yes" indicates cases who developed pneumoconiosis; "No" indicates cases who didn't develop pneumoconiosis.

Fig. 6 Forrest plot detailing risk ratios for smoking workers when comparing participants who developed pneumoconiosis with control. "Yes" indicates cases who developed pneumoconiosis; "No" indicates cases who didn't develop pneumoconiosis.

Fig. 7 Forest plot of occupational categories of CWP, tunnelling workers VS others (mining, combining and helping workers). "Yes" indicates cases who developed pneumoconiosis; "No" indicates cases who didn't develop pneumoconiosis.

For peer review only - http://bmjopen.bmj.com/site/about/guidelines.xhtml

Fig. 8 Forest plot of occupational categories of CWP, helping VS others (tunneling, mining and combining workers). “Yes” indicates cases who developed pneumoconiosis; “No” indicates cases who didn’t develop pneumoconiosis.

Fig. 9 Forest plot for summary of relative risk of pneumoconiosis for longest versus shortest duration of dust exposure. ES: effect size.

Fig.10 Dose-response association of duration of dust exposure with risk of pneumoconiosis. Duration of dust exposure was modelled with restricted cubic splines in a multivariate random effects dose-response model. Black line indicates the linear model; sky blue line indicates the spline model; dashed lines represent 95% confidence intervals.

Fig.11 Forest plot for summary of relative risk of pneumoconiosis for each 5-year increase in duration of dust exposure. ES: effect size.

Fig. 12 Forest plot for summary of relative risk of pneumoconiosis for highest versus lowest categories of cumulative dust concentration (CTD). ES: effect size.

Fig. 13 Dose-response association of CTD with risk of pneumoconiosis. CTD was modelled with restricted cubic splines in a multivariate random effects dose-response model. Black line indicates the linear model; sky blue line indicates the spline model; dashed lines represent 95% confidence intervals; CTD: cumulative total dust exposure.

Fig. 14 Forest plot for summary of relative risk of pneumoconiosis for each 50 mg/m^3-years increase in cumulative dust concentration (CTD). ES: effect size.

Fig. 15 Correlation between CSE and incidence of silicosis ($r=0.794$, $P<0.001$). Each gray dot indicates a group of workers exposed each category of CSE, black line

For peer review only - http://bmjopen.bmj.com/site/about/guidelines.xhtml

indicates predicted linear model, and shadow part presents 95% confidence interval of linear model. Y scales are natural logarithmically transformed.

For peer review only

For peer review only - http://bmjopen.bmj.com/site/about/guidelines.xhtml

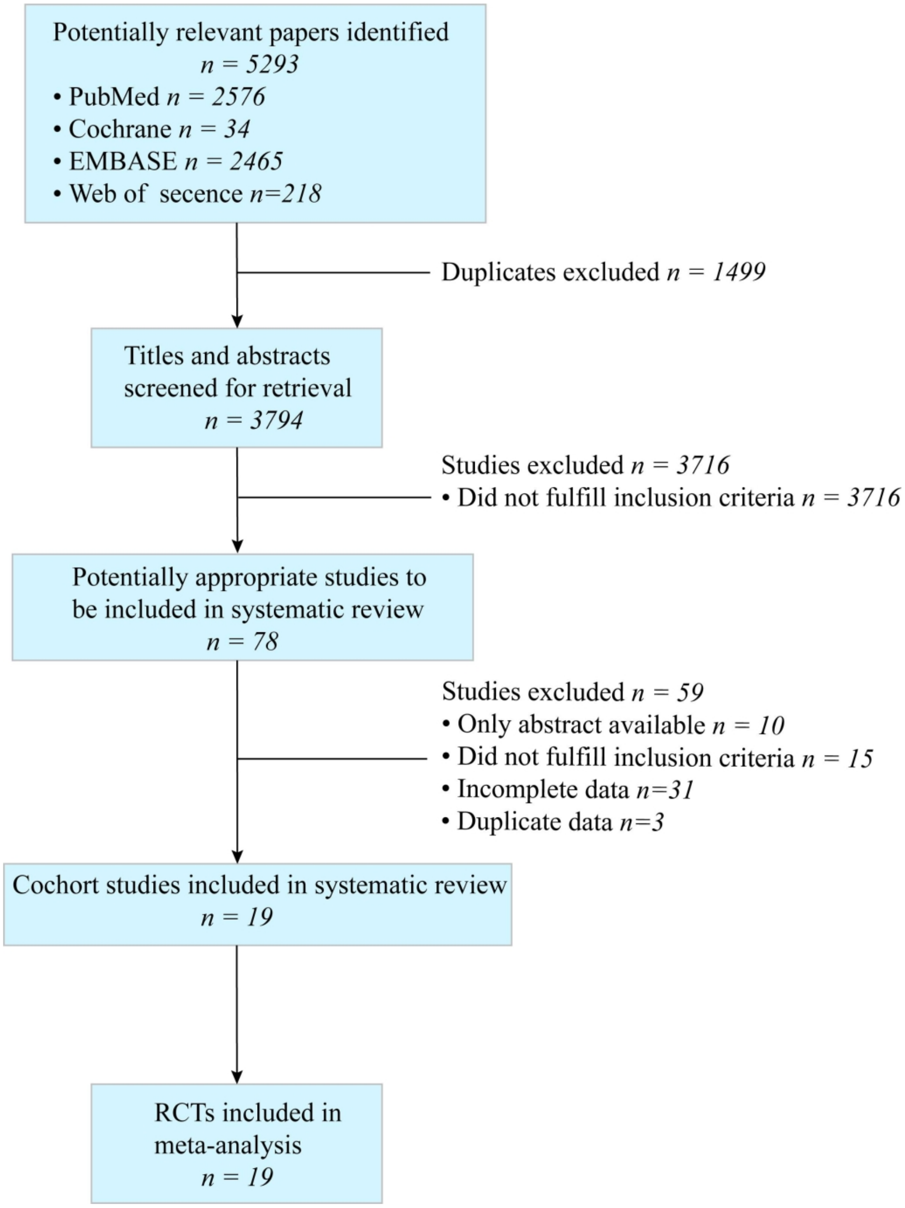

Fig.1 Flow diagram of study selection

633x845mm (96 x 96 DPI)

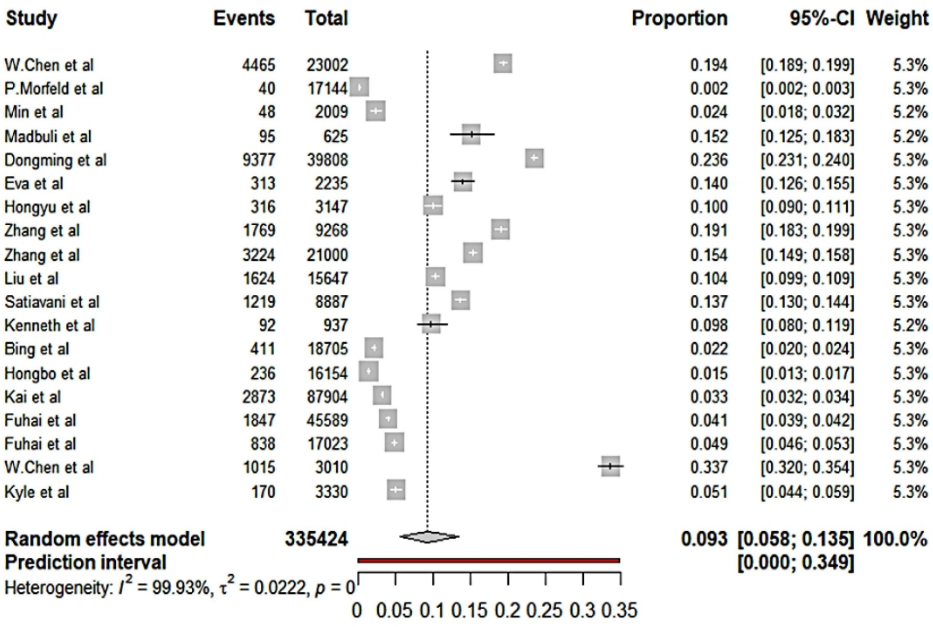

Fig.2 The forest plot of pooled prevalence of pneumoconiosis

849x545mm (96 x 96 DPI)

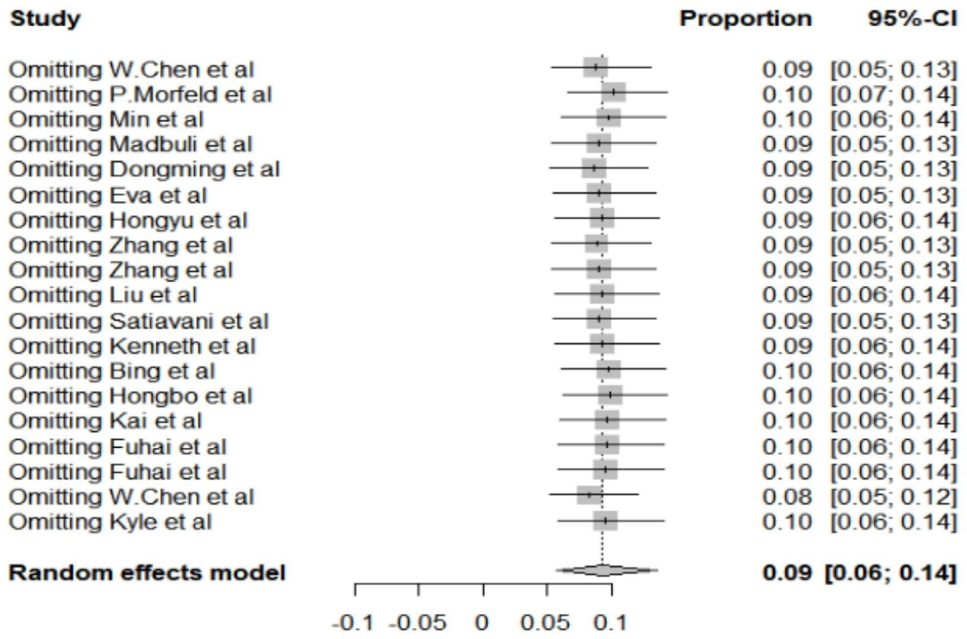

Fig. 3 The forest plot of sensitivity results for pooling prevalence of pneumoconiosis

527x352mm (96 x 96 DPI)

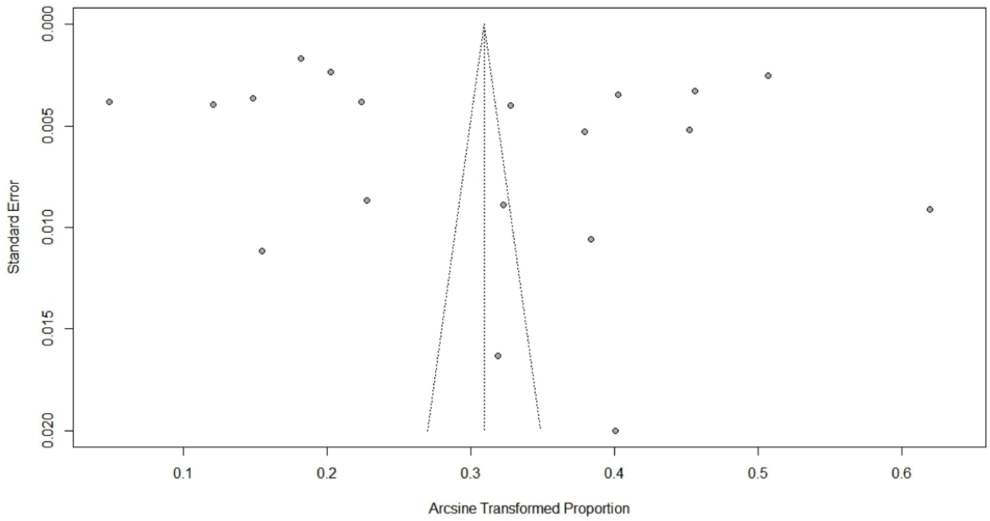

Fig.4 The funnel plot of publication bias of included papers for pooling prevalence of pneumoconiosis

914x489mm (96 x 96 DPI)

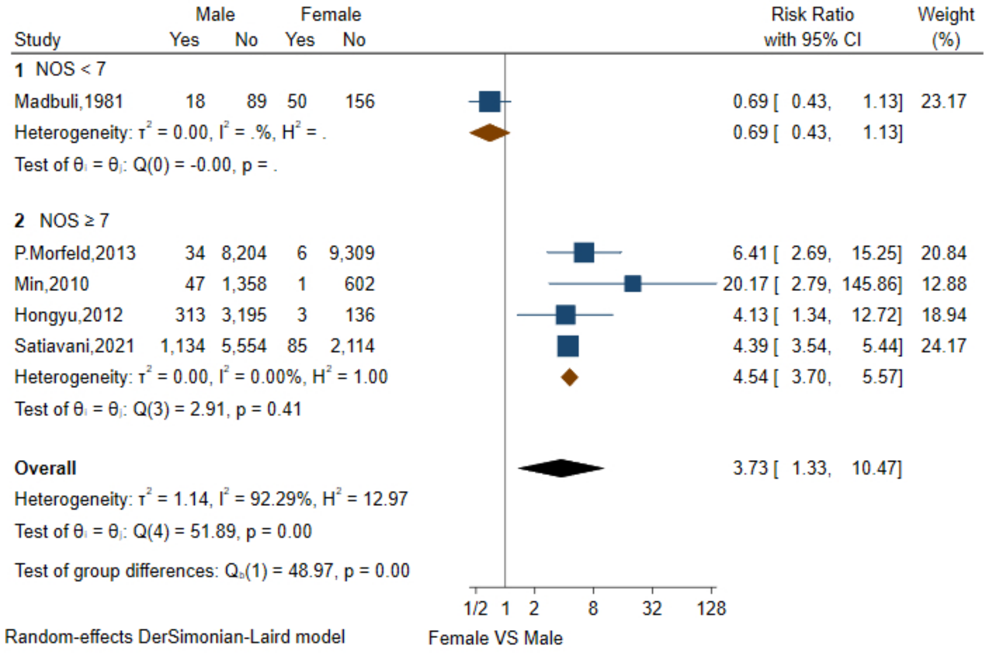

Fig.5 Forrest plot detailing risk ratios for male workers when comparing participants who developed pneumoconiosis with controls and the subgroup analysis of study quality (NOS<7 versus NOS≥7). "Yes" indicates cases who developed pneumoconiosis; "No" indicates cases who didn't develop pneumoconiosis.

251x166mm (72 x 72 DPI)

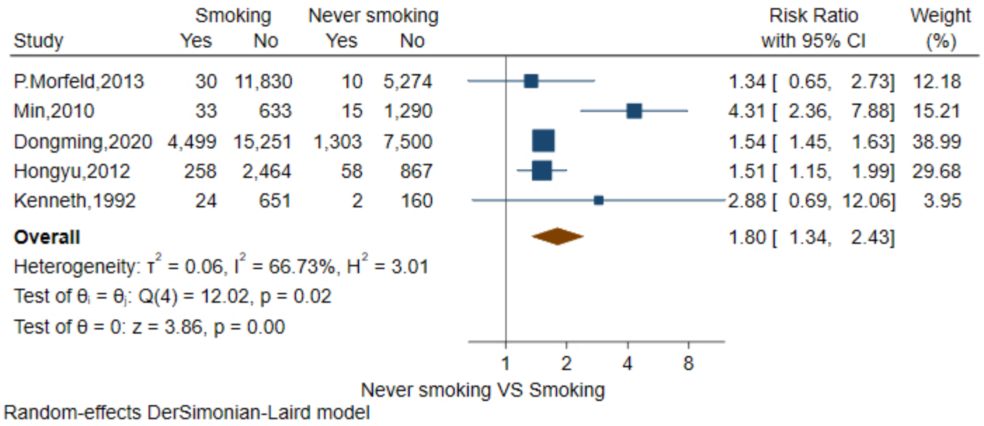

Fig,6 Forrest plot detailing risk ratios for smoking workers when comparing participants who developed pneumoconiosis with controls. "Yes" indicates cases who developed pneumoconiosis; "No" indicates cases who didn't develop pneumoconiosis.

250x110mm (72 x 72 DPI)

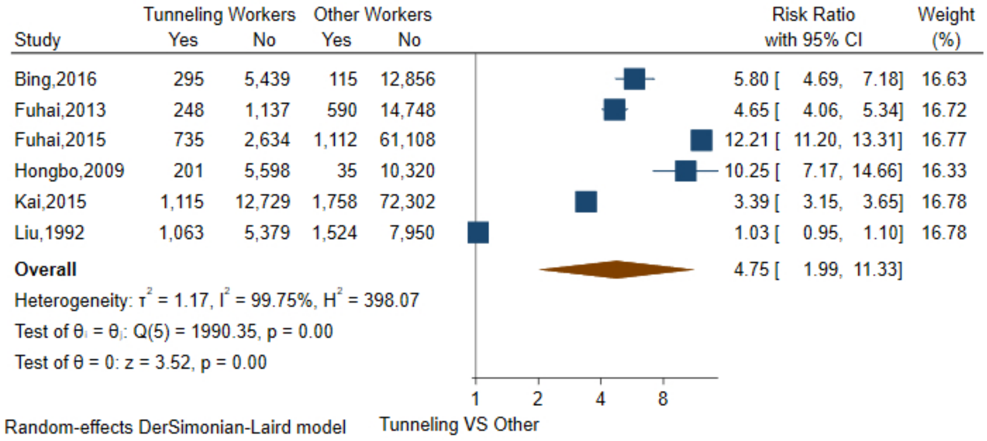

Fig.7 Forest plot of occupational categories of CWP, tunnelling workers VS others (mining, combining and helping workers). "Yes" indicates cases who developed pneumoconiosis; "No" indicates cases who didn't develop pneumoconiosis.

250x113mm (72 x 72 DPI)

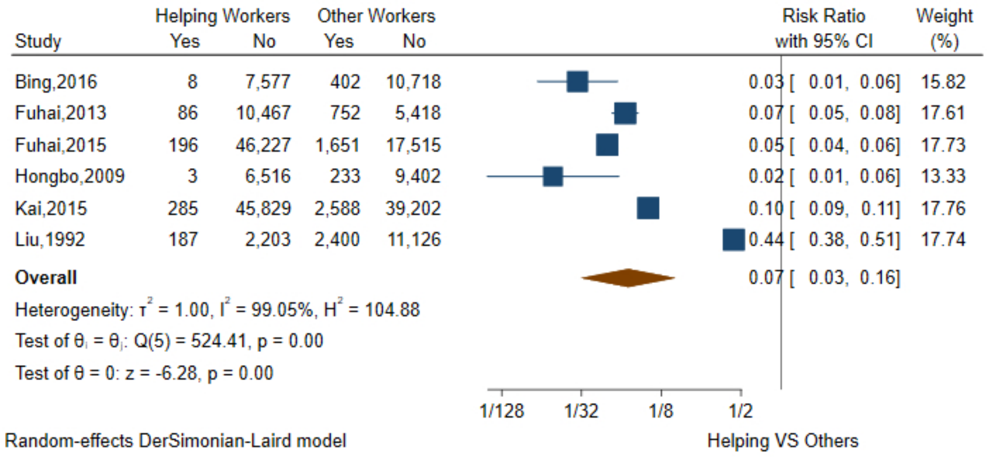

Fig.8 Forest plot of occupational categories of CWP, helping VS others (tunneling, mining and combining workers). “Yes” indicates cases who developed pneumoconiosis; “No” indicates cases who didn’t develop pneumoconiosis.

250x117mm (72 x 72 DPI)

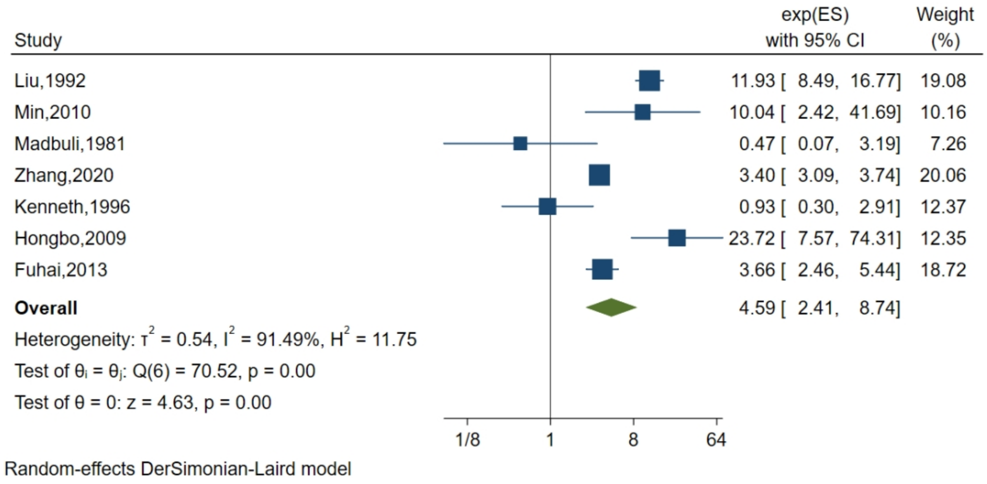

Fig.9 Forest plot for summary of relative risk of pneumoconiosis for longest versus shortest duration of dust exposure. ES: effect size, CI: confidence interval.

435x216mm (72 x 72 DPI)

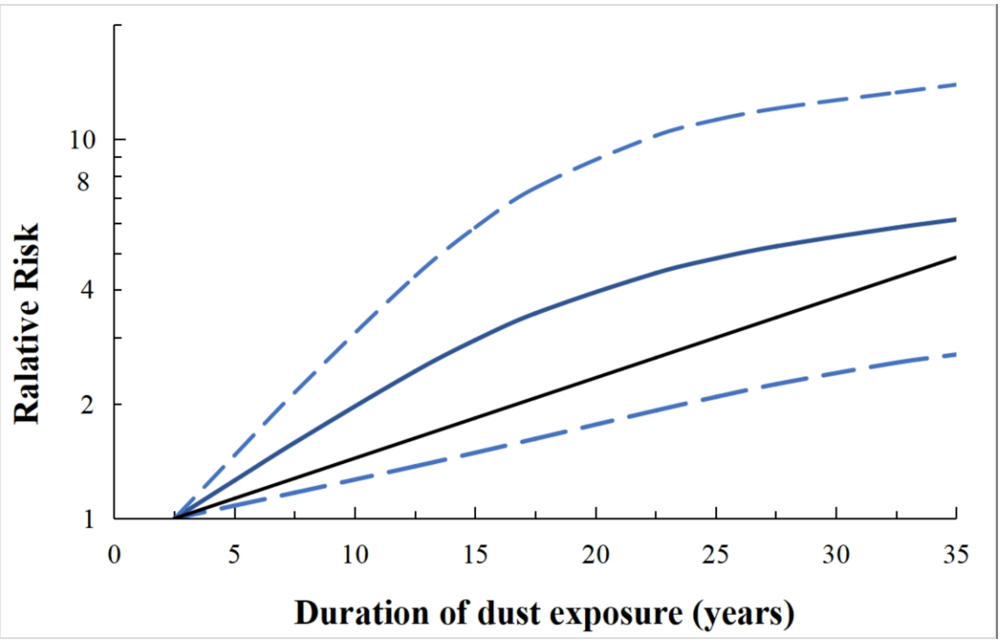

Fig.10 Dose-response association of duration of dust exposure with risk of pneumoconiosis. Duration of dust exposure was modelled with restricted cubic splines in a multivariate random effects dose-response model. Black line indicates the linear model; sky blue line indicates the spline model; dashed lines represent 95% confidence intervals.

201x127mm (150 x 150 DPI)

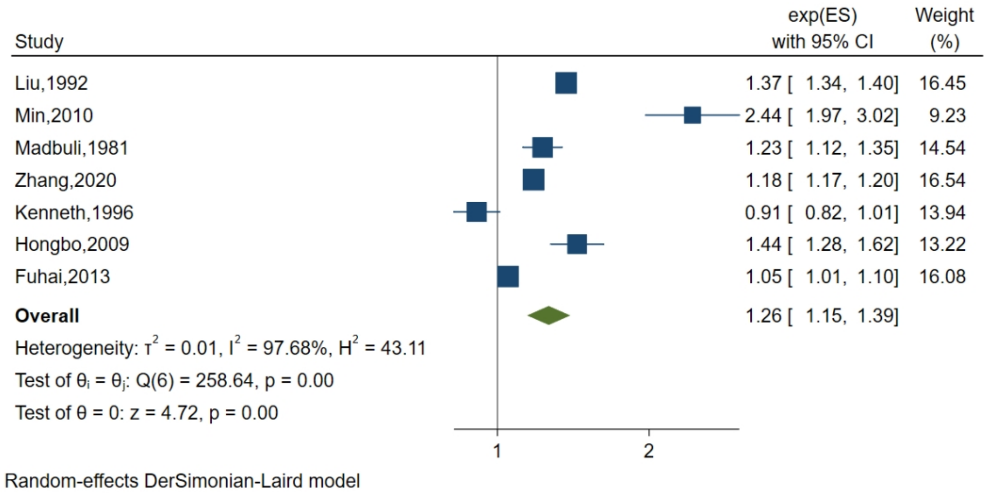

Fig.11 Forest plot for summary of relative risk of pneumoconiosis for each 5-year increase in duration of dust exposure. ES: effect size, CI: confidence interval.

425x216mm (72 x 72 DPI)

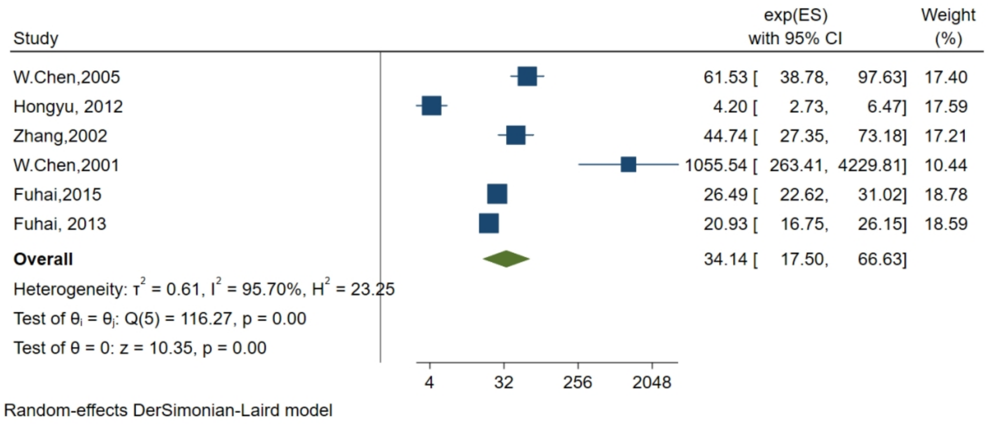

Fig.12 Forest plot for summary of relative risk of pneumoconiosis for highest versus lowest categories of cumulative dust concentration (CTD). ES: effect size, CI: confidence interval.

480x209mm (72 x 72 DPI)

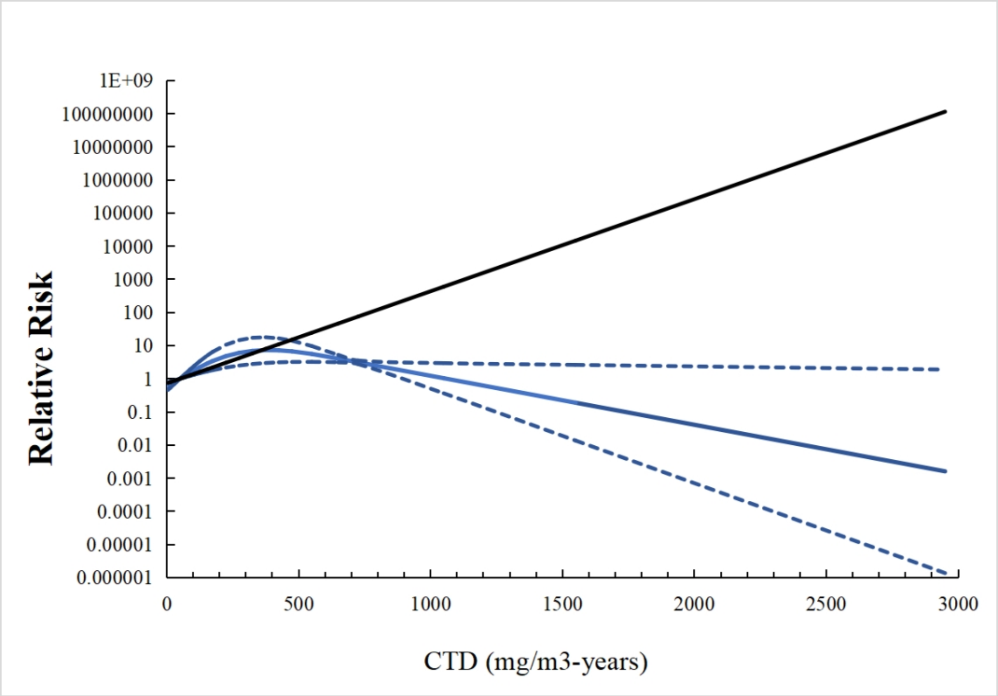

Fig.13 Dose-response association of CTD with risk of pneumoconiosis. CTD was modelled with restricted cubic splines in a multivariate random effects dose-response model. Black line indicates the linear model; sky blue line indicates the spline model; dashed lines represent 95% confidence intervals; CTD: cumulative total dust exposure.

205x143mm (150 x 150 DPI)

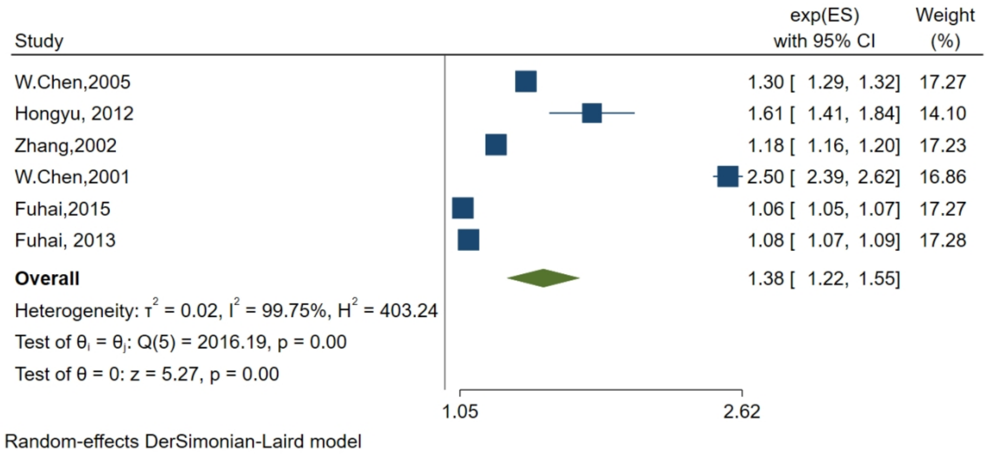

Fig.14 Forest plot for summary of relative risk of pneumoconiosis for each 50 mg/m3-years increase in cumulative dust concentration (CTD). ES: effect size, CI: confidence interval.

460x216mm (72 x 72 DPI)

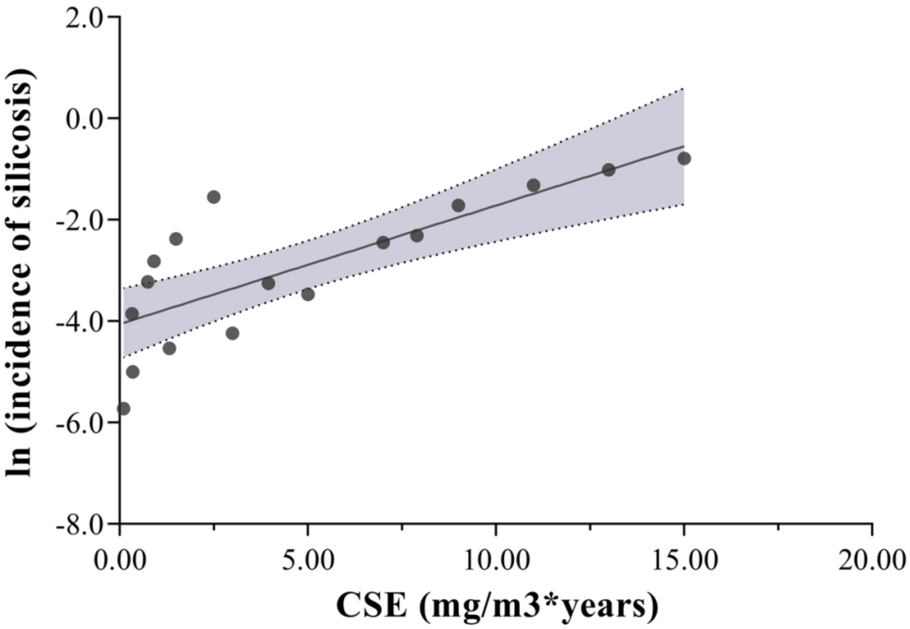

Fig.15 Correlation between CSE and incidence of silicosis (r=0.794, P<0.001). Each gray dot indicates a group of workers exposed each category of CSE, black line indicates predicted linear model, and shadow part presents 95% confidence interval of linear model. Y scales are natural logarithmically transformed. For each category of CSE, we extracted midpoint as the dose in each category. When the highest and lowest categories were open ended, we assumed the length of these open-ended intervals to be the same as those of the adjacent intervals.

115x80mm (300 x 300 DPI)

Search strategy of each online database

Online database	Search number	Search strategy
PubMed	#1	Silicosis [MeSH Terms] OR anthracosis [MeSH Terms] OR anthracosilicosis [MeSH Terms] OR berylliosis [MeSH Terms] OR byssinosis [MeSH Terms] OR siderosis [MeSH Terms] OR Caplan syndrome [MeSH Terms]
	#2	Pneumoconios*[Title/Abstract] OR pneumokoniosis [Title/Abstract] OR asbestos*[Title/Abstract] OR silicosis [Title/Abstract] OR anthracosis [Title/Abstract] OR anthracosilicosis [Title/Abstract] OR berylliosis [Title/Abstract] OR byssinosis [Title/Abstract] OR siderosis [Title/Abstract] OR "Caplan syndrome"[Title/Abstract] OR "Caplan's syndrome" [Title/Abstract] OR aluminosis [Title/Abstract] OR baritosis [Title/Abstract] OR chalicosis [Title/Abstract] OR "occupational lung disease*" [Title/Abstract] OR "dust disease*" [Title/Abstract]
	#3	(Silica[Title/Abstract] OR asbestos[Title/Abstract] OR cotton[Title/Abstract] OR

For peer review only

		bagasse[Title/Abstract] OR iron[Title/Abstract] OR anthracite[Title/Abstract] OR stone[Title/Abstract] OR barium [Title/Abstract] OR aluminum[Title/Abstract] OR beryllium[Title/Abstract] OR "hard metal"[Title/Abstract]) AND (dust*[Title/Abstract] OR particle*[Title/Abstract]) AND (lung[Title/Abstract] OR pleura*[Title/Abstract] OR "respiratory tract"[Title/Abstract] OR pulmonary[Title/Abstract] OR fibrosis[Title/Abstract])
	#4	#1 OR #2 OR #3
	#5	"Cohort studies" [MeSH Terms] OR "longitudinal studies" [MeSH Terms] OR "follow up studies" [MeSH Terms] OR "prospective studies" [MeSH Terms] OR "retrospective studies" [MeSH Terms]
	#6	"Cohort studies" [Title/Abstract] OR "longitudinal studies" [Title/Abstract] OR "follow up studies" [Title/Abstract] OR "prospective studies" [Title/Abstract] OR "retrospective studies" [Title/Abstract]
	#7	#5 OR #6

For peer review only

	#8	#4 AND #7
Cochrane Library	#1	(pneumoconios*):ti,ab,kw OR (pneumokoniosis):ti,ab,kw OR (asbestos*):ti,ab,kw OR (silicosis):ti,ab,kw OR (anthracosis):ti,ab,kw OR (anthracosilicosis):ti,ab,kw OR (berylliosis):ti,ab,kw OR (byssinosis):ti,ab,kw OR (siderosis):ti,ab,kw OR ("caplan syndrome"):ti,ab,kw OR ("caplan's syndrome"):ti,ab,kw OR (aluminosis):ti,ab,kw OR (baritosis):ti,ab,kw OR (chalicosis):ti,ab,kw OR ("occupational lung disease*"):ti,ab,kw OR ("dust disease*"):ti,ab,kw
	#2	MeSH descriptor: [Asbestosis] explode all trees
	#3	MeSH descriptor: [Silicosis] explode all trees
	#4	MeSH descriptor: [Anthracosis] explode all trees
	#5	MeSH descriptor: [Anthracosilicosis] explode all trees
	#6	MeSH descriptor: [Berylliosis] explode all trees
	#7	MeSH descriptor: [Byssinosis] explode all trees
	#8	MeSH descriptor: [Siderosis] explode all trees
	#9	MeSH descriptor: [Caplan Syndrome] explode all trees

For peer review only

	#10	#2 OR #3 OR #4 OR #5 OR #6 OR #7 OR #8
	#11	(silica):ti,ab,kw OR (asbestos):ti,ab,kw OR (cotton):ti,ab,kw OR (bagasse):ti,ab,kw OR (iron):ti,ab,kw OR (anthracite):ti,ab,kw OR (stone):ti,ab,kw OR (barium):ti,ab,kw OR (aluminium):ti,ab,kw OR (beryllium):ti,ab,kw OR ("hard metal"):ti,ab,kw
	#12	(dust*):ti,ab,kw OR (particle*):ti,ab,kw
	#13	(lung):ti,ab,kw OR (pleura*):ti,ab,kw OR ("respiratory tract"):ti,ab,kw OR (pulmonary):ti,ab,kw OR (fibrosis):ti,ab,kw
	#14	#11 AND #12 AND #13
	#15	#1 OR #10 OR #14
	#16	("cohort studies"):ti,ab,kw OR ("longitudinal studies"):ti,ab,kw OR ("follow up studies"):ti,ab,kw OR ("prospective studies"):ti,ab,kw OR ("retrospective studies"):ti,ab,kw
	#17	MeSH descriptor: [Retrospective Studies] explode all trees
	#18	MeSH descriptor: [Prospective Studies] explode all trees

For peer review only

	#19	MeSH descriptor: [Follow-Up Studies] explode all trees
	#20	MeSH descriptor: [Longitudinal Studies] explode all trees
	#21	MeSH descriptor: [Cohort Studies] explode all trees
	#22	#16 OR #17 OR #18 OR #19 OR #21
	#23	#15 AND #22
Web of Science	#1	TS = (Pneumoconios* OR pneumokoniosis OR asbestos* OR silicosis OR anthracosis OR anthracosilicosis OR berylliosis OR byssinosis OR siderosis OR Caplan* syndrome OR aluminosis OR baritosis OR chalicosis OR occupational lung disease* OR dust disease*)
	#2	TS = (Silica OR asbestos OR cotton OR bagasse OR iron OR anthracite OR stone OR barium OR aluminum OR beryllium OR hard metal)
	#3	TS = (dust* OR particle*)
	#4	TS = (lung OR pleura* OR respiratory tract OR pulmonary OR fibrosis)
	#5	#2 AND #3 AND #4
	#6	#1 OR #5
	#7	TS = (cohort studies OR longitudinal studies OR

For peer review only

		follow up studies OR prospective studies OR retrospective studies)
	#8	#6 AND #7
Embase	#1	'siderosis'/exp OR 'occupational lung disease'/exp
	#2	pneumoconios*: ab,ti OR pneumokoniosis: ab,ti OR asbestos*: ab,ti OR silicosis: ab,ti OR anthracosis: ab,ti OR anthracosilicosis: ab,ti OR berylliosis: ab,ti OR byssinosis: ab,ti OR siderosis: ab,ti OR 'caplan* syndrome ': ab,ti OR aluminosis: ab,ti OR baritosis: ab,ti OR chalicosis: ab,ti OR 'occupational lung disease* ': ab,ti OR 'dust disease* ': ab,ti
	#3	silica: ab,ti OR asbestos: ab,ti OR cotton: ab,ti OR bagasse: ab,ti OR iron: ab,ti OR anthracite: ab,ti OR stone: ab,ti OR barium: ab,ti OR aluminum: ab,ti OR beryllium: ab,ti OR 'hard metal ': ab,ti
	#4	dust*: ab,ti OR particle*: ab,ti
	#5	lung: ab,ti OR pleura*: ab,ti OR 'respiratory tract ': ab,ti OR pulmonary: ab,ti OR fibrosis: ab,ti
	#6	#3 AND #4 AND #5
	#7	#1 OR #2 OR #6
	#8	'longitudinal study'/exp OR 'cohort analysis'/exp OR

For peer review only

		'prospective study'/exp OR 'retrospective study'/exp
	#9	'cohort studies': ab,ti OR 'longitudinal studies': ab,ti OR 'follow up studies': ab,ti OR 'prospective studies': ab,ti OR 'retrospective studies': ab,ti
	#10	#8 OR #9
	#11	#7 AND #10

For peer review only

PRISMA 2020 Checklist

Section and Topic	Item #	Checklist item	Location where item is reported
**TITLE**			
Title	1	Identify the report as a systematic review.	p.1-line1~2
**ABSTRACT**			
Abstract	2	See the PRISMA 2020 for Abstracts checklist.	p.2~p.3
**INTRODUCTION**			
Rationale	3	Describe the rationale for the review in the context of existing knowledge.	p.5~p.6-line9
Objectives	4	Provide an explicit statement of the objective(s) or question(s) the review addresses.	p.6-line10~14
**METHODS**			
Eligibility criteria	5	Specify the inclusion and exclusion criteria for the review and how studies were grouped for the syntheses.	p.7-line9~18
Information sources	6	Specify all databases, registers, websites, organisations, reference lists and other sources searched or consulted to identify studies. Specify the date when each source was last searched or consulted.	p.6-line21~22
Search strategy	7	Present the full search strategies for all databases, registers and websites, including any filters and limits used.	p.7-line1~4
Selection process	8	Specify the methods used to decide whether a study met the inclusion criteria of the review, including how many reviewers screened each record and each report retrieved, whether they worked independently, and if applicable, details of automation tools used in the process.	p.7-line6~9
Data collection process	9	Specify the methods used to collect data from reports, including how many reviewers collected data from each report, whether they worked independently, any processes for obtaining or confirming data from study investigators, and if applicable, details of automation tools used in the process.	p.7-line7~8
Data items	10a	List and define all outcomes for which data were sought. Specify whether all results that were compatible with each outcome domain in each study were sought (e.g. for all measures, time points, analyses), and if not, the methods used to decide which results to collect.	p.7-line9~15
	10b	List and define all other variables for which data were sought (e.g. participant and intervention characteristics, funding sources). Describe any assumptions made about any missing or unclear information.	p.7-line9~15
Study risk of bias assessment	11	Specify the methods used to assess risk of bias in the included studies, including details of the tool(s) used, how many reviewers assessed each study and whether they worked independently, and if applicable, details of automation tools used in the process.	p.7-line22~p.8-line5
Effect measures	12	Specify for each outcome the effect measure(s) (e.g. risk ratio, mean difference) used in the synthesis or presentation of results.	p.8-line19~p.9-line6
Synthesis methods	13a	Describe the processes used to decide which studies were eligible for each synthesis (e.g. tabulating the study intervention characteristics and comparing against the planned groups for each synthesis (item #5)).	p.7-line9~18
	13b	Describe any methods required to prepare the data for presentation or synthesis, such as handling of missing summary statistics, or data conversions.	p.7-line7~8
	13c	Describe any methods used to tabulate or visually display results of individual studies and syntheses.	p.8-line17~p.9-line6
	13d	Describe any methods used to synthesize results and provide a rationale for the choice(s). If meta-analysis was performed, describe the model(s), method(s) to identify the presence and extent of statistical heterogeneity, and software package(s) used.	p.9-line7~p.10-line20
	13e	Describe any methods used to explore possible causes of heterogeneity among study results (e.g. subgroup analysis, meta-regression).	p.11-line1~5
	13f	Describe any sensitivity analyses conducted to assess robustness of the synthesized results.	p.11-line6~7
Reporting bias assessment	14	Describe any methods used to assess risk of bias due to missing results in a synthesis (arising from reporting biases).	p.11-line8~9
Certainty	15	Describe any methods used to assess certainty (or confidence) in the body of evidence for an outcome.	p.10-line21~22

For peer review only

PRISMA 2020 Checklist

Section and Topic	Item #	Checklist item	Location where item is reported
assessment			
**RESULTS**			
Study selection	16a	Describe the results of the search and selection process, from the number of records identified in the search to the number of studies included in the review, ideally using a flow diagram.	p.11-line12~19
	16b	Cite studies that might appear to meet the inclusion criteria, but which were excluded, and explain why they were excluded.	p.11-line14~18
Study characteristics	17	Cite each included study and present its characteristics.	p.11-line20~ p.12-line4
Risk of bias in studies	18	Present assessments of risk of bias for each included study.	p.11-line21~22
Results of individual studies	19	For all outcomes, present, for each study: (a) summary statistics for each group (where appropriate) and (b) an effect estimate and its precision (e.g. confidence/credible interval), ideally using structured tables or plots.	p.14-line4~p.15-line12
Results of syntheses	20a	For each synthesis, briefly summarise the characteristics and risk of bias among contributing studies.	p.14-line4~p.19-line5
	20b	Present results of all statistical syntheses conducted. If meta-analysis was done, present for each the summary estimate and its precision (e.g. confidence/credible interval) and measures of statistical heterogeneity. If comparing groups, describe the direction of the effect.	p.14-line4~p.19-line5
	20c	Present results of all investigations of possible causes of heterogeneity among study results.	p.14-line4~p.19-line5
	20d	Present results of all sensitivity analyses conducted to assess the robustness of the synthesized results.	p.14-line9~10
Reporting biases	21	Present assessments of risk of bias due to missing results (arising from reporting biases) for each synthesis assessed.	p.14-line11
Certainty of evidence	22	Present assessments of certainty (or confidence) in the body of evidence for each outcome assessed.	p.12-line5~p.16-line16
**DISCUSSION**			
Discussion	23a	Provide a general interpretation of the results in the context of other evidence.	p.19-line7~p.25-line20
	23b	Discuss any limitations of the evidence included in the review.	p.26-line1~4
	23c	Discuss any limitations of the review processes used.	p.26-line1~4
	23d	Discuss implications of the results for practice, policy, and future research.	p.19-line7~p.25-line20
**OTHER INFORMATION**			
Registration and protocol	24a	Provide registration information for the review, including register name and registration number, or state that the review was not registered.	p.6-line17~18
	24b	Indicate where the review protocol can be accessed, or state that a protocol was not prepared.	p.6-line17~18
	24c	Describe and explain any amendments to information provided at registration or in the protocol.	p.6-line17~18
Support	25	Describe sources of financial or non-financial support for the review, and the role of the funders or sponsors in the review.	p.27-line1~3
Competing interests	26	Declare any competing interests of review authors.	p.27-line5
Availability of data, code and other materials	27	Report which of the following are publicly available and where they can be found: template data collection forms; data extracted from included studies; data used for all analyses; analytic code; any other materials used in the review.	p.27-line10~11

For peer review only

For peer review only - http://bmjopen.bmj.com/site/about/guidelines.xhtml

PRISMA 2020 Checklist

From: Page MJ, McKenzie JE, Bossuyt PM, Boutron I, Hoffmann TC, Mulrow CD, et al. The PRISMA 2020 statement: an updated guideline for reporting systematic reviews. BMJ 2021;372:n71. doi: 10.1136/bmj.n71

For more information, visit: http://www.prisma-statement.org/

For peer review only

BMJ Open

Incidence and influencing factors of occupational pneumoconiosis: A systematic review and meta-analysis.

Journal:	*BMJ Open*
Manuscript ID	bmjopen-2022-065114.R1
Article Type:	Original research
Date Submitted by the Author:	22-Nov-2022
Complete List of Authors:	Su, Xuesen; Shanxi Medical University; The National Health Commission Key Laboratory of Pneumoconiosis (Shanxi, China) Project<br>Kong, Xiaomei; First Hospital of Shanxi Medical University, Department of Pulmonary and Critical Care Medicine; National Health Commission Key Laboratory of Pneumoconiosis (Shanxi China) Project<br>Yu, xiao; First Hospital of Shanxi Medical University, Department of Pulmonary and Critical Care Medicine; The National Health Commission Key Laboratory of Pneumoconiosis (Shanxi, China) Project<br>Zhang, Xinri; First Hospital of Shanxi Medical University, Department of Pulmonary and Critical Care Medicine; National Health Commission Key Laboratory of Pneumoconiosis (Shanxi China) Project
**Primary Subject Heading**:	Public health
Secondary Subject Heading:	Global health, Occupational and environmental medicine, Respiratory medicine
Keywords:	OCCUPATIONAL & INDUSTRIAL MEDICINE, PUBLIC HEALTH, EPIDEMIOLOGY

SCHOLARONE™
Manuscripts

I, the Submitting Author has the right to grant and does grant on behalf of all authors of the Work (as defined in the below author licence), an exclusive licence and/or a non-exclusive licence for contributions from authors who are: i) UK Crown employees; ii) where BMJ has agreed a CC-BY licence shall apply, and/or iii) in accordance with the terms applicable for US Federal Government officers or employees acting as part of their official duties; on a worldwide, perpetual, irrevocable, royalty-free basis to BMJ Publishing Group Ltd ("BMJ") its licensees and where the relevant Journal is co-owned by BMJ to the co-owners of the Journal, to publish the Work in this journal and any other BMJ products and to exploit all rights, as set out in our licence.

The Submitting Author accepts and understands that any supply made under these terms is made by BMJ to the Submitting Author unless you are acting as an employee on behalf of your employer or a postgraduate student of an affiliated institution which is paying any applicable article publishing charge ("APC") for Open Access articles. Where the Submitting Author wishes to make the Work available on an Open Access basis (and intends to pay the relevant APC), the terms of reuse of such Open Access shall be governed by a Creative Commons licence – details of these licences and which Creative Commons licence will apply to this Work are set out in our licence referred to above.

Other than as permitted in any relevant BMJ Author's Self Archiving Policies, I confirm this Work has not been accepted for publication elsewhere, is not being considered for publication elsewhere and does not duplicate material already published. I confirm all authors consent to publication of this Work and authorise the granting of this licence.

For peer review only

Incidence and influencing factors of occupational pneumoconiosis: A systematic review and meta-analysis.

Xuesen Su[1, 3], Xiaomei Kong[2, 3], Xiao Yu[2, 3], Xinri Zhang[2, 3]

Institutional Affiliations:

[1] The First College for Clinical Medicine, Shanxi Medical University, No. 56 Xinjian South Road, Taiyuan, Shanxi, People's Republic of China.

[2] Department of Respiratory and Critical Care Medicine, First Hospital of Shanxi Medical University, Shanxi Medical University, No. 85 Jiefang South Road, Taiyuan, Shanxi, People's Republic of China.

[3] The National Health Commission Key Laboratory of Pneumoconiosis (Shanxi, China) Project, No. 85 Jiefang South Road, Taiyuan, Shanxi, People's Republic of China.

Corresponding author: Xinri Zhang, M.D.

Department of Respiratory and Critical Care Medicine, First Hospital of Shanxi Medical University, Shanxi Medical University, Taiyuan, Shanxi, People's Republic of China.

E-mail address: XinriZhangsxmd@163.com

For peer review only - http://bmjopen.bmj.com/site/about/guidelines.xhtml

Abstract:

Objectives: To determine the incidence of pneumoconiosis worldwide and its influencing factors.

Design: Systematic review and meta-analysis.

Setting: Cohort studies on occupational pneumoconiosis.

Participants: PubMed, Embase, the Cochrane Library, and Web of Science were searched until November 2021. Studies were selected for meta-analysis if they involved at least one variable investigated as an influencing factor for the incidence of pneumoconiosis and reported either the parameters and 95% confidence intervals (CIs) of the risk fit to the data, or sufficient information to allow for the calculation of those values.

Primary outcome measures: The pooled incidence of pneumoconiosis and risk ratio (RR) and 95%CIs of influencing factors.

Results: Our meta-analysis included 19 studies with a total of 335,424 participants, of whom 29,972 developed pneumoconiosis. The pooled incidence of pneumoconiosis was 0.093 (95% CI: 0.085~0.135). We identified the following influencing factors: (1) male (RR=3.74; 95%CI 1.31-10.64; P=0.01), (2) smoking (RR=1.80; 95%CI 1.34-2.43; P=0.0001), (3) tunneling category (RR=4.75; 95%CI 1.96-11.53; P<0.0001), (4) helping category (RR=0.07; 95%CI 0.13-0.16; P<0.0001), (5) age (the highest incidence occurs between the ages of 50 and 60), (6) duration of dust exposure ((RR=4.59, 95% CI 2.41-8.74, P<0.01), (7) cumulative total dust exposure (CTD) (RR=34.14, 95% CI 17.50-66.63, P<0.01). A dose-response analysis revealed a

For peer review only - http://bmjopen.bmj.com/site/about/guidelines.xhtml

significant positive linear dose-response association between the risk of pneumoconiosis and duration of exposure and CTD (P-nonlinearity=0.10, P-nonlinearity=0.16; respectively). The Pearson correlation analysis revealed that silicosis incidence was highly correlated with CSE (r=0.794, P<0.001).

Conclusion: The incidence of pneumoconiosis in occupational workers was 0.093 and seven factors were found to be associated with the incidence, providing some insight into the prevention of pneumoconiosis.

PROSPERO registration number: CRD42022323233.

Keywords: pneumoconiosis, influencing factors, systematic review, meta-analysis

Abbreviations: NOS: Newcastle Ottawa Scale, CWP: Coal Worker's Pneumoconiosis, CTD: cumulative total dust exposure, CSE: cumulative silica exposure.

For peer review only - http://bmjopen.bmj.com/site/about/guidelines.xhtml

Strengths and limitations of this study

- To our knowledge, this is the first systematic review and meta-analysis to estimate the incidence of pneumoconiosis in dust-exposed workers by pooling data from only cohort studies.
- Our study provided quantitative relationship between influencing factors and pneumoconiosis with a larger sample size.
- Due to the limited literature available, some potentially relevant risk factors, such as alcohol consumption and tuberculosis, were excluded from this meta-analysis.
- Significant heterogeneity in the pooled results for a variety of reasons and the small number of included studies may limit the dependability of our results for certain risk factors.

For peer review only

For peer review only - http://bmjopen.bmj.com/site/about/guidelines.xhtml

Introduction

Pneumoconiosis is inclusive of a group of serious pulmonary diseases associated with the inhalation of mineral dust and corresponding reactions of lung tissues, including diffuse fibrosis and progressive lung dysfunction. Although pneumoconiosis among dust-exposed workers has received considerable worldwide attention in terms of incidence, risk factors, early diagnosis, prevention, and intervention, there are currently few established therapeutic options for the disease, highlighting the importance of precautionary measures[1].

Methods of assessing exposure, controlling dust concentration, utilizing tools to prevent exposure, undergoing regular physical examination, and preserving pathological records have been implemented to protect workers from dust inhalation, and eventually, some studies have reported a downward trend since 2015[2-5]—around 527,500 cases of prevalence with about 60,000 new patients reported globally in 2017 corresponds to around 1,111,000 cases of prevalence with about 99,000 new patients in 2016. However, it is still escalating in certain regions, particularly in countries with a large labor force [6 7]. In addition, there are some worse outcomes for a subset of employees. In a 14-year cohort study, Satiavani et al[8] found that the incidence and cumulative risk of silicosis increased more rapidly with increasing years of smoking and dust exposure compared to never-smoking workers, indicating that stricter exposure limits and additional preventative measures may need to be imposed on smoking workers. Therefore, the possible reason for the current situation is that current preventive standards may not be

For peer review only - http://bmjopen.bmj.com/site/about/guidelines.xhtml

able to protect workers with vastly different characteristics, in other words, targeted prevention may be in high demand at this time.

Targeted prevention requires a deeper comprehension of the influencing factors. Even though previous studies have demonstrated that the incidence rate of pneumoconiosis is affected by a variety of factors, including advanced age, male gender, smoking, and dust exposure[8-10], limited prospective data and the homogeneity of studied populations impede their clinical applicability. Furthermore, there is no meta-analysis or systematic review that summarizes the relevant research findings. Consequently, pneumoconiosis risk factors and the shape of their relationship remain largely unknown.

We conducted this systematic review and meta-analysis to provide some clues for the targeted prevention of pneumoconiosis in light of the paucity and controversy of evidence currently available. With a larger sample size, this meta-analysis sought to address and explore the incidence and the impact of different influencing factors on the morbidity of pneumoconiosis, as well as the shape of the relation between them.

Methods

This meta-analysis was completed in accordance with the Preferred Reporting Items for Systematic Reviews and Meta-Analysis (PRISMA) statement[11] and was registered with PROSPERO (Registration NO: CRD42022323233).

Search strategy

By using search terms related to “pneumoconiosis” and “cohort studies”, two reviewers independently and systematically searched through online databases, including PubMed, EMBASE, the Cochrane Library, and Web of Science, before November 2021,

For peer review only - http://bmjopen.bmj.com/site/about/guidelines.xhtml

without regard to the language of publication. Through a thorough discussion with a third reviewer, any discrepancies in the literature search process were resolved. Each electronic database's search terms are detailed in *The supplementary Table*.

Inclusion and exclusion criteria

Two reviewers exported all studies identified by the search strategy to Endnote X9 independently (Thomson Reuters, Philadelphia, USA). The titles and abstracts of all search results were screened first, followed by an independent examination of the full texts of all eligible literature. This meta-analysis required the following inclusion criteria for studies: (1) all involving occupational dust exposure workers (exposed to occupational dust in any period of their work history) are divided into with-pneumoconiosis group and without-pneumoconiosis group according to the national diagnostic criteria of pneumoconiosis published by the International Labor Organization (ILO) (1980, 2000, and 2009 editions) or Ministry of Health of the People's Republic of China (GB 5906-1986, GB 5906-1997, GBZ 70-2002, GBZ 70-2009, and GBZ 70-2015)[12-14]; (2) at least one variable investigated as an influencing factor for the morbidity of pneumoconiosis; (3) the outcome was the number of workers with and without pneumoconiosis under the condition of with and without exposure or with different levels of exposure (4) cohort study designs. The following studies were excluded: (1) studies with insufficient data; (2) the diagnosis of patients with pneumoconiosis was not reported. Disputes regarding the inclusion of eligible papers were resolved through a full discussion with a third reviewer.

Quality assessment

For peer review only - http://bmjopen.bmj.com/site/about/guidelines.xhtml

The quality of the included studies was assessed independently by two authors using the Newcastle Ottawa Scale (NOS). The NOS contains eight categories relating to methodological quality and each study was given an eventual score out of a maximum of 9 points. A score of 0 to 6 indicated a low-quality study, whereas a score of 7 to 9 indicated a high-quality study. Any disagreements about quality assessment were resolved through a full discussion with a third reviewer.

Data extraction

Two reviewers independently conducted data extraction and any disagreements about data extraction were resolved through a full discussion with a third reviewer. The following information was extracted: (1) study characteristics: first author name, publication year, country, duration of follow-up, sample size; (2) type of pneumoconiosis included; (3) participant numbers (separating those who developed pneumoconiosis and those who did not); (4) characteristics of these groups (such as gender); (5) exposure of interest (duration or dust concentrations); (6)information about the outcome of interest (incidence of pneumoconiosis); (7) risk estimates with 95% CIs from any statistic model.

Statistical methods

Statistical analyses were conducted utilizing Statistical Package for the Social Sciences (SPSS, Version 25.0, IBM SPSS Statistics; IBM Corporation, Armonk, NY), Stata 16.1 software (Stata Corp. TX, 2019), and R 4.1.3 statistical software (Schwarzer, 2007; TUNA Team, Tsinghua University, 2022). First, to investigate the incidence of pneumoconiosis in dust-exposed workers, the pooled incidence and its 95% confidence

For peer review only - http://bmjopen.bmj.com/site/about/guidelines.xhtml

intervals (CIs) were calculated using a random-effects model. Second, we chose the influencing factors that were mentioned in at least two studies to investigate the relationship between those and the incidence of pneumoconiosis. A dichotomous variable was reported as frequency and proportion, and the risk ratios (RRs) and their 95%CIs were summarized to assess the outcome using a random-effects model. Third, we compared the highest categories to the lowest categories and conducted dose-response meta-analyses to investigate the association between the risk of pneumoconiosis and dust exposure including two aspects: duration of dust exposure and cumulative total dust exposure concentration (CTD). A P value of less than 0.05 was considered significant for all tests.

First, we examined the association between exposure and pneumoconiosis risk using a random-effects model to summarize RRs and 95% CIs from each study obtained by comparing the highest versus the lowest exposure categories. The dose-response analysis was then conducted using the method described by Greenland[15] and Orsini[16], which required knowledge of the distribution of cases, non-cases, the RRs, and 95%CIs for at least three quantitative exposure categories. For each exposure category, we extracted the midpoint as the dose in each category, estimated by calculating the mean of the lower and upper bound. When the highest and lowest categories were open-ended, we assumed the length of these open-ended intervals to be the same as those of the adjacent intervals.

Using a two-stage, random-effects dose-response meta-analysis, a potential non-linear association between the risk of pneumoconiosis and two aspects of dust exposure was

For peer review only - http://bmjopen.bmj.com/site/about/guidelines.xhtml

investigated. The modeling of dust exposure and restricted cubic splines with three knots at fixed centiles of 10%, 50%, and 90% of the distribution was used to conduct this meta-analysis. We calculated restricted cubic spline models by a generalized least squares trend estimation method, which takes into account the correlation within each set of reported relative risks or hazard ratios, based on the Orsini method. In addition, we pooled study-specific estimates using the restricted maximum likelihood method in a multivariate random-effects meta-analysis. The probability of non-linearity was estimated by testing the null hypothesis under the assumption that the coefficient of the second spline is equal to zero. Using the two-stage generalized least squares trend estimation method, a linear dose-response relationship was examined between the risk of pneumoconiosis and additional 5 years of duration and 50 mg/m^3-years of CTD. First, study-specific slope lines were estimated, and then, using a random-effects model, these lines were combined to obtain an overall average slope. Finally, obtained regression values of the dose-response relationship in the spline and line models at the chosen reference level (the analysis process described above is detailed in the *supplementary file*). Statistical analyses were conducted using Stata 16.1 software.

The association between cumulative silica exposure (CSE) and the incidence of silicosis was estimated using the Pearson correlation. Due to the incidence rate of silicosis being non-normally distributed data (the higher the incidence, the smaller number of groups), the natural logarithm of the incidence rate was used as the normal distribution variable. Both the Q statistic and I^2 were calculated as indicators of heterogeneity (significant heterogeneity defined as $I^2>50\%$, $P<0.05$). Subgroup analyses were performed to

For peer review only - http://bmjopen.bmj.com/site/about/guidelines.xhtml

investigate possible reasons for the significant heterogeneity and address the effects of important factors. We divided the data into subgroups based on the type of pneumoconiosis (CWP versus other types of pneumoconiosis), occupational category for CWP, and study quality (NOS<7 versus NOS≥7), and then compared across subgroups.

Eliminating individual studies one by one, a sensitivity analysis was conducted to determine the effect of each study on the pooled estimate. Using the funnel plots and Egger's tests, we evaluated the potential risk of publication bias in the included studies.

Patient and public involvement

Patients and/or the public were not involved in the design, conduct, reporting, or dissemination plans of this research.

Results

Study selection and study characteristics

Fig. 1 presents a detailed flowchart of the literature screening. In the initial search, 5293 articles were found. After excluding papers that were duplicates or did not meet the inclusion criteria, 78 full-text articles of potentially relevant studies were identified. Following a full-text review, an additional 59 articles were excluded: forty-four studies with insufficient and duplicate research data, six cross-sectional studies, five reviews, three case-control studies, and one case report. Finally,19 studies were included in our meta-analysis.

Among these studies, there were six prospective studies, nine retrospective studies, and four retrospective-prospective cohort studies. The range of NOS scores was 5 to 9, and

For peer review only - http://bmjopen.bmj.com/site/about/guidelines.xhtml

publish years ranged from 1981 to 2021. This meta-analysis involved 335,424 participants, including 29,972 pneumoconiosis patients and 305,452 non-pneumoconiosis workers. We analyzed seven factors influencing the morbidity of pneumoconiosis, including gender, smoking, coal workers' occupational categories, duration of dust exposure, age, CTD, and CSE (*Table 1*).

Table 1 Study characteristics

Study	Country	Design	Pneumoconiosis reported	Duration (years)	Sample size (pneumoconiosis cases/total cases)	Risk factors reported	NOS scores
W. Chen et ai[9]	China	Retrospective	Silicosis	45	4465/23002	F5	7
P. Morfeld et al[17]	Germany	Prospective	Silicosis	20	40/17144	F1, F2	8
Min et al[18]	China	Prospective	Silicosis	29	48/2009	F1, F2, F4, F7	9
Madbuli et al[19]	Egypt	Prospective	Byssinosis	10	95/625	F1, F4	5
Dongming et al[20]	China	Retrospective and Prospective	Silicosis	43	9377/39808	F2, F6	9

For peer review only - http://bmjopen.bmj.com/site/about/guidelines.xhtml

Eva et al[21]	South African	Prospective	Silicosis	23	313/2235	F6	7
Hongyu et al[22]	China	Retrospective and Prospective	Silicosis	44	316/3147	F1, F2, F5, F7	9
Zhang et al[23]	China	Retrospective	Silicosis	35	1769/9268	F5	6
Zhang et al[24]	China	Retrospective	CWP		3224/21000	F4	7
Liu et al[25]	China	Retrospective	CWP	7	1624/15647	F3, F4	5
Satiavan et al[8]	China	Prospective	Silicosis	54	1219/8887	F1	7
Kenneth et al[26]	U.S.	Prospective	Silicosis	5.5	92/937	F2, F4, F6	7
Bing et al[10]	China	Retrospective	CWP	48	411/18705	F3	8
Hongbo et al[27]	China	Retrospective	CWP	50	236/16154	F3, F4	8
Kai et al[6]	China	Retrospective and Prospective	CWP	44	2873/87904	F3	7
Fuhai et	China	Retrospective	CWP	41	1847/45589	F3, F5	8

For peer review only - http://bmjopen.bmj.com/site/about/guidelines.xhtml

For peer review only

al[28]		and Prospective					
Fuhai et al[29]	China	Retrospective	CWP	42	838/17023	F3, F4, F5	9
Kyle et al[30]	U.S.	Retrospective	Silicosis	25	170/3330	F6	7
W. Chen et al[31]	China	Retrospective	Silicosis	35	1015/3010	F5	6

Risk factors: F1: gender; F2: smoking; F3: coal workers' occupational categories; F4: duration of dust exposure; F5: cumulative total dust exposure (CTD); F6: cumulative silica exposure (CSE); F7: age. CWP: Coal Worker's Pneumoconiosis

Pooled results, sensitive analysis, publication bias of the incidence of pneumoconiosis

Based on the results of the random-effects method, the pooled incidence of pneumoconiosis among dust-exposed workers was 0.093 (95%CI: 0.085-0.135), with a high-level heterogeneity between studies (I^2 = 99.93 %, P < 0.0001) (*Fig.2, Table 2*). The sensitive analysis revealed that no individual studies significantly affected the pooled results (*Supplementary Fig. 1*). Egger's linear regression test (t = 0.81, p = 0.427) indicated that there was no significant publication bias, and the funnel plot was presented in *Supplementary Fig. 2*.

Table 2 The pooled results and subgroup analysis of the incidence of pneumoconiosis

	Number	n/N	Pooled incidence rate	Heterogeneity

For peer review only

		of studies		Effects size	Lower limit	Upper limit	I^2	p
Total		19	29,972/335,424	0.093	0.058	0.135	99.93%	<0.001
Pneumoconiosis	silicosis	11	18,824/112,777	0.119	0.064	0.188	99.92%	<0.001
	CWP	7	11,503/222,022	0.044	0.024	0.082	99.90%	<0.001
Gender	male	5	3,112/19,946	0.034	0.000	0.123	98.96%	<0.010
	female	5	972/12,462	0.012	0.002	0.086	98.45%	<0.010
Smoker	yes	5	4,845/35,677	0.064	0.012	0.152	99.93%	<0.001
	no	5	1,388/16,479	0.019	0.005	0.076	98.88%	<0.010
Occupational categories of coal workers	tunneling	6	3,675/36,573	0.112	0.062	0.174	99.63%	<0.010
	mining	6	1,508/21,948	0.059	0.025	0.093	99.06%	<0.010
	combining	6	2,861/42,886	0.049	0.020	0.123	99.82%	<0.001
	helping	6	765/119,854	0.005	0.001	0.019	99.56%	<0.010

Subgroup analysis of the incidence of pneumoconiosis and comparison results

Table 2 demonstrates the pooled incidence of all subgroups stratified by different types of pneumoconiosis, gender, smoking status, and occupational categories of coal workers. The pooled incidence of silicosis (0.119, 95%CI: 0.064-0.188) was higher than that of CWP (0.044, 95%CI: 0.024-0.082). Males had a higher pooled incidence (0.034, 95%CI: 0.000-0.123) than females (0.012, 95%CI: 0.002-0.086). Additionally, the pooled incidence among smokers (0.064, 95 %CI: 0.012 -0.152) was higher than that among nonsmokers (0.019, 95%CI: 0.005-0.076). The pooled incidence of CWP

For peer review only

For peer review only - http://bmjopen.bmj.com/site/about/guidelines.xhtml

in various occupational categories varied. Tunneling workers had an incidence of 0.112 (95%CI: 0.062-0.174), mining workers was 0.059 (95%CI: 0.025-0.093), combining workers was 0.049 (95%CI: 0.020-0.123) and helping workers was 0.005 (95%CI: 0.001-0.019).

Influencing factors of pneumoconiosis

We found seven factors influencing the incidence of pneumoconiosis, including gender, smoking, age, coal workers' occupational categories, duration of dust exposure, CTD, and CSE.

Gender

The association between gender and the incidence of pneumoconiosis was examined in five studies, four of which were of high quality and one of which was of low quality. Using a random-effects model, the pooled results of five studies indicated that male workers had a significantly higher risk than female (RR =3.74; 95%CI 1.31-10.64; P=0.01) with heterogeneity (P<0.00001, I^2=93%). Subgroup analysis for only high-quality studies (RR =4.54, 95%CI 3.70-5.57, P<0.00001) with non-significant heterogeneity (P=0.40, I^2=0%) revealed the possible cause of significant heterogeneity (*Fig. 3a*).

Smoking

Five studies, all of which were high-quality studies, reported the comparison between smoking and never-smoking. The pooled results of five studies suggested that smoking workers had an obviously higher risk than non-smoking workers (RR =1.80; 95%CI

For peer review only - http://bmjopen.bmj.com/site/about/guidelines.xhtml

1.34-2.43; P=0.0001) with heterogeneity (P=0.02, I^2=67%) by using a random-effects model. No data pooling was possible for any identified subgroup (*Fig. 3b*).

Age

Two studies reported the association between age and morbidity of pneumoconiosis. These studies[18 22] roughly suggest that the highest incidence occurs between the ages of 50 and 60. When the age is below 50 years old, the incidence rate increases with age, whereas when the age is above 60 years old, the incidence rate decreases with age.

Occupational category

Since occupational categories were only reported in CWP-related studies, we summarized the relationship between occupational categories and the incidence rate of pneumoconiosis in these studies. In six studies involving 220,911 participants and 8,791 patients, a comparison was conducted between four distinct occupational categories. The incidence was highest among tunneling workers (0.112, 95%CI 0.062-0.174) and lowest among helping workers (0.005, 95%CI 0.001-0.019), according to the pooled results from various occupational categories. Thus, we compared tunneling workers to other categories (mining, combining, and helping workers) and helping workers to workers in other categories (tunneling, mining, and combining workers). Using a random-effects model, the pooled results of six studies indicated that tunneling workers had a significantly higher risk than other workers (RR=4.75; 95%CI 1.96-11.33; P<0.001) with heterogeneity (P<0.001, I^2=100%) (*Fig. 3c*). And helping workers had an obviously lower risk than other workers (RR=0.07; 95% CI 0.03-0.16;

For peer review only - http://bmjopen.bmj.com/site/about/guidelines.xhtml

P<0.001) with heterogeneity (P<0.001, I^2=99%) (*Fig. 3d*). Significant heterogeneity and the limited number of included studies may compromise the validity of our findings.

Duration of dust exposure

Seven studies, including six high-quality studies and one low-quality study, reported the relationship between the duration of dust exposure and the incidence of pneumoconiosis. The summary effect size for pneumoconiosis morbidity comparing the longest and shortest duration of dust exposure was calculated by using a random-effects model (RR=4.59, 95%CI 2.41-8.74, P<0.01; *Supplementary Fig. 3*), indicating a significant association; however, evidence of high heterogeneity was found between studies (I^2=91.49%, P<0.01). In dose-response analysis, there was no indication of a nonlinear association between the duration of dust exposure and risk of pneumoconiosis (P-nonlinearity=0.10; *Fig. 4a*). Furthermore, linear dose-response meta-analysis showed that an increase in duration by 5 years was positively associated with a 26% higher risk of pneumoconiosis (RR=1.26, 95%CI 1.15-1.39, P<0.01; *Fig. 4c*) with heterogeneity (I^2=97.68%, P<0.01), regarding 2.5 years as a reference. No data pooling was possible for dose-response meta-analysis of any identified subgroup.

Cumulative total dust concentration (CTD)

Six studies involving a total of 189,037 participants and 10,271 patients reported the association between CTD and pneumoconiosis incidence. The summary effect size for pneumoconiosis morbidity comparing the highest and lowest CTD was calculated by using a random-effects model (RR=34.14, 95%CI 17.50-66.63, P<0.01; *Supplementary Fig. 4*), indicating a significant association; however, there was evidence of high

For peer review only - http://bmjopen.bmj.com/site/about/guidelines.xhtml

heterogeneity was found among studies (I^2=95.70%, P<0.01). After pooling six study-specific estimates using the restricted maximum likelihood method in a random-effects model, the result of the formal statistical hypothesis test indicated that there was no indication of a nonlinear association between the duration of dust exposure and pneumoconiosis risk (P-nonlinearity=0.16; *Fig. 4b*). Furthermore, linear dose-response meta-analysis showed that a 50 mg/m^3-years increase in CTD was associated with a 38% higher risk of pneumoconiosis (RR:1.38, 95%CI 1.22-1.55, P<0.01; *Fig. 4d*) with heterogeneity (I^2=99.75%, P<0.01), regarding 50 mg/m^3-years as a reference.

Different sorts of risk values retrieved from each study were determined to be included in the analysis during the data analysis. Only one of the six studies had an adjusted risk ratio. Therefore, this study was eliminated for sensitivity analysis. Sensitivity analysis for the link between CTD and the risk of pneumoconiosis revealed that excluding the study by *Hongyu et al*[22] did not significantly modify the estimates, either in the shape of association (P-nonlinearity=0.74; *Supplementary Fig. 5*) or the pooled effect sizes (detailed in *Supplementary Fig. 6* and *Supplementary Fig. 7*)

Cumulative Silica Exposure (CSE)

Four studies reported the association between CSE and the incidence of silicosis, which involved 40,212 participants and 6,371 patients. The Pearson correlation analysis revealed that the incidence of silicosis and CSE were highly correlated (r=0.794, P<0.001, *Fig. 5*). No data pooling was possible for regression analysis of any identified subgroup.

Discussion

For peer review only - http://bmjopen.bmj.com/site/about/guidelines.xhtml

This is, to our knowledge, the first systematic review and meta-analysis to estimate the incidence of pneumoconiosis in dust-exposed workers and the relationship between pneumoconiosis and various influencing factors. Our meta-analysis revealed that the incidence among workers exposed to dust was approximately 9.3% (95% CI: 8.5%~13.5%). And the effect sizes of influencing factors, including gender, smoking, age, occupational categories, duration of dust exposure, CTD, and CSE, was investigated using a larger sample size and a more comprehensive methodology than ever before. Further, in terms of the quantified relationship between risk factors and the incidence, we found a linear dose-response relationship between CTD and exposure duration and the pneumoconiosis risk, and a linear correlation between CSE and the incidence of silicosis.

The pooled incidence estimated by this meta-analysis was higher than the Global Burden of Disease Study 2017 analysis's reported incidence rate of 7.5% (95%CI 6.6%-8.4%)[32]. The first possible reason for it was that the majority of studies included in our meta-analysis originated from nations with relatively higher incidence rates of pneumoconiosis, such as China and America. Second, the incidence of pneumoconiosis has been declining recently as preventive measures have improved. It is also possible that the pooled incidence is higher than the incidence or prevalence reported by recent studies because the type of article included in this meta-analysis is a cohort study. This is based on the observation that our pooled CWP incidence differs significantly from that reported by the analysis from the Global Burden of Disease Study 2017 (1.9%, 95%CI 1.5%-2.5%)[32], but is comparable to that (3.7%, 95% CI

For peer review only - http://bmjopen.bmj.com/site/about/guidelines.xhtml

3.0~4.5%) of a meta-analysis for CWP among underground miners[33]. In comparison to recent studies, the pooled results of only cohort studies typically allowed for larger sample size and greater access to the true conclusion.

In addition, our results demonstrated that the pooled incidence of silicosis is higher than that of CWP, which is consistent with the findings of numerous previous studies. Numerous pathological processes, including macrophage autophagy, autoimmune, apoptosis, and proptosis, have indicated that crystalline silica or quartz, the most prevalent occupational dust, is the most significant cause of silicosis[34-36]. However, rather than silica or quartz, the amount of carbon in the coal is what largely determines the risk of CWP[37-39]. In other words, the incidence of silicosis is higher than that of CWP, possibly because the pathogenic exposures that cause silicosis are more common than those that cause CWP. Nevertheless, the mechanism that perfectly explains the epidemiological data remains elusive.

Consistent with many previous studies[8 18 40], our findings showed a difference in the pooled incidence of pneumoconiosis between men and women, with men being at higher risk than women. On the other hand, some previous studies have shown no difference in the incidence of pneumoconiosis between men and women[41-43], however, limitations such as cross-sectional studies, medical examination, and a short period of follow-up rendered their findings inconclusive. There are several possible explanations for the difference in pneumoconiosis risk between men and women. First, the greater physical strength of men during labor contributes to more rapid pulmonary ventilation, which in turn results in the inhalation of a significantly higher concentration of

For peer review only - http://bmjopen.bmj.com/site/about/guidelines.xhtml

occupational dust over the same period when compared to women[44 45]. Second, as demonstrated by previous gender-related research, some gender disparities are largely mediated by differences in the expression of genotypes, such as differential expression of pro-inflammatory cytokines[46 47]. Third, a higher proportion of male smokers compared to female smokers was associated with an increased risk of pneumoconiosis in the male group.

Concerning the relationship between smoking and pneumoconiosis, the current studies primarily focus on the morbidity and mortality of pneumoconiosis[20 48], and it is generally accepted that smoking can promote the occurrence of pneumoconiosis and also increase all-cause mortality in patients with pneumoconiosis, despite the fact that the risk for smoking varies widely between studies. In addition, Dongming *et al*[20] found that as the dust concentration rises, the incidence of pneumoconiosis increases more rapidly in smokers than in nonsmokers. Previous animal research demonstrated that exposure to tobacco smoke alone had a minimal effect on these toxicity parameters, however, the combined exposure to tobacco smoke and occupational dust worsened the lung response in comparison to exposure to either agent alone[49]. One possible explanation is that cigarette smoking destroys the ciliated epithelium in the bronchi and reduces the clearance of dust from the lungs. Moreover, Dorman *et al*[50]. discovered that exposure to tobacco smoke exacerbated the silica dust-induced lung toxicity in rats by altering the level of secretion of several cytokines/chemokines and oxidants produced by the bronchoalveolar phagocytes. Alternately, exposure to a toxic agent may result in changes in gene expression accompanied by alterations in the functions mediated by

For peer review only - http://bmjopen.bmj.com/site/about/guidelines.xhtml

the affected genes, resulting in toxicity to the target organ. SPP1, a gene involved in lung inflammatory and fibrotic responses to toxic particles, was overexpressed by 2.85-, 36.69-, and 72.61-fold, in the smoke, silica, and smoke plus silica exposed rats, respectively, compared to the air controls[51]. Currently, it is imperative that smokers quit, and they may require lower dust concentrations and stricter prevention and control of dust inhalation than nonsmokers. However, the effects of tobacco smoke and occupational dust on the lungs are complex, and further study is required to determine how they interact.

There are occupational categories for any worker who is exposed to dust on the job, but the vast majority of occupational studies on this topic have focused on coal miners[6 27 28]. The average dust concentration in the workplace is one of the possible primary causes of disparate morbidity. Kai *et al*[6] found that the incidence of pneumoconiosis in the same occupational category in various coal mines was proportional to the average dust concentration; that is, the incidence increased as the average dust concentration increased. There are, of course, numerous other distinctions between occupational categories, such as working hours, work habits, and lifestyles. Whether these factors are also the reasons for the different incidence rates between them needs to be discovered and confirmed by future research.

The observed positive association is consistent with results from previous clinical studies on the duration and incidence of pneumoconiosis. In 11 state-owned coal mines, for instance, a retrospective cohort study found that the rate of morbidity increased sharply with the length of employment[24]. Since the majority of studies on the risk of

For peer review only - http://bmjopen.bmj.com/site/about/guidelines.xhtml

different lengths of employment have been measured in increments of five years, our study also calculated the risk increase for each additional five years of employment. Some reviews[52-55] proposed that the development and progression of pneumoconiosis are chronic inflammatories, anti-inflammatory, and fibrotic processes, indicating that pneumoconiosis progresses gradually under the continuous stimulation of occupational dust. So, we believe that reducing the duration of employment in an environment with a high dust concentration or implementing targeted shift changes for workers with an overall increase in morbidity. However, the efficacy and mechanism of these targeted preventive measures must be confirmed through additional research.

Although there is no comparable meta-analysis for CTD, our results are consistent with previous clinical research that revealed a positive association between the incidence of pneumoconiosis and CTD[6 9 22 29]. Present studies suggested that dust is still the primary occupational hazard for developing pneumoconiosis; therefore, if dust concentrations increase, pneumoconiosis risk should theoretically increase as well[29]. We believe that CTD is a more significant method for evaluating exposure than either the average dust concentration or the duration of exposure because it takes both into account. However, we detected further risk growth beyond the increment of 50 mg/m^3-years in this meta-analysis, due to a reduction in sample size, the relationship between a higher concentration of CTD and the risk of pneumoconiosis is less reliable. The association between CTD and the incidence of pneumoconiosis should be further investigated and analyzed, and the Occupational Safety and Health Administration (OSHA) should eventually impose permissible limits for CTD.

For peer review only - http://bmjopen.bmj.com/site/about/guidelines.xhtml

In the absence of the effect size of silica on the risk of silicosis, we estimated the association between CSE and the incidence of silicosis using Pearson correlation analysis. Previous studies have identified a positive correlation between the incidence of silicosis and silica[20 21 30]. For example, Tamires *et al* found that the incidence ratio of silicosis increased by 4% for each additional year of silica exposure[56]. However, CSE is a more accurate method of assessing silica exposure than duration because it takes dust concentration into account. There is no previous study to explore the linear association between CSE and the incidence of silicosis because of fewer exposures' stratification in each study. In our meta-analysis, we found a positively strong linear correlation. At present, most countries regulate exposure limits for respirable crystalline silica (RCS) in the range of 0.05 mg/m^3 to 0.1 mg/m^3 [57]. However, this occupational exposure standard is too strict for most workplaces, and they may exceed it for brief periods[58]. Moreover, new information suggests that it would be more effective to control the risk of pneumoconiosis by imposing a more accurate assessment of dust exposure, as measured in hours, days, weeks, or months[59]. We believed that our findings could provide some insights into this new understanding.

Limitations

There are some inevitable limitations in our meta-analysis. First, significant heterogeneity in the pooled results for a variety of reasons and the small number of included studies may reduce the validity of our results for certain risk factors. Second, given the limited literature available, some potentially relevant risk factors, such as alcohol consumption and tuberculosis, were not included in this meta-analysis.

For peer review only - http://bmjopen.bmj.com/site/about/guidelines.xhtml

Conclusions

In conclusion, this meta-analysis revealed that the incidence of pneumoconiosis among occupational workers was 0.093 and that there were seven factors associated with this incidence, including male, smoking, age, coal workers' occupational categories, duration of dust exposure, CTD, and CSE. In addition, we discovered a linear dose-response relationship between the risk of pneumoconiosis and the duration of dust exposure and CTD. Our findings will provide hints for pneumoconiosis prevention strategies.

Author Contributions

Xuesen Su, Xiao Yu, and Xiaomei Kong completed the search, determined eligible papers for inclusion, and completed the quality assessment, and data extraction. Xuesen Su and Xinri Zhang completed the meta-analysis. All authors contributed to the writing of the final manuscript.

Acknowledgments

The authors would also like to thank Professors from the College of Public Health, Shanxi Medical University for the advice on the study design and revise opinion.

Funding

Organization: 1. The National Health Commission Key Laboratory of Pneumoconiosis (Shanxi, China) Project; Project number: No. 2020-PT320-005; Project Leader: Xinri Zhang. 2. Key Laboratory of Respiratory Disease Prevention and Control of Shanxi.

Conflict of Interest

For peer review only - http://bmjopen.bmj.com/site/about/guidelines.xhtml

The authors have no conflict of interest to declare.

Data availability statement

Extra data can be accessed via the Dryad data repository at http://datadryad.org/ with the doi: 10.5061/dryad.rv15dv4c3

Ethics Approval

No ethical approval is necessary for this systematic review. And all the studies included in this meta-analysis have obtained ethical approval.

For peer review only

Reference

1. Qi XM, Luo Y, Song MY, et al. Pneumoconiosis: current status and future prospects. *Chinese medical journal* 2021;134(8):898-907. doi: 10.1097/cm9.0000000000001461 [published Online First: 2021/04/22]

2. Global, regional, and national incidence, prevalence, and years lived with disability for 354 diseases and injuries for 195 countries and territories, 1990-2017: a systematic analysis for the Global Burden of Disease Study 2017. *Lancet (London, England)* 2018;392(10159):1789-858. doi: 10.1016/s0140-6736(18)32279-7 [published Online First: 2018/11/30]

3. Global, regional, and national incidence, prevalence, and years lived with disability for 328 diseases and injuries for 195 countries, 1990-2016: a systematic analysis for the Global Burden of Disease Study 2016. *Lancet (London, England)* 2017;390(10100):1211-59. doi: 10.1016/s0140-6736(17)32154-2 [published Online First: 2017/09/19]

4. Global, regional, and national incidence, prevalence, and years lived with disability for 310 diseases and injuries, 1990-2015: a systematic analysis for the Global Burden of Disease Study 2015. *Lancet (London, England)* 2016;388(10053):1545-602. doi: 10.1016/s0140-6736(16)31678-6 [published Online First: 2016/10/14]

5. Global, regional, and national incidence, prevalence, and years lived with disability for 301 acute and chronic diseases and injuries in 188 countries, 1990-2013: a systematic analysis for the Global Burden of Disease Study 2013. *Lancet (London, England)* 2015;386(9995):743-800. doi: 10.1016/s0140-6736(15)60692-4 [published Online First: 2015/06/13]

For peer review only - http://bmjopen.bmj.com/site/about/guidelines.xhtml

6. Cui K, Shen F, Han B, et al. Comparison of the Cumulative Incidence Rates of Coal Workers' Pneumoconiosis between 1970 and 2013 among Four State-Owned Colliery Groups in China. *International journal of environmental research and public health* 2015;12(7):7444-56. doi: 10.3390/ijerph120707444 [published Online First: 2015/07/03]

7. Blackley DJ, Halldin CN, Laney AS. Continued Increase in Prevalence of Coal Workers' Pneumoconiosis in the United States, 1970-2017. *American journal of public health* 2018;108(9):1220-22. doi: 10.2105/ajph.2018.304517 [published Online First: 2018/07/20]

8. Poinen-Rughooputh S, Rughooputh MS, Guo Y, et al. Sex-Related Differences in the Risk of Silicosis Among Chinese Pottery Workers: A Cohort Study. *Journal of occupational and environmental medicine* 2021;63(1):74-79. doi: 10.1097/jom.0000000000002068 [published Online First: 2020/10/31]

9. Chen W, Hnizdo E, Chen JQ, et al. Risk of silicosis in cohorts of Chinese tin and tungsten miners, and pottery workers (I): an epidemiological study. *American journal of industrial medicine* 2005;48(1):1-9. doi: 10.1002/ajim.20174 [published Online First: 2005/06/09]

10. Han B, Liu H, Zhai G, et al. Estimates and Predictions of Coal Workers' Pneumoconiosis Cases among Redeployed Coal Workers of the Fuxin Mining Industry Group in China: A Historical Cohort Study. *PloS one* 2016;11(2):e0148179. doi: 10.1371/journal.pone.0148179 [published Online First: 2016/02/06]

11. Page MJ, McKenzie JE, Bossuyt PM, et al. The PRISMA 2020 statement: an updated guideline for reporting systematic reviews. *BMJ (Clinical research ed)* 2021;372:n71.

For peer review only

doi: 10.1136/bmj.n71 [published Online First: 2021/03/31]

12. Shipley RT. The 1980 ILO classification of radiographs of the pneumoconioses. *Radiologic clinics of North America* 1992;30(6):1135-45. [published Online First: 1992/11/01]

13. Muszyńska-Graca M, Dąbkowska B, Brewczyński PZ. [Guidelines for the use of the International Classification of Radiographs of Pneumoconioses of the International Labour Office (ILO): Substantial changes in the currrent edition]. *Medycyna pracy* 2016;67(6):833-37. doi: 10.13075/mp.5893.00493 [published Online First: 2016/12/23]

14. Gao YH, Wu J, Xing JC, et al. [Changes in diagnostic criteria for pneumoconiosis in China]. *Zhonghua lao dong wei sheng zhi ye bing za zhi = Zhonghua laodong weisheng zhiyebing zazhi = Chinese journal of industrial hygiene and occupational diseases* 2013;31(7):557-9. [published Online First: 2013/09/24]

15. Greenland S, Longnecker MP. Methods for trend estimation from summarized dose-response data, with applications to meta-analysis. *American journal of epidemiology* 1992;135(11):1301-9. doi: 10.1093/oxfordjournals.aje.a116237 [published Online First: 1992/06/01]

16. Orsini N, Li R, Wolk A, et al. Meta-analysis for linear and nonlinear dose-response relations: examples, an evaluation of approximations, and software. *American journal of epidemiology* 2012;175(1):66-73. doi: 10.1093/aje/kwr265 [published Online First: 2011/12/03]

17. Morfeld P, Mundt KA, Taeger D, et al. Threshold value estimation for respirable quartz dust exposure and silicosis incidence among workers in the german porcelain industry. *Journal of occupational and environmental medicine* 2013;55(9):1027-34. doi:

For peer review only - http://bmjopen.bmj.com/site/about/guidelines.xhtml

10.1097/JOM.0b013e318297327a [published Online First: 2013/08/24]

18. Zhang M, Zheng YD, Du XY, et al. Silicosis in automobile foundry workers: a 29-year cohort study. *Biomedical and environmental sciences : BES* 2010;23(2):121-9. doi: 10.1016/s0895-3988(10)60041-4 [published Online First: 2010/06/03]

19. Noweir MH. Some observations on epidemiologic studies in Egyptian gins, cotton pressing plants and cottonseed oil extraction plants. *Chest* 1981;79(4 Suppl):15s-20s. doi: 10.1378/chest.79.4_supplement.15s [published Online First: 1981/04/01]

20. Wang D, Zhou M, Liu Y, et al. Comparison of Risk of Silicosis in Metal Mines and Pottery Factories: A 44-Year Cohort Study. *Chest* 2020;158(3):1050-59. doi: 10.1016/j.chest.2020.03.054

21. Hnizdo E, Sluis-Cremer GK. Risk of silicosis in a cohort of white South African gold miners. *American journal of industrial medicine* 1993;24(4):447-57. doi: 10.1002/ajim.4700240409 [published Online First: 1993/10/01]

22. Guan HY, Zhang H, Su LP, et al. [The study of the characteristics and influencing factors of pneumoconiosis among workers exposed to dusts in an iron mine]. *Zhonghua lao dong wei sheng zhi ye bing za zhi = Zhonghua laodong weisheng zhiyebing zazhi = Chinese journal of industrial hygiene and occupational diseases* 2012;30(1):36-40. [published Online First: 2012/06/27]

23. Zhang H, Yang J, Qin Y, et al. [Assessment of risk for tin mine dust in Guangxi]. *Zhonghua lao dong wei sheng zhi ye bing za zhi = Zhonghua laodong weisheng zhiyebing zazhi = Chinese journal of industrial hygiene and occupational diseases* 2002;20(6):430-2. [published Online First: 2003/12/26]

For peer review only - http://bmjopen.bmj.com/site/about/guidelines.xhtml

24. Zhang G, Wang XT. [Dose-response relationship analysis between cumulative coal dust exposure and pneumoconiosis risk]. *Zhonghua lao dong wei sheng zhi ye bing za zhi = Zhonghua laodong weisheng zhiyebing zazhi = Chinese journal of industrial hygiene and occupational diseases* 2020;38(6):433-37. doi: 10.3760/cma.j.cn121094-20190510-00194 [published Online First: 2020/07/08]

25. Liu XG. [Study on the response index of population--the prevalence degree of pneumoconiosis]. *Zhonghua yu fang yi xue za zhi [Chinese journal of preventive medicine]* 1992;26(6):342-4. [published Online First: 1992/11/01]

26. Rosenman KD, Reilly MJ, Rice C, et al. Silicosis among foundry workers. Implication for the need to revise the OSHA standard. *American journal of epidemiology* 1996;144(9):890-900. doi: 10.1093/oxfordjournals.aje.a009023 [published Online First: 1996/11/01]

27. Liu H, Tang Z, Yang Y, et al. Identification and classification of high risk groups for Coal Workers' Pneumoconiosis using an artificial neural network based on occupational histories: a retrospective cohort study. *BMC public health* 2009;9:366. doi: 10.1186/1471-2458-9-366 [published Online First: 2009/09/30]

28. Shen F, Liu H, Yuan J, et al. Cost-Effectiveness of Coal Workers' Pneumoconiosis Prevention Based on Its Predicted Incidence within the Datong Coal Mine Group in China. *PloS one* 2015;10(6):e0130958. doi: 10.1371/journal.pone.0130958 [published Online First: 2015/06/23]

29. Shen F, Yuan J, Sun Z, et al. Risk identification and prediction of coal workers' pneumoconiosis in Kailuan Colliery Group in China: a historical cohort study. *PloS one*

For peer review only - http://bmjopen.bmj.com/site/about/guidelines.xhtml

2013;8(12):e82181. doi: 10.1371/journal.pone.0082181 [published Online First: 2014/01/01]

30. Steenland K, Brown D. Silicosis among gold miners: exposure--response analyses and risk assessment. *American journal of public health* 1995;85(10):1372-7. doi: 10.2105/ajph.85.10.1372 [published Online First: 1995/10/01]

31. Chen W, Zhuang Z, Attfield MD, et al. Exposure to silica and silicosis among tin miners in China: exposure-response analyses and risk assessment. *Occupational and environmental medicine* 2001;58(1):31-7. doi: 10.1136/oem.58.1.31 [published Online First: 2000/12/19]

32. Shi P, Xing X, Xi S, et al. Trends in global, regional and national incidence of pneumoconiosis caused by different aetiologies: an analysis from the Global Burden of Disease Study 2017. *Occupational and environmental medicine* 2020;77(6):407-14. doi: 10.1136/oemed-2019-106321 [published Online First: 2020/03/20]

33. Lu C, Dasgupta P, Cameron J, et al. A systematic review and meta-analysis on international studies of prevalence, mortality and survival due to coal mine dust lung disease. *PloS one* 2021;16(8):e0255617. doi: 10.1371/journal.pone.0255617 [published Online First: 2021/08/04]

34. Tan S, Chen S. Macrophage Autophagy and Silicosis: Current Perspective and Latest Insights. *International journal of molecular sciences* 2021;22(1) doi: 10.3390/ijms22010453 [published Online First: 2021/01/21]

35. Mayeux JM, Escalante GM, Christy JM, et al. Silicosis and Silica-Induced Autoimmunity in the Diversity Outbred Mouse. *Frontiers in immunology* 2018;9:874. doi:

For peer review only - http://bmjopen.bmj.com/site/about/guidelines.xhtml

10.3389/fimmu.2018.00874 [published Online First: 2018/05/15]

36. Tan S, Chen S. The Mechanism and Effect of Autophagy, Apoptosis, and Pyroptosis on the Progression of Silicosis. *International journal of molecular sciences* 2021;22(15) doi: 10.3390/ijms22158110 [published Online First: 2021/08/08]

37. Huang X, Li W, Attfield MD, et al. Mapping and prediction of coal workers' pneumoconiosis with bioavailable iron content in the bituminous coals. *Environmental health perspectives* 2005;113(8):964-8. doi: 10.1289/ehp.7679 [published Online First: 2005/08/05]

38. Suarthana E, Laney AS, Storey E, et al. Coal workers' pneumoconiosis in the United States: regional differences 40 years after implementation of the 1969 Federal Coal Mine Health and Safety Act. *Occupational and environmental medicine* 2011;68(12):908-13. doi: 10.1136/oem.2010.063594 [published Online First: 2011/05/21]

39. Mo J, Wang L, Au W, et al. Prevalence of coal workers' pneumoconiosis in China: a systematic analysis of 2001-2011 studies. *International journal of hygiene and environmental health* 2014;217(1):46-51. doi: 10.1016/j.ijheh.2013.03.006 [published Online First: 2013/04/24]

40. Forastiere F, Lagorio S, Michelozzi P, et al. Silica, silicosis and lung cancer among ceramic workers: a case-referent study. *American journal of industrial medicine* 1986;10(4):363-70. doi: 10.1002/ajim.4700100404 [published Online First: 1986/01/01]

41. Rastogi SK, Gupta BN, Chandra H, et al. A study of the prevalence of respiratory morbidity among agate workers. *International archives of occupational and environmental health* 1991;63(1):21-6. doi: 10.1007/bf00406193 [published Online First: 1991/01/01]

For peer review only - http://bmjopen.bmj.com/site/about/guidelines.xhtml

42. Gielec L, Izycki J, Woźniak H. [Evaluation of long-term occupational exposure to dust and its effect on health during production of ceramic tiles]. *Medycyna pracy* 1992;43(1):25-33. [published Online First: 1992/01/01]

43. Chaudhury N, Phatak A, Paliwal R, et al. Silicosis among agate workers at Shakarpur: An analysis of clinic-based data. *Lung India : official organ of Indian Chest Society* 2010;27(4):221-4. doi: 10.4103/0970-2113.71955 [published Online First: 2010/12/09]

44. Miller AE, MacDougall JD, Tarnopolsky MA, et al. Gender differences in strength and muscle fiber characteristics. *European journal of applied physiology and occupational physiology* 1993;66(3):254-62. doi: 10.1007/bf00235103 [published Online First: 1993/01/01]

45. Card JW, Zeldin DC. Hormonal influences on lung function and response to environmental agents: lessons from animal models of respiratory disease. *Proceedings of the American Thoracic Society* 2009;6(7):588-95. doi: 10.1513/pats.200904-020RM [published Online First: 2009/11/26]

46. An J, Ribeiro RC, Webb P, et al. Estradiol repression of tumor necrosis factor-alpha transcription requires estrogen receptor activation function-2 and is enhanced by coactivators. *Proceedings of the National Academy of Sciences of the United States of America* 1999;96(26):15161-6. doi: 10.1073/pnas.96.26.15161 [published Online First: 1999/12/28]

47. Grundboeck-Jusco J, Binette JP, Kimura A, et al. The glycosaminoglycan composition of the lung with acute and chronic pathology and in senescence. *Clinica chimica acta; international journal of clinical chemistry* 1992;208(1-2):77-84. doi: 10.1016/0009-

For peer review only - http://bmjopen.bmj.com/site/about/guidelines.xhtml

8981(92)90023-j [published Online First: 1992/06/15]

48. Wang D, Yang M, Liu Y, et al. Association of Silica Dust Exposure and Cigarette Smoking With Mortality Among Mine and Pottery Workers in China. *JAMA network open* 2020;3(4):e202787. doi: 10.1001/jamanetworkopen.2020.2787 [published Online First: 2020/04/15]

49. Sager TM, Umbright CM, Mustafa GM, et al. Tobacco Smoke Exposure Exacerbated Crystalline Silica-Induced Lung Toxicity in Rats. *Toxicological sciences : an official journal of the Society of Toxicology* 2020;178(2):375-90. doi: 10.1093/toxsci/kfaa146 [published Online First: 2020/09/26]

50. Dorman DC, Mokashi V, Wagner DJ, et al. Biological responses in rats exposed to cigarette smoke and Middle East sand (dust). *Inhalation toxicology* 2012;24(2):109-24. doi: 10.3109/08958378.2011.647413 [published Online First: 2012/01/31]

51. Dong J, Ma Q. Osteopontin enhances multi-walled carbon nanotube-triggered lung fibrosis by promoting TGF-β1 activation and myofibroblast differentiation. *Particle and fibre toxicology* 2017;14(1):18. doi: 10.1186/s12989-017-0198-0 [published Online First: 2017/06/10]

52. Mossman BT, Churg A. Mechanisms in the pathogenesis of asbestosis and silicosis. *American journal of respiratory and critical care medicine* 1998;157(5 Pt 1):1666-80. doi: 10.1164/ajrccm.157.5.9707141 [published Online First: 1998/05/29]

53. Fujimura N. Pathology and pathophysiology of pneumoconiosis. *Current opinion in pulmonary medicine* 2000;6(2):140-4. doi: 10.1097/00063198-200003000-00010 [published Online First: 2000/03/31]

For peer review only - http://bmjopen.bmj.com/site/about/guidelines.xhtml

54. McCunney RJ, Morfeld P, Payne S. What component of coal causes coal workers' pneumoconiosis? *Journal of occupational and environmental medicine* 2009;51(4):462-71. doi: 10.1097/JOM.0b013e3181a01ada [published Online First: 2009/04/01]

55. Castranova V, Vallyathan V. Silicosis and coal workers' pneumoconiosis. *Environmental health perspectives* 2000;108 Suppl 4(Suppl 4):675-84. doi: 10.1289/ehp.00108s4675 [published Online First: 2000/08/10]

56. Souza TP, Watte G, Gusso AM, et al. Silicosis prevalence and risk factors in semi-precious stone mining in Brazil. *American journal of industrial medicine* 2017;60(6):529-36. doi: 10.1002/ajim.22719 [published Online First: 2017/05/18]

57. Bevan R, Ashdown L, McGough D, et al. Setting evidence-based occupational exposure limits for manganese. *Neurotoxicology* 2017;58:238-48. doi: 10.1016/j.neuro.2016.08.005 [published Online First: 2016/08/16]

58. Buchanan D, Miller BG, Soutar CA. Quantitative relations between exposure to respirable quartz and risk of silicosis. *Occupational and environmental medicine* 2003;60(3):159-64. doi: 10.1136/oem.60.3.159 [published Online First: 2003/02/25]

59. Krefft S, Wolff J, Rose C. Silicosis: An Update and Guide for Clinicians. *Clinics in chest medicine* 2020;41(4):709-22. doi: 10.1016/j.ccm.2020.08.012 [published Online First: 2020/11/07]

For peer review only - http://bmjopen.bmj.com/site/about/guidelines.xhtml

Figure legend:

Fig. 1 Flow diagram of study selection

Fig. 2 The forest plot of pooled incidence of pneumoconiosis

Fig. 3 Forest plot detailing risk ratios for the influencing factors when comparing participants who developed pneumoconiosis with controls. “Yes” indicates cases who developed pneumoconiosis; “No” indicates cases who didn’t develop pneumoconiosis. (a) male VS female (RR: 3.73, 95%CI: 1.33-10.47) with the subgroup analysis of study quality (NOS<7 versus NOS≥7), (b) smoking VS nerve smoking (RR: 1.80, 95%CI: 1.34-2.43), (c) tunneling VS others (RR: 4.75, 95%CI: 1.99-13.33), (d) helping VS others (RR: 0.07, 95%CI: 0.03=0.16).

Fig. 4 The association between the risk of pneumoconiosis and dust exposure. The black line indicates the linear model; the sky-blue line indicates the spline model; the dashed lines represent 95% confidence intervals. (a) The dose-response association between the duration of dust exposure and the risk of pneumoconiosis (P-nonlinearity: 0.10), (b) The dose-response association between the CTD and the risk of pneumoconiosis (P-nonlinearity: 0.16), (c) Forest plot detailing risk ratios for each 5-year increase in the duration of dust exposure (RR: 1.26, 95%CI: 1.15-1.39), (d) Forest plot detailing risk ratios for each 50 mg/m^3-years increase in CTD (RR: 1.38, 95%CI: 1.38-1.55). ES: effect size; CTD: Cumulative dust concentration.

Fig. 5 Correlation between Cumulative Silica Exposure (CSE) and incidence of silicosis. Concentrations of CSE and the incidence were highly correlated (r=0.794, P<0.001). Y scales are logarithmically transformed.

Supplementary Fig. 1 The forest plot of sensitivity results for pooling incidence of pneumoconiosis.

For peer review only - http://bmjopen.bmj.com/site/about/guidelines.xhtml

Supplementary Fig. 2 The funnel plot of publication bias of included papers for pooling incidence of pneumoconiosis.

Supplementary Fig. 3 Forest plot for summary of relative risk of pneumoconiosis for longest versus shortest duration of dust exposure (RR: 4.59, 95%CI: 2.41-8.74). ES: effect size.

Supplementary Fig. 4 Forest plot for a summary of relative risk of pneumoconiosis for highest versus lowest categories of cumulative dust concentration (CTD) (RR: 34.14, 95%CI: 17.50-66.63). ES: effect size.

Supplementary Fig. 5 The sensitivity results for the dose-response analysis between the cumulative dust concentration (CTD) and the risk of pneumoconiosis (P-linearity: 0.74).

Supplementary Fig. 6 The forest plot of sensitivity results for highest versus lowest categories of cumulative dust concentration (CTD) (RR: 46.74, 95%CI: 27.61-79.11). ES: effect size.

Supplementary Fig. 7 The sensitivity results for a summary of the relative risk of pneumoconiosis for each 50 mg/m^3-years increase in cumulative dust concentration (CTD) (RR: 1.34, 95%CI: 1.18-1.52). ES: effect size.

For peer review only

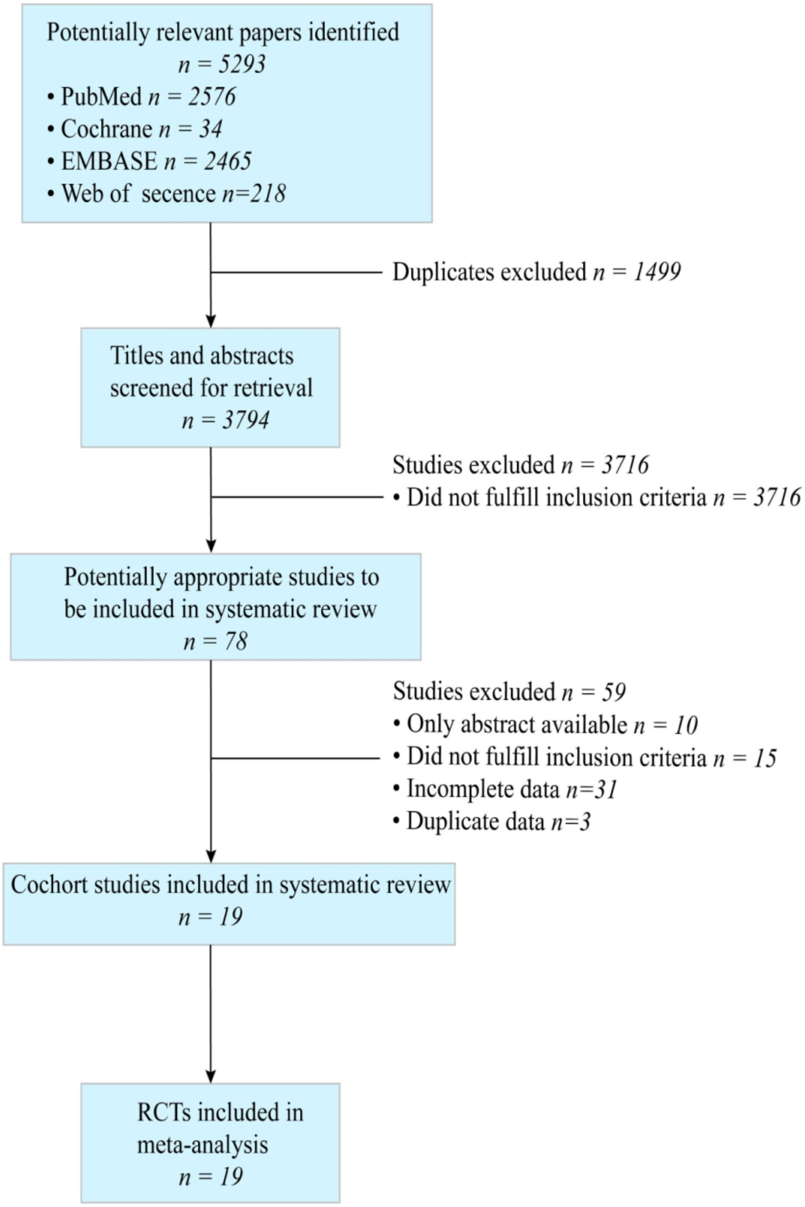

Flow diagram of study selection

100x150mm (300 x 300 DPI)

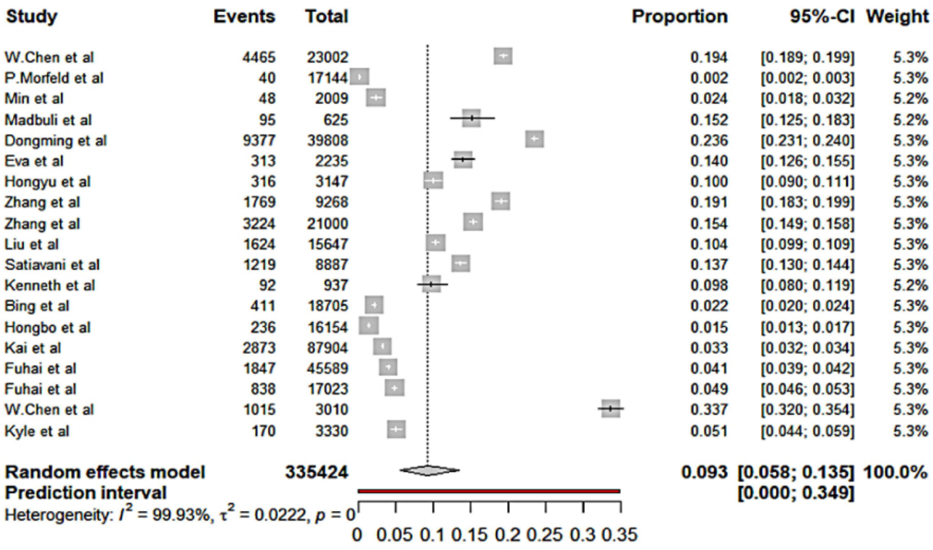

The forest plot of pooled incidence of pneumoconiosis

80x45mm (300 x 300 DPI)

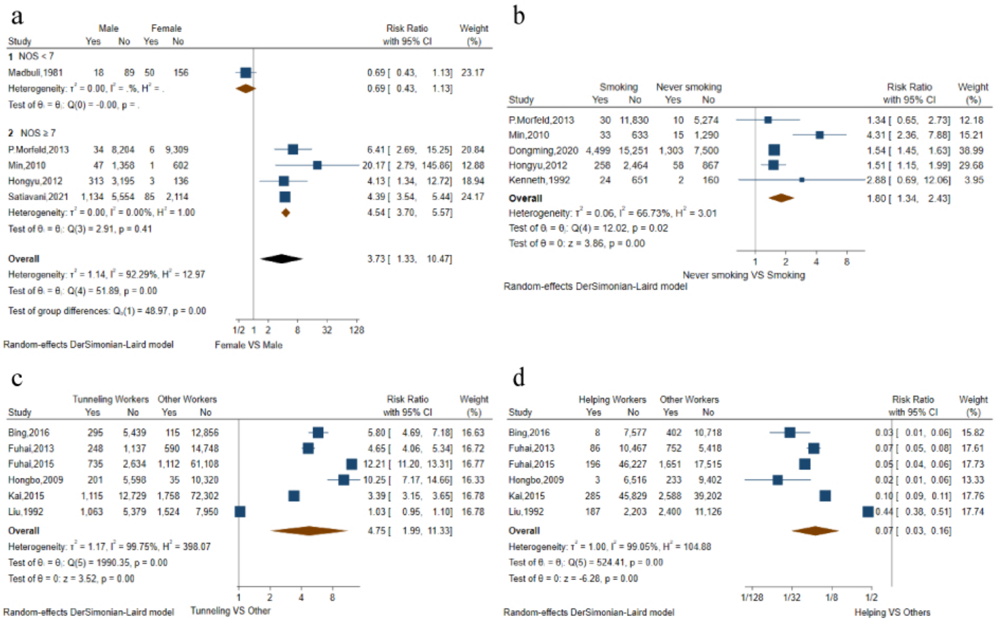

Forest plot detailing risk ratios for the influencing factors when comparing participants who developed pneumoconiosis with controls. “Yes” indicates cases who developed pneumoconiosis; “No” indicates cases who didn’t develop pneumoconiosis. (a) male VS female (RR: 3.73, 95%CI: 1.33-10.47) with the subgroup analysis of study quality (NOS<7 versus NOS≥7), (b) smoking VS nerve smoking (RR: 1.80, 95%CI: 1.34-2.43), (c) tunneling VS others (RR: 4.75, 95%CI: 1.99-13.33), (d) helping VS others (RR: 0.07, 95%CI: 0.03=0.16).

80x49mm (300 x 300 DPI)

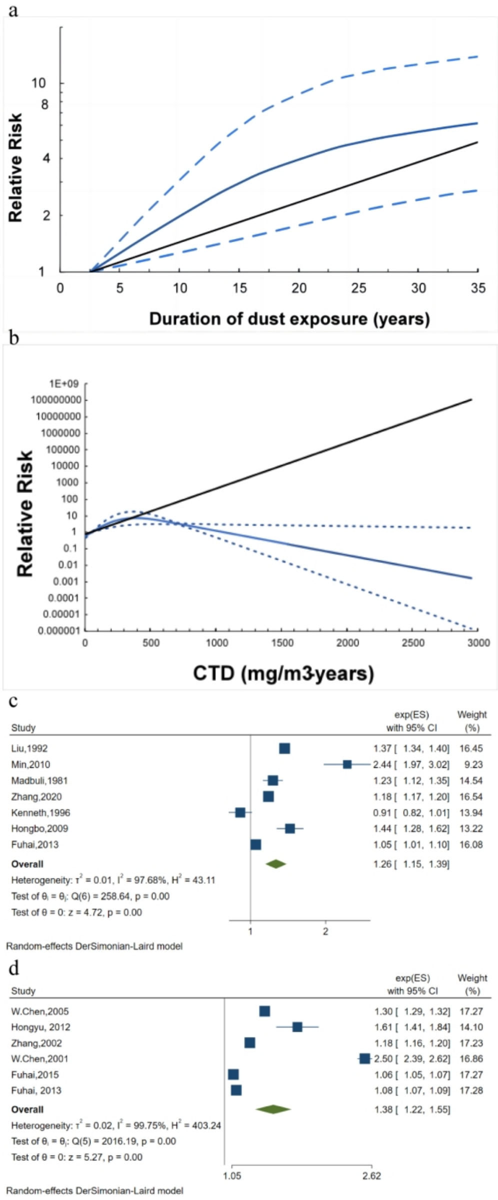

The association between the risk of pneumoconiosis and dust exposure. The black line indicates the linear model; the sky-blue line indicates the spline model; the dashed lines represent 95% confidence intervals. (a) The dose-response association between the duration of dust exposure and the risk of pneumoconiosis (P-nonlinearity: 0.10), (b) The dose-response association between the CTD and the risk of pneumoconiosis (P-nonlinearity: 0.16), (c) Forest plot detailing risk ratios for each 5-year increase in the duration of dust exposure (RR: 1.26, 95%CI: 1.15-1.39), (d) Forest plot detailing risk ratios for each 50 mg/m3-years increase in CTD (RR: 1.38, 95%CI: 1.38-1.55). ES: effect size; CTD: Cumulative dust concentration.

80x193mm (300 x 300 DPI)

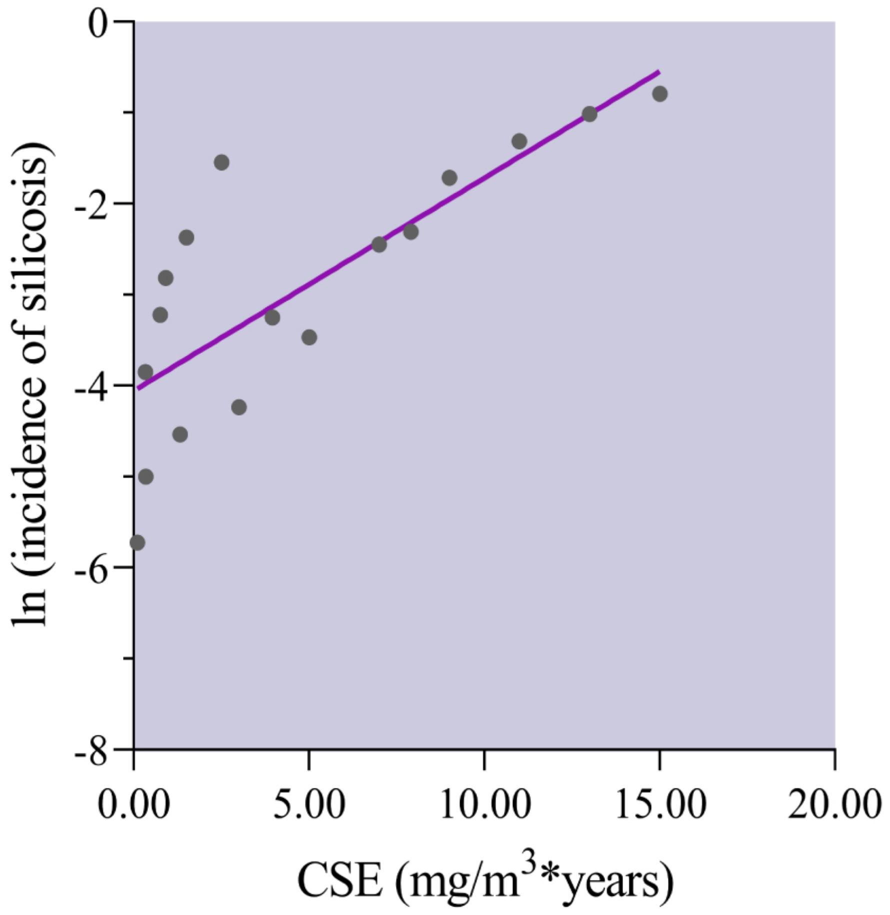

Correlation between Cumulative Silica Exposure (CSE) and incidence of silicosis. Concentrations of CSE and the incidence were highly correlated (r=0.794, P<0.001). Y scales are logarithmically transformed.

80x80mm (300 x 300 DPI)

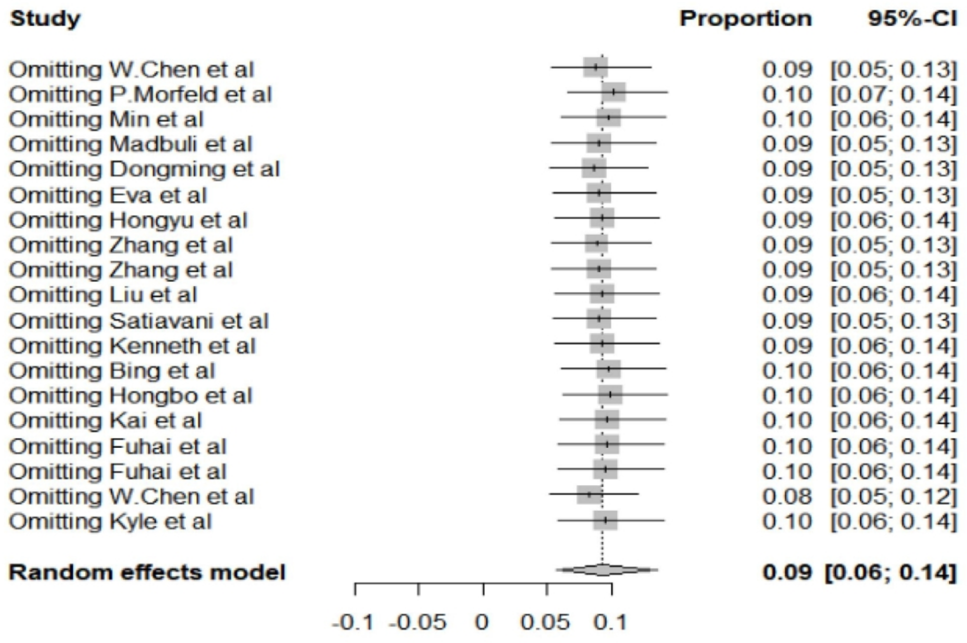

The forest plot of sensitivity results for pooling incidence of pneumoconiosis.

527x352mm (96 x 96 DPI)

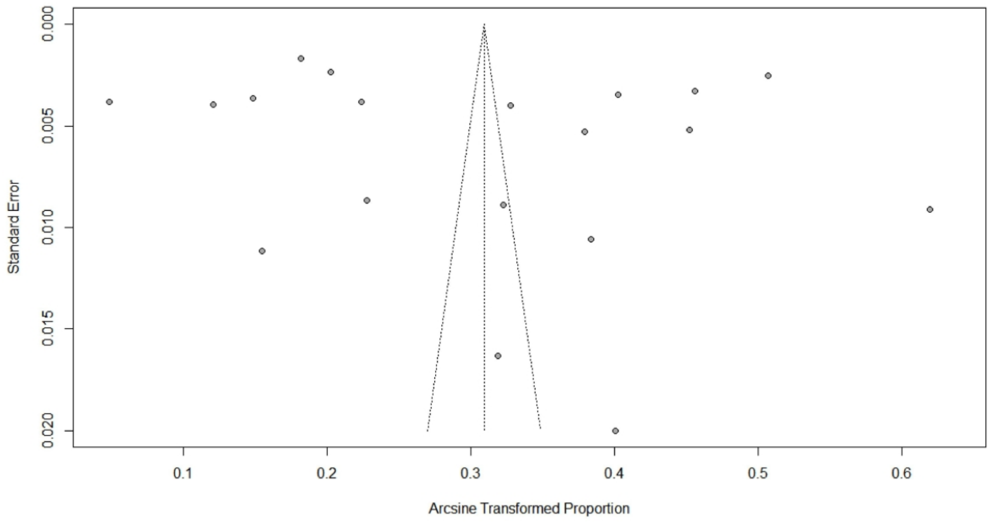

The funnel plot of publication bias of included papers for pooling incidence of pneumoconiosis.

914x489mm (96 x 96 DPI)

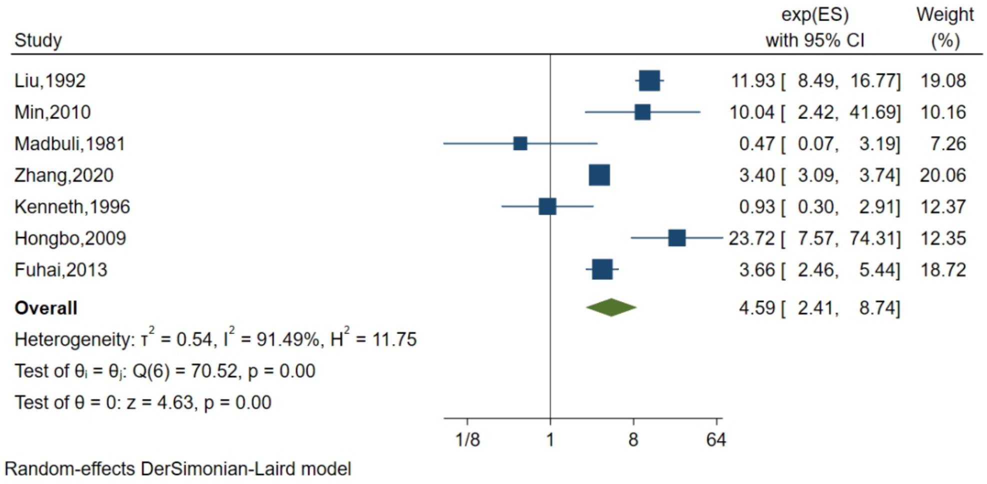

Forest plot for summary of relative risk of pneumoconiosis for longest versus shortest duration of dust exposure (RR: 4.59, 95%CI: 2.41-8.74). ES: effect size.

435x216mm (72 x 72 DPI)

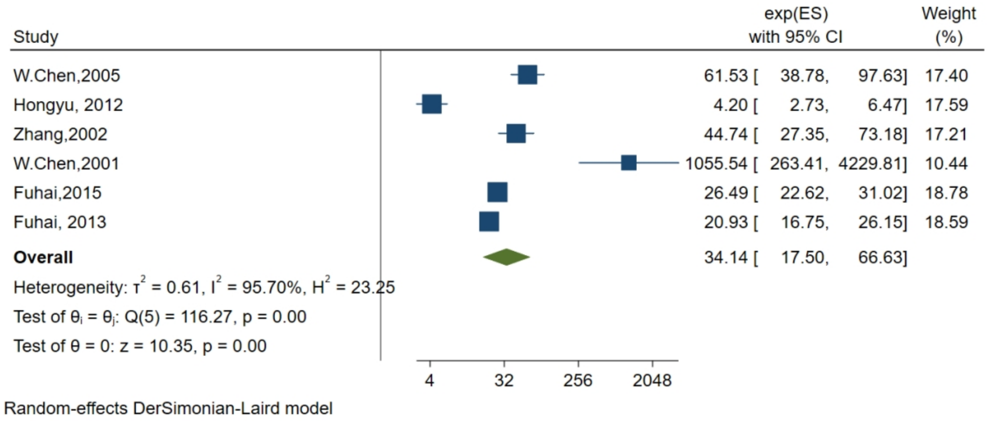

Forest plot for a summary of relative risk of pneumoconiosis for highest versus lowest categories of cumulative dust concentration (CTD) (RR: 34.14, 95%CI: 17.50-66.63). ES: effect size.

480x209mm (72 x 72 DPI)

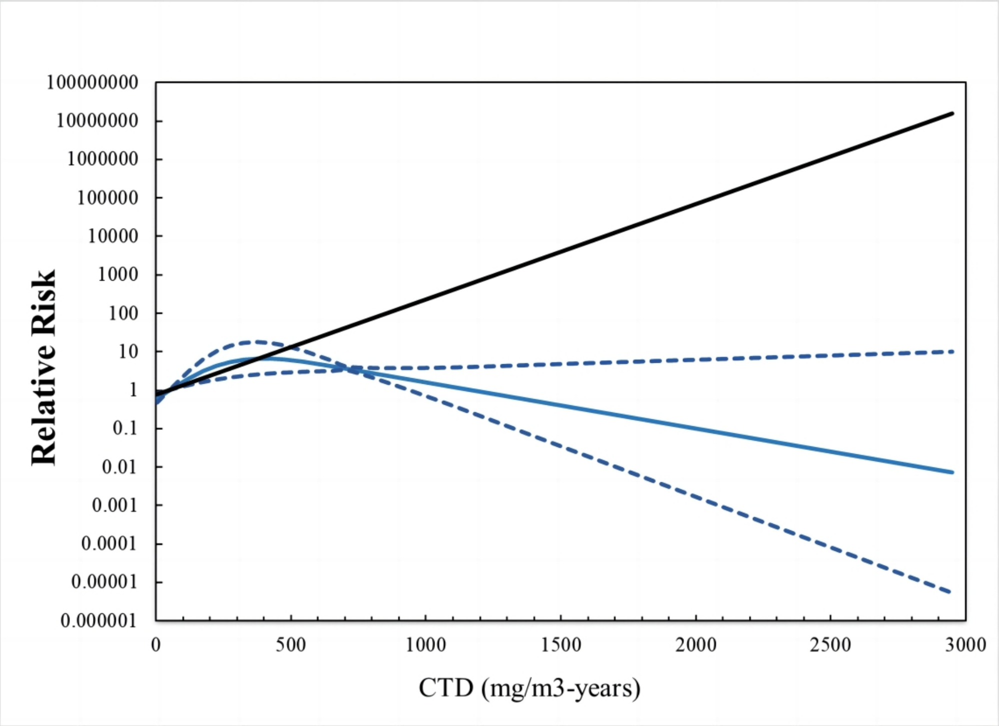

The sensitivity results for the dose-response analysis between the cumulative dust concentration (CTD) and the risk of pneumoconiosis (P-linearity: 0.74).

1275x926mm (96 x 96 DPI)

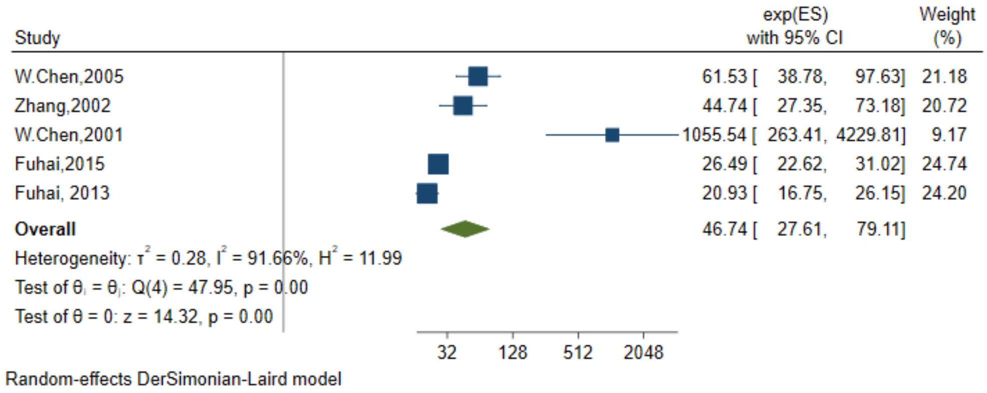

The forest plot of sensitivity results for highest versus lowest categories of cumulative dust concentration (CTD) (RR: 46.74, 95%CI: 27.61-79.11). ES: effect size.

254x103mm (72 x 72 DPI)

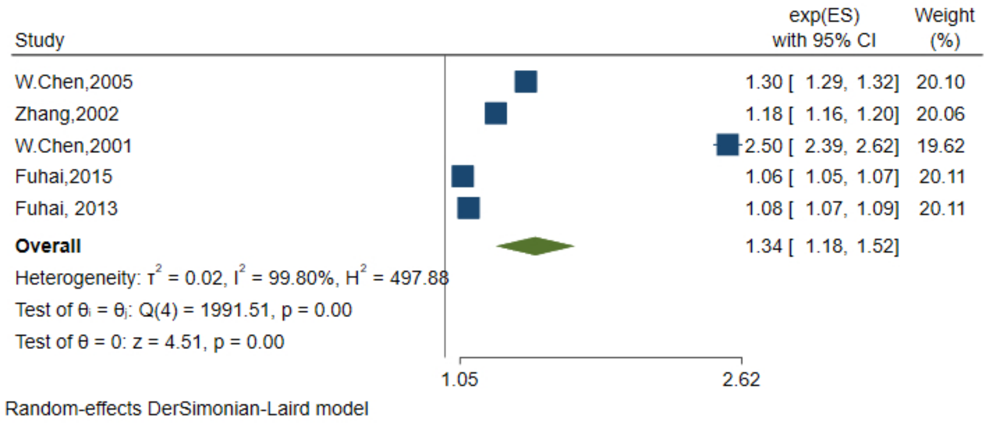

The sensitivity results for a summary of the relative risk of pneumoconiosis for each 50 mg/m3-years increase in cumulative dust concentration (CTD) (RR: 1.34, 95%CI: 1.18-1.52). ES: effect size.

253x110mm (72 x 72 DPI)

Search strategy of each online database

Online database	Search number	Search strategy
PubMed	#1	Silicosis [MeSH Terms] OR anthracosis [MeSH Terms] OR anthracosilicosis [MeSH Terms] OR berylliosis [MeSH Terms] OR byssinosis [MeSH Terms] OR siderosis [MeSH Terms] OR Caplan syndrome [MeSH Terms]
	#2	Pneumoconios*[Title/Abstract] OR pneumokoniosis [Title/Abstract] OR asbestos*[Title/Abstract] OR silicosis [Title/Abstract] OR anthracosis [Title/Abstract] OR anthracosilicosis [Title/Abstract] OR berylliosis [Title/Abstract] OR byssinosis [Title/Abstract] OR siderosis [Title/Abstract] OR "Caplan syndrome"[Title/Abstract] OR "Caplan's syndrome" [Title/Abstract] OR aluminosis [Title/Abstract] OR baritosis [Title/Abstract] OR chalicosis [Title/Abstract] OR "occupational lung disease*" [Title/Abstract] OR "dust disease*" [Title/Abstract]
	#3	(Silica[Title/Abstract] OR asbestos[Title/Abstract] OR cotton[Title/Abstract] OR

For peer review only - http://bmjopen.bmj.com/site/about/guidelines.xhtml

		bagasse[Title/Abstract] OR iron[Title/Abstract] OR anthracite[Title/Abstract] OR stone[Title/Abstract] OR barium [Title/Abstract] OR aluminum[Title/Abstract] OR beryllium[Title/Abstract] OR "hard metal"[Title/Abstract]) AND (dust*[Title/Abstract] OR particle*[Title/Abstract]) AND (lung[Title/Abstract] OR pleura*[Title/Abstract] OR "respiratory tract"[Title/Abstract] OR pulmonary[Title/Abstract] OR fibrosis[Title/Abstract])
	#4	#1 OR #2 OR #3
	#5	“Cohort studies” [MeSH Terms] OR “longitudinal studies” [MeSH Terms] OR “follow up studies” [MeSH Terms] OR “prospective studies” [MeSH Terms] OR “retrospective studies” [MeSH Terms]
	#6	“Cohort studies” [Title/Abstract] OR “longitudinal studies” [Title/Abstract] OR “follow up studies” [Title/Abstract] OR “prospective studies” [Title/Abstract] OR “retrospective studies” [Title/Abstract]
	#7	#5 OR #6

For peer review only

	#8	#4 AND #7
Cochrane Library	#1	(pneumoconios*):ti,ab,kw OR (pneumokoniosis):ti,ab,kw OR (asbestos*):ti,ab,kw OR (silicosis):ti,ab,kw OR (anthracosis):ti,ab,kw OR (anthracosilicosis):ti,ab,kw OR (berylliosis):ti,ab,kw OR (byssinosis):ti,ab,kw OR (siderosis):ti,ab,kw OR ("caplan syndrome"):ti,ab,kw OR ("caplan's syndrome"):ti,ab,kw OR (aluminosis):ti,ab,kw OR (baritosis):ti,ab,kw OR (chalicosis):ti,ab,kw OR ("occupational lung disease*"):ti,ab,kw OR ("dust disease*"):ti,ab,kw
	#2	MeSH descriptor: [Asbestosis] explode all trees
	#3	MeSH descriptor: [Silicosis] explode all trees
	#4	MeSH descriptor: [Anthracosis] explode all trees
	#5	MeSH descriptor: [Anthracosilicosis] explode all trees
	#6	MeSH descriptor: [Berylliosis] explode all trees
	#7	MeSH descriptor: [Byssinosis] explode all trees
	#8	MeSH descriptor: [Siderosis] explode all trees
	#9	MeSH descriptor: [Caplan Syndrome] explode all trees

For peer review only

	#10	#2 OR #3 OR #4 OR #5 OR #6 OR #7 OR #8
	#11	(silica):ti,ab,kw OR (asbestos):ti,ab,kw OR (cotton):ti,ab,kw OR (bagasse):ti,ab,kw OR (iron):ti,ab,kw OR (anthracite):ti,ab,kw OR (stone):ti,ab,kw OR (barium):ti,ab,kw OR (aluminium):ti,ab,kw OR (beryllium):ti,ab,kw OR ("hard metal"):ti,ab,kw
	#12	(dust*):ti,ab,kw OR (particle*):ti,ab,kw
	#13	(lung):ti,ab,kw OR (pleura*):ti,ab,kw OR ("respiratory tract"):ti,ab,kw OR (pulmonary):ti,ab,kw OR (fibrosis):ti,ab,kw
	#14	#11 AND #12 AND #13
	#15	#1 OR #10 OR #14
	#16	("cohort studies"):ti,ab,kw OR ("longitudinal studies"):ti,ab,kw OR ("follow up studies"):ti,ab,kw OR ("prospective studies"):ti,ab,kw OR ("retrospective studies"):ti,ab,kw
	#17	MeSH descriptor: [Retrospective Studies] explode all trees
	#18	MeSH descriptor: [Prospective Studies] explode all trees

For peer review only

	#19	MeSH descriptor: [Follow-Up Studies] explode all trees
	#20	MeSH descriptor: [Longitudinal Studies] explode all trees
	#21	MeSH descriptor: [Cohort Studies] explode all trees
	#22	#16 OR #17 OR #18 OR #19 OR #21
	#23	#15 AND #22
Web of Science	#1	TS = (Pneumoconios* OR pneumokoniosis OR asbestos* OR silicosis OR anthracosis OR anthracosilicosis OR berylliosis OR byssinosis OR siderosis OR Caplan* syndrome OR aluminosis OR baritosis OR chalicosis OR occupational lung disease* OR dust disease*)
	#2	TS = (Silica OR asbestos OR cotton OR bagasse OR iron OR anthracite OR stone OR barium OR aluminum OR beryllium OR hard metal)
	#3	TS = (dust* OR particle*)
	#4	TS = (lung OR pleura* OR respiratory tract OR pulmonary OR fibrosis)
	#5	#2 AND #3 AND #4
	#6	#1 OR #5
	#7	TS = (cohort studies OR longitudinal studies OR

For peer review only

		follow up studies OR prospective studies OR retrospective studies)
	#8	#6 AND #7
Embase	#1	'siderosis'/exp OR 'occupational lung disease'/exp
	#2	pneumoconios*: ab,ti OR pneumokoniosis: ab,ti OR asbestos*: ab,ti OR silicosis: ab,ti OR anthracosis: ab,ti OR anthracosilicosis: ab,ti OR berylliosis: ab,ti OR byssinosis: ab,ti OR siderosis: ab,ti OR 'caplan* syndrome ': ab,ti OR aluminosis: ab,ti OR baritosis: ab,ti OR chalicosis: ab,ti OR 'occupational lung disease* ': ab,ti OR 'dust disease* ': ab,ti
	#3	silica: ab,ti OR asbestos: ab,ti OR cotton: ab,ti OR bagasse: ab,ti OR iron: ab,ti OR anthracite: ab,ti OR stone: ab,ti OR barium: ab,ti OR aluminum: ab,ti OR beryllium: ab,ti OR 'hard metal ': ab,ti
	#4	dust*: ab,ti OR particle*: ab,ti
	#5	lung: ab,ti OR pleura*: ab,ti OR 'respiratory tract ': ab,ti OR pulmonary: ab,ti OR fibrosis: ab,ti
	#6	#3 AND #4 AND #5
	#7	#1 OR #2 OR #6
	#8	'longitudinal study'/exp OR 'cohort analysis'/exp OR

For peer review only

		'prospective study'/exp OR 'retrospective study'/exp
	#9	'cohort studies': ab,ti OR 'longitudinal studies': ab,ti OR 'follow up studies': ab,ti OR 'prospective studies': ab,ti OR 'retrospective studies': ab,ti
	#10	#8 OR #9
	#11	#7 AND #10

For peer review only

The detailed description of the analysis process (*e.g., CTD and the risk of pneumoconiosis*)

There was a risk ratio nonreferent log relative risks *log RR* corresponding to doses (CTD) for each study (a total of six studies were included in this analysis), which are normally taken at the midpoint of each exposure group's range (just like the description in method section).

The data is then centralized, which is done by subtracting the values of the other central points in each study from the values of the smallest central points, which is defined as *CTDC*.

Following that, a two-stage random-effects dose-response meta-analysis was used to examine a possible non-linear relationship between CTD and the risk of pneumoconiosis. When a common 3-knot (10%, 50%, and 90% of the distribution) restricted cubic spline transformation is performed to the vector of aggregated exposure data (containing the reference category midpoint), a matrix of two spline transformations and two variables, *CTDCS1* and *CTDCS2*, are generated. First, we used a generalized least squares trend estimation method to produce study-specific estimates under a constrained cubic spline model. Second, we pooled study-specific results in a multivariate random-effects model using the restricted maximum likelihood method.

The first variable, *CTDCS1*, in the restricted cubic spline model, is defined as linear components according to the principle of building nonlinear components in restricted cubic spline models. Furthermore, the nonlinear component of the restricted cubic spline model is the overall other nonlinear variables. Only one variable, *CTDCS2*, is a nonlinear component in this analysis. As a result, we determine if CTDCS2 is statistically significant in this analysis by computing the p-value of the invalid null hypothesis test for *CTDCS2*, in which *CTDCS2* was assumed to be equal to 0. (a linear dose-response analysis). With the help of stata software calculation, the P value can be obtained as 0.16. Therefore, statistically, it is accepted that the *CTDCS2* is equal to 0, that is, there is a linear dose-response relationship between the variables.

For peer review only

Using the two-stage generalized least squares trend estimation method, a linear dose-response connection between an additional 1 mg/m3-years and the risk was studied. Same as the above procedure. First, Study-specific slope lines were computed, and then these lines were pooled to give an overall average slope.

Finally, using software, we calculated the regression coefficients of the risk ratio at the chosen reference level, *RRs* (relative risks for the spline models), and *RRl* (relative risks for the lines models) corresponding to the reference exposure level, as well as the 95%CIs (*lbs, ubs*, and *lbl, ubl*) of each level exposure level under the spline and linear models, respectively, and polt them. It is worth noting that we picked 50 mg/m3-years as the reference exposure level because this is the generally accepted amplitude of CTD alterations. As a result, rather than a nonlinear dose-response connection, there is a straight dose-response association between CTD and the risk of pneumoconiosis.

We sought to further quantify this link when the results revealed a linear dose-response relationship between CTD and the risk of pneumoconiosis. The initial outcome of the dose-response study, however, was a change in the risk of pneumoconiosis for every 1 mg/m^3-year change in CTD, and a change of 1 mg/m^3-years was too tiny for CTD. For the reasons stated in the discussion section, we selected to calculate the change in risk of pneumoconiosis for every 50 mg/m^3-year rise in CTD. According to our study and calculations, the risk of pneumoconiosis increases by 1.38 (95% CI: 1.22-1.55) with every 50 mg/m^3-year increase in CTD. The forest plot in *Fig. 5a* depicted the change in pneumoconiosis risk for every 50 mg/m^3-year rise in CTD in each study, as well as the overall results using this linear dose-response model.

PRISMA 2020 Checklist

Section and Topic	Item #	Checklist item	Location where item is reported
**TITLE**			
Title	1	Identify the report as a systematic review.	p.1-lines1~2
**ABSTRACT**			
Abstract	2	See the PRISMA 2020 for Abstracts checklist.	p.2~p.3
**INTRODUCTION**			
Rationale	3	Describe the rationale for the review in the context of existing knowledge.	p.5~p.6-line9
Objectives	4	Provide an explicit statement of the objective(s) or question(s) the review addresses.	p.6-lines10~14
**METHODS**			
Eligibility criteria	5	Specify the inclusion and exclusion criteria for the review and how studies were grouped for the syntheses.	p.7-lines 6~22
Information sources	6	Specify all databases, registers, websites, organisations, reference lists and other sources searched or consulted to identify studies. Specify the date when each source was last searched or consulted.	p.6-line20~p.7-line 1
Search strategy	7	Present the full search strategies for all databases, registers and websites, including any filters and limits used.	p.7-lines 1~4
Selection process	8	Specify the methods used to decide whether a study met the inclusion criteria of the review, including how many reviewers screened each record and each report retrieved, whether they worked independently, and if applicable, details of automation tools used in the process.	p.6-line20~p.7 line3
Data collection process	9	Specify the methods used to collect data from reports, including how many reviewers collected data from each report, whether they worked independently, any processes for obtaining or confirming data from study investigators, and if applicable, details of automation tools used in the process.	p.8-lines 9~10
Data items	10a	List and define all outcomes for which data were sought. Specify whether all results that were compatible with each outcome domain in each study were sought (e.g. for all measures, time points, analyses), and if not, the methods used to decide which results to collect.	p.8-lines11~15
	10b	List and define all other variables for which data were sought (e.g. participant and intervention characteristics, funding sources). Describe any assumptions made about any missing or unclear information.	p.8-lines11~15
Study risk of bias assessment	11	Specify the methods used to assess risk of bias in the included studies, including details of the tool(s) used, how many reviewers assessed each study and whether they worked independently, and if applicable, details of automation tools used in the process.	p.11-lines6~8
Effect measures	12	Specify for each outcome the effect measure(s) (e.g. risk ratio, mean difference) used in the synthesis or presentation of results.	p.8-line22~p.9 line6
Synthesis methods	13a	Describe the processes used to decide which studies were eligible for each synthesis (e.g. tabulating the study intervention characteristics and comparing against the planned groups for each synthesis (item #5)).	p.7-lines 6~22, p.9-lines2~4, p.9-lines7~9.
	13b	Describe any methods required to prepare the data for presentation or synthesis, such as handling of missing summary statistics, or data conversions.	p.8-lines 9~10
	13c	Describe any methods used to tabulate or visually display results of individual studies and syntheses.	p.8-line 19~p.9-line 10
	13d	Describe any methods used to synthesize results and provide a rationale for the choice(s). If meta-analysis was performed, describe the model(s), method(s) to identify the presence and extent of statistical heterogeneity, and software package(s) used.	p.8-line19~p.10-line 20
	13e	Describe any methods used to explore possible causes of heterogeneity among study results (e.g. subgroup analysis, meta-regression).	p.10-line22~p.11-line5
	13f	Describe any sensitivity analyses conducted to assess robustness of the synthesized results.	p.10-lines 21~22
Reporting bias assessment	14	Describe any methods used to assess risk of bias due to missing results in a synthesis (arising from reporting biases).	p.11-lines 6~8
Certainty	15	Describe any methods used to assess certainty (or confidence) in the body of evidence for an outcome.	p.8-lines 2~7

For peer review only

PRISMA 2020 Checklist

Section and Topic	Item #	Checklist item	Location where item is reported
assessment			
**RESULTS**			
Study selection	16a	Describe the results of the search and selection process, from the number of records identified in the search to the number of studies included in the review, ideally using a flow diagram.	p.11-lines 14~20
	16b	Cite studies that might appear to meet the inclusion criteria, but which were excluded, and explain why they were excluded.	p.11-lines 17~19
Study characteristics	17	Cite each included study and present its characteristics.	p.11-line21~p.14 line3
Risk of bias in studies	18	Present assessments of risk of bias for each included study.	p.14-lines 9~11
Results of individual studies	19	For all outcomes, present, for each study: (a) summary statistics for each group (where appropriate) and (b) an effect estimate and its precision (e.g. confidence/credible interval), ideally using structured tables or plots.	p.14-line4~p.16-line5
Results of syntheses	20a	For each synthesis, briefly summarise the characteristics and risk of bias among contributing studies.	p.14-line4~p.19-line21
	20b	Present results of all statistical syntheses conducted. If meta-analysis was done, present for each the summary estimate and its precision (e.g. confidence/credible interval) and measures of statistical heterogeneity. If comparing groups, describe the direction of the effect.	p.14-line4~p.19-line21
	20c	Present results of all investigations of possible causes of heterogeneity among study results.	p.14-line4~p.19-line21
	20d	Present results of all sensitivity analyses conducted to assess the robustness of the synthesized results.	p.9-lines 9~10<br>p.1- lines 9~15
Reporting biases	21	Present assessments of risk of bias due to missing results (arising from reporting biases) for each synthesis assessed.	p.14-lines10~12
Certainty of evidence	22	Present assessments of certainty (or confidence) in the body of evidence for each outcome assessed.	p.11-line21~p.12-line 1
**DISCUSSION**			
Discussion	23a	Provide a general interpretation of the results in the context of other evidence.	p.20-lines1~11
	23b	Discuss any limitations of the evidence included in the review.	p.25-lines18~22
	23c	Discuss any limitations of the review processes used.	p.25-lines18~22
	23d	Discuss implications of the results for practice, policy, and future research.	p.20-line1~p.25-line 16
**OTHER INFORMATION**			
Registration and protocol	24a	Provide registration information for the review, including register name and registration number, or state that the review was not registered.	p.6-lines16~18
	24b	Indicate where the review protocol can be accessed, or state that a protocol was not prepared.	p.6-lines16~18
	24c	Describe and explain any amendments to information provided at registration or in the protocol.	p.6-lines16~18
Support	25	Describe sources of financial or non-financial support for the review, and the role of the funders or sponsors in the review.	p.26-lines19~22
Competing interests	26	Declare any competing interests of review authors.	p.27-line 1
Availability of data, code and	27	Report which of the following are publicly available and where they can be found: template data collection forms; data extracted from included studies; data used for all analyses; analytic code; any other materials used in the review.	p.27-lines 3~4

For peer review only

PRISMA 2020 Checklist

Section and Topic	Item #	Checklist item	Location where item is reported
other materials			

From: Page MJ, McKenzie JE, Bossuyt PM, Boutron I, Hoffmann TC, Mulrow CD, et al. The PRISMA 2020 statement: an updated guideline for reporting systematic reviews. BMJ 2021;372:n71. doi: 10.1136/bmj.n71

For more information, visit: http://www.prisma-statement.org/

For peer review only
